# Cyclodimers and Cyclotrimers of 2,3 ‐Bisalkynylated Anthracenes, Phenazines and Diazatetracenes

**DOI:** 10.1002/chem.202103193

**Published:** 2021-11-05

**Authors:** Steffen Maier, Nikolai Hippchen, Frank Rominger, Jan Freudenberg, Uwe H. F. Bunz

**Affiliations:** ^1^ Organisch-Chemisches Institut Ruprecht-Karls-Universität Heidelberg Im Neuenheimer Feld 270 69120 Heidelberg Germany; ^2^ Centre for Advanced Materials Ruprecht-Karls-Universität Heidelberg Im Neuenheimer Feld 225 69120 Heidelberg Germany

**Keywords:** acenes, alkynes, Glaser–Hay coupling, graphdyine, organic field-effect transistors

## Abstract

The synthesis of novel (N−)acene‐based cyclooligomers is reported. Glaser‐Hay coupling of the bisethynylated monomers results in cyclodimers and cyclotrimers that are separable by column and gel‐permeation chromatographies. For the diazatetracene, the use of *sec*‐butyl‐silylethynyl groups is necessary to achieve solubility. Diazatetracene‐based cyclodimers and cyclotrimers were used as semiconductors in thin‐film transistors. Although their optoelectronic properties are quite similar, their electron mobilities in proof‐of‐concept thin‐film transistors differ by an order of magnitude.

## Graphical abstract

**Upwardly mobile**: Glaser‐Hay coupling of *ortho*‐bisethynylated (aza)acenes furnishes Eglinton‐Gailbrath‐type cyclodimers and cyclotrimers. Although conjugation between the chromophores is poor through the diyne bridges, as evidenced by UV‐vis spectroscopy, the azatetracene‐based trimer exhibits electron mobilities one order of magnitude higher than its dimeric congener.

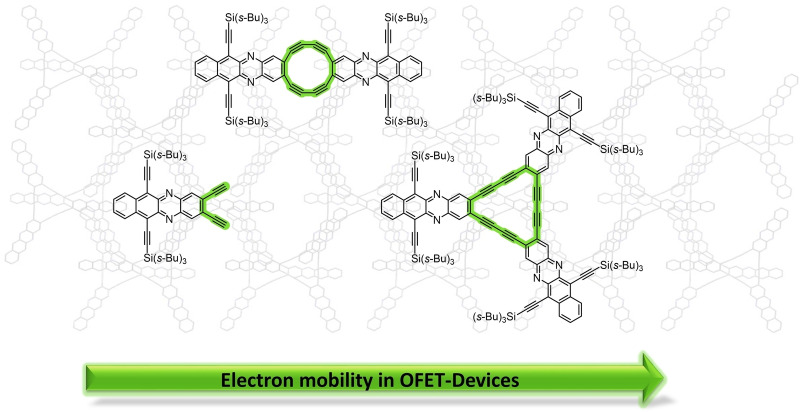

The reaction of diethynylbenzene with Cu(OAc)_2_ under oxidative conditions, published by Eglinton and Galbraith, selectively furnished the fairly angle deformed cyclodimer **A** (Figure 1),[^1^, ^2^] which polymerizes in the solid state.^[2]^ Later Swager et al.^[3]^ demonstrated that the apparent absence of the expected, less‐strained cyclotrimer was due to its insolubility ‐ alkoxy‐substituted diethynylbenzenes *do* give cyclotrimers in good yield upon oxidative homocoupling.^[4]^ Haley et al.^[5]^ published a series of elegant papers in which they connected such cyclotrimers into larger segments of a carbon allotrope called graphdiyne. Yet, only benzene rings^[6]^ were investigated until, in 1998, Komatsu published the dimerization of diethynylnaphthalene derivatives furnishing **B**,^[7]^ and Anthony prepared structure **C** as the cyclotrimer of a substituted 2,3‐diethynylpentacene.^[8]^

**Figure 1:**
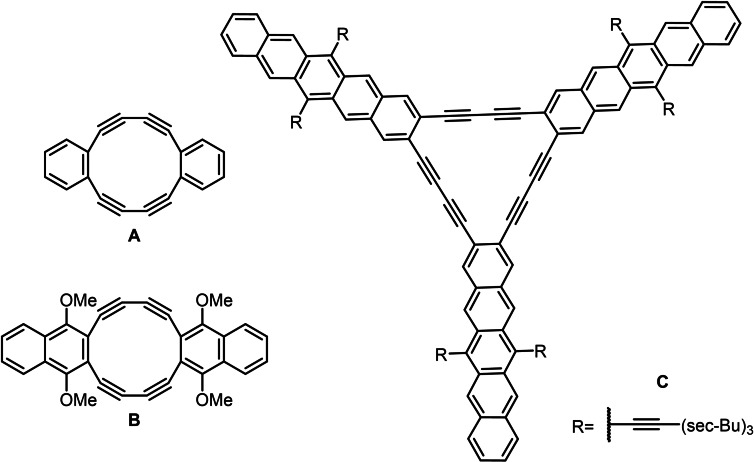
Structures of selected known arylene‐1,2‐bisalkynylene dimers and trimers.

We prepared the precursor 1,2‐diynes (Scheme 1) by Stille coupling of 1,2‐dibromoarenes using TMSCCSnMe_3_ and a bis(benzonitrile)palladium(II) chloride precatalyst^[9]^ in the presence of *t*Bu_3_P⋅HBF_4_; potassium carbonate liberates the terminal diynes, which were administered by a syringe pump into a solution of copper acetate and pyridine under air. Without a syringe pump, ill‐defined polymers formed‐we could not isolate or detect cyclooligomers, etc. by mass spectrometry. The purification of the target compounds, formed in 12–13 % for the cyclodimers and around 15–25 % for the cyclotrimers (Figure 2), was performed by gel permeation chromatography. Surprisingly, dimer **3** **b**_2_ could not be isolated, testament to its insolubility ‐ only **3** **b**_3_.

**Scheme 1:**
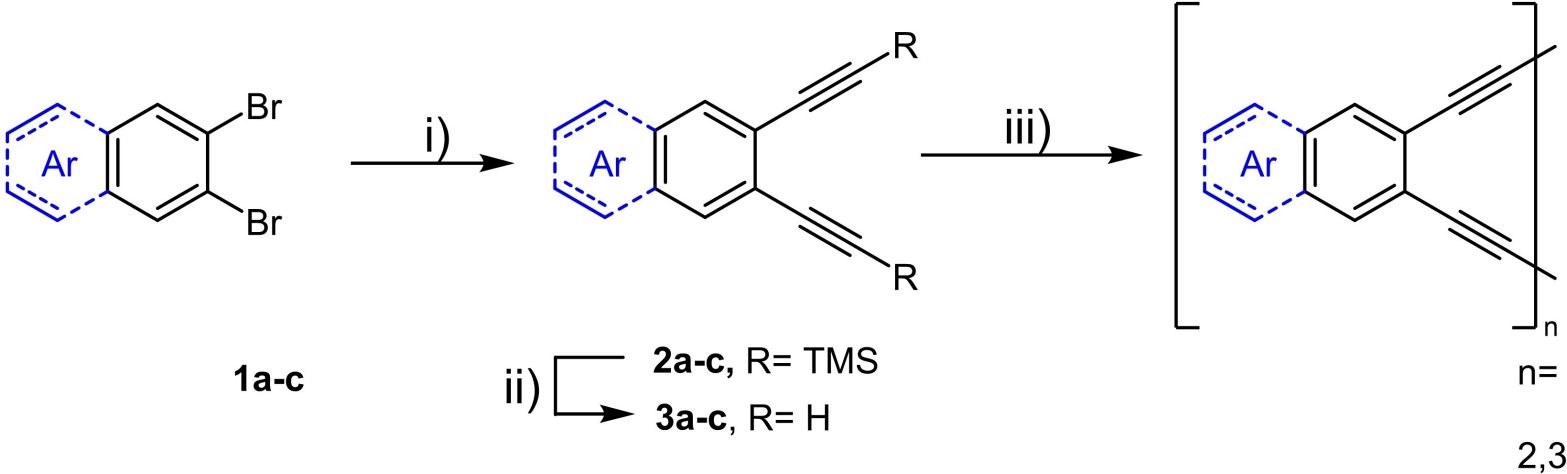
Synthetic route to (aza)acenylene‐alkynylene dimers and trimers. i) TMSCCSnMe_3_ (5.00 equiv.), (C_6_H_5_CN)_2_PdCl_2_ (10 mol%), P*t*Bu3HBF4 (20 mol%), THF, RT, 10 h; ii) K_2_CO_3_ (20.0 equiv.), THF/MeOH (1 : 1), RT, 1 h; iii) Cu(OAc)_2_ (22.0 equiv.), pyridine/MeOH (1 : 1), 60 °C, 24 h.

**Figure 2:**
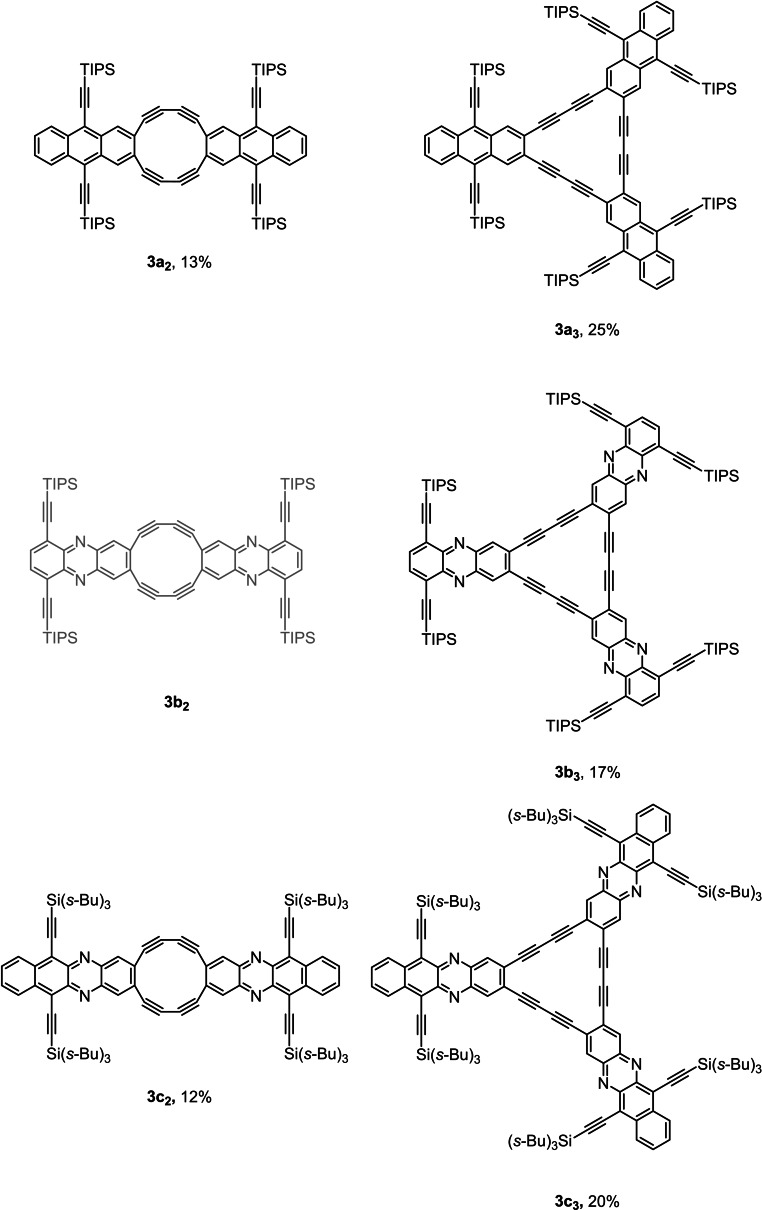
The prepared cyclooligomers including yields.

Attempts to cyclize diethynyltetraazapentacenes and their dihydro analogues failed, probably due to interfering redox processes. We noticed that the cyclodimers were always considerably less soluble than the cyclotrimers in variance to the observation made for the parent systems, and that, despite their decoration with R_3_Si‐ethynyl groups. To assess the thermal stability of the series, TGA/DSC experiments were conducted. All materials are stable up to 300 °C. The least stable compound is strained dimer **3** **c_2_**‐its decomposition begins at ∼300 °C. For the monomeric compound **3** **c** and the trimer **3** **c_3_**  decompostion approximately starts at 350 °C (Figures S46–S52 in the Supporting Information).

Figure 3 displays the UV‐vis spectra of monomeric **3** **c** and the cycles **3** **c_2,3_** . The cyclooligomerization leads to an increased red shift up to 31 nm for the absorption maximum. This is probably due to the diazatetracene‐dialkyne being the major chromophore; the conjugation through the diyne bridges is fairly weak, supported by the lack of ring current over the butadienylenes revealed by anisotropy of the induced current density (AICD) calculations^[10]^ (Figure S6). The same trend is visible for **3** **a** and **3** **a_2_**,_**3**_  and **3** **b** and **3** **b_3_**  (red shift in **3** **b**/**3** **b_3_** , only visible in the onset; Figures S31 and S32).

**Figure 3:**
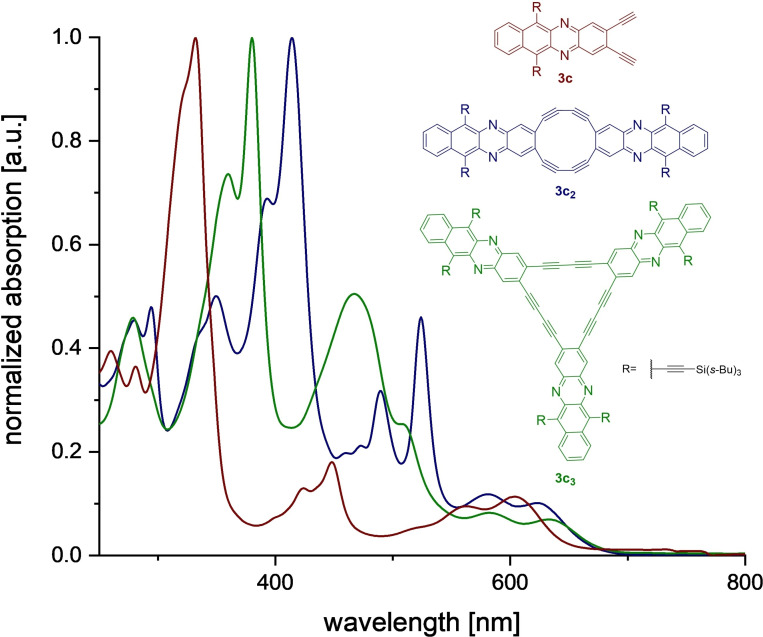
Normalized absorption spectra of diethynyldiazatetracene **3** **c**, its Glaser dimer **3** **c_2_** and trimer **3** **c_3_**  in CH_2_Cl_2_.

DFT calculations (B3LYP, def2‐TZVP) show that the frontier molecular orbitals are evenly distributed over the molecules. Tables 1 and 2 display the experimental and calculated optical and electronic values for the compounds reported herein. Cyclooligomerization stabilizes the LUMOs by up to 0.29 eV. This effect was not reproduced for the reduction events observed in the cyclovoltammograms, which do not differ significantly.

**Table 1:** Experimental optical properties of monomers **3** **a**–**c**, dimers **3** **a_2_**–**c_2_** and trimers **3** **a_3_‐c_3_**  in solution (CH_2_Cl_2_).

Compd	*λ*_max, abs_	*λ*_onset, abs_	*λ*_max, em_	Stokes shift	Quantum	Lifetime
	[nm]	[nm]	[nm]	[cm^−1^]	yield [%]^[a]^	[ns]
**3** **a**	458	472	472	648	79	7
**3** **a_2_**	472	489	536	2530	17	12
**3** **a_3_**	478	499	540	2402	18	11
**3** **b**	463	525	524	2515	<5[b]	–[b]
**3** **b_2_**	–	–	–		–	–
**3** **b_3_**	460	480	548	3490	<5	–[b]
**3** **c**	602	672	648	1180	<5	–[b]
**3** **c_2_**	622	672	678	1328	<5	–[b]
**3** **c_3_**	633	682	673	939	<5	–[b]

[a] A reference method using quinine sulfate was employed.^[13]^ [b] Emission was too weak for an exact measurement.

**Table 2:** Experimental and calculated (gas‐phase) electrochemical properties of monomers **3** **a**–**c**, dimers **3** **a_2_**–**c_2_** and trimers **3** **a_3_**_‐_ **c_3_**  in solution (CH_2_Cl_2_).

Compd	*E* ^(0/−)^ [V]^[a]^	Ionization potential [ev] ^[b]^	HOMO [eV]^[c]^	Electron affinity [eV]^[d]^	LUMO [eV]^[c]^
**3** **a**	−1.81	−5.92	−5.55	−3.29	−2.76
**3** **a_2_**	–[e]	–[e]	−5.41	−[e]	−2.99
**3** **a_3_**	−1.80	−5.78	−5.37	−3.30	−2.98
**3** **b**	−1.31		−6.07	−3.79	−3.23
**3** **b_2_**	–	–	−6.03	−	−3.52
**3** **b_3_**	−1.38		−6.00	−3.72	−3.50
**3** **c**	−1.11		−5.63	−3.99	−3.46
**3** **c_2_**	−1.02	−5.93	−5.60	−4.08	−3.72
**3** **c_3_**	−1.05	−5.87	−5.58	−4.05	−3.70

[a] First reduction potentials from cyclic voltammetry (CV) in CH_2_Cl_2_ at room temperature with Bu_4_NPF_6_ as the electrolyte against Fc/Fc^+^ as an internal standard (−5.10 eV)^[14]^ at 0.2 or 0.5 V/s. [b] Ionization potential=electron affinity−*λ*_onset,abs_. [c] Obtained from DFT calculations (Gaussian16,^15^B3LYP/def2‐TZVP). [d] Electron affinity=−e×(5.1 V+*E* ^(0/−)^). [e] Measurement not possible due to insufficient solubility.

We notice that the **c**‐series with diazatetracene as chromophore displays robust electron affinities of approximately −4.0 eV (measured) and −3.5 to −3.7 eV (calculated), thus suggesting that these should be electron transport materials in n‐channel transistors.

We obtained a single crystalline specimen of the target compounds (Figure 4 and the Supporting Information). The quasiplanar dimers **3** **a_2_** and **3** **c_2_** form slightly offset one‐dimensional staircases with π–π stacking distances of 351 and 338 pm (averaged through both chromophores and butadiinylene linkers), respectively. In the supramolecular structures, one acenylene subunit is centered above the octadehydro[12]annulene moiety of the neighboring molecule, hinting at less‐than‐ideal charge transport properties. Unlike the Hay‐type dimers, **3** **a**–**c_3_**  deviate from planarity ‐ the (aza)acenylene subunits are slightly twisted with respect to each other, a feature also observed in Anthony's pentacene‐based dehydroannulenes,[^11^, ^12^] which is most likely a crystal packing effect as gas‐phase structures were calculated to be planar. In contrast to the pentacene‐based annulene exhibiting three‐dimensional π‐stacking interactions, only two‐dimensional ones are observed in the trimers: In **3** **a_3_**  (co‐crystallyzing with chloroform) and **3** **c_3_**  (solvent‐free packing), two of the three “arms” overlap‐distances between π‐system distances amount to ∼3.4 Å. **3** **b_3_**  forms dimers in which two phenazine chromophores π‐stack (∼3.1  Å) on top of each other. These then π interact (∼4.7  Å with the neighboring dimer pairs forming a one‐dimensional slipped dimer stack.

**Figure 4:**
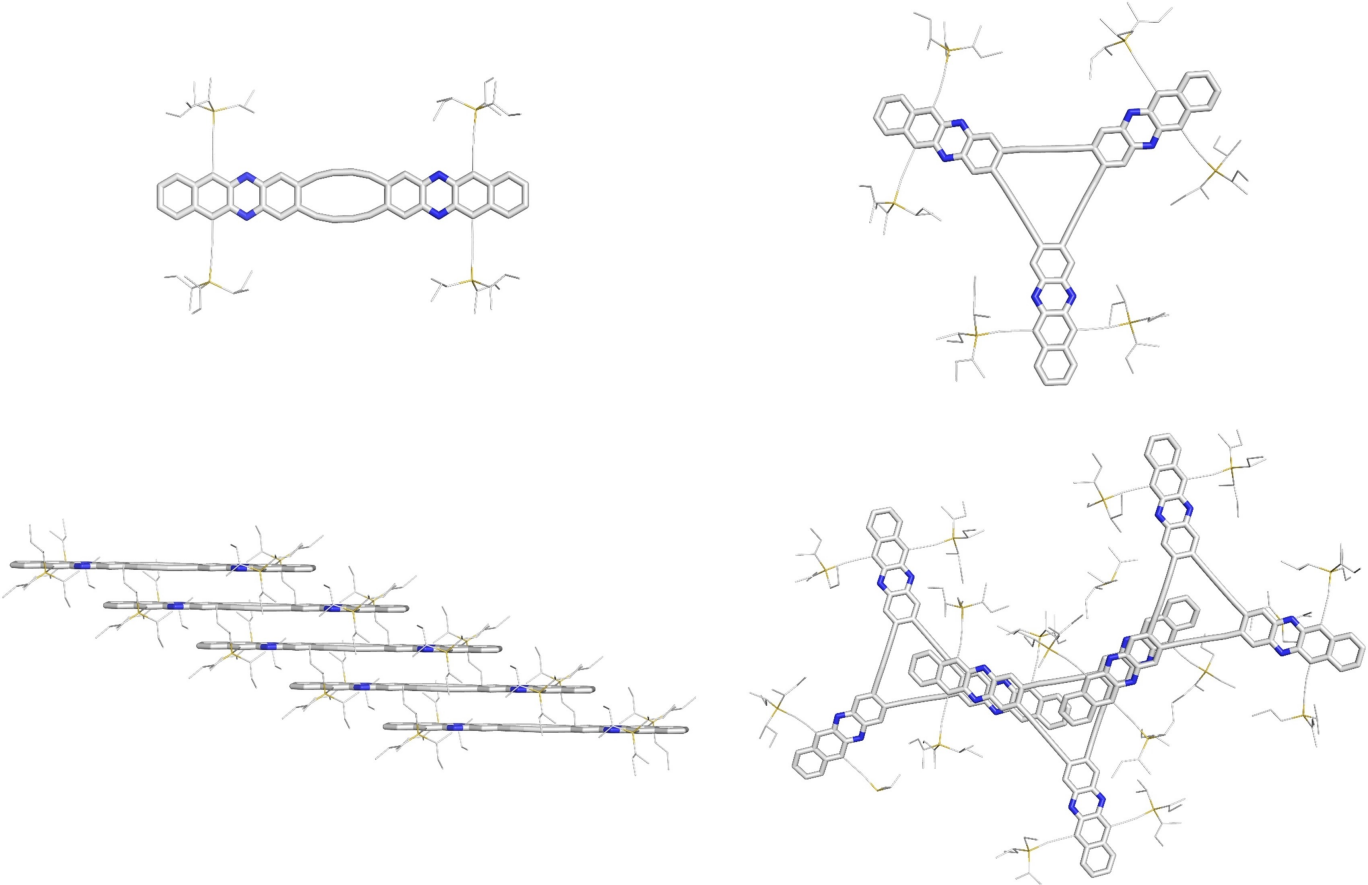
Single crystal structures and stacking of diazatetracene dimer **3** **c_2_** (left) and trimer **3** **c_3_**  (right). TIPS‐ethynyl substituents were reduced in size for visualization.

Due to their electrochemical properties and the possibility to obtain solvent‐free crystal structures, diazatetracene‐based **3** **c_2,3_**  were promising candidates for thin‐film transistors (TFTs). Note that only Anthony's trimer was employed in an organic electronic device, albeit in a photovoltaic cell.^[8]^ TFTs were fabricated in bottom gate/top contact architectures (Figure 5 and Supporting Information) with silver as contact electrodes, using a SAM modified dielectric.^[16]^ Thin films of **3** **c_2_** and **3** **c_3_**  were obtained by drop‐casting (**3** **c_2_**: toluene, 0.5 mg/mL; **3** **c_3_** : CH_2_Cl_2_, 0.5 mg/mL). The average electron mobility of the trimer (*μ*_ave_=1.85x10^−2^ cm^2^/V s) was one order of magnitude higher than that of the dimer (*μ*_ave_=2.10x10^−3^ cm^2^/V s), probably due to fewer grain boundaries formed upon evaporation of the volatile dichloromethane compared to the microcrystalline film of **3** **c_2_** from high‐boiling toluene solution (see Figure S43 for polarization micrographs), and as a consequence of the higher transfer integrals (38.8 meV for **3** **c_3_**  and 3.55 meV for **3** **c_2_**) for electron transport (Figure S7). Although TIPS‐ethynylated diazatetracene,^[17]^ which was found to pack in a brick‐wall motif, exhibited an electron mobility of *μ*_lit_ = 5x10^−2^ cm^2^/V s,^[18]^ the mobilities of trimer **3** **c_3_**  are on the same order of magnitude and that despite bulkier (*s*Bu)_3_Si‐ethynyl groups being used to solubilize the material.

**Figure 5:**
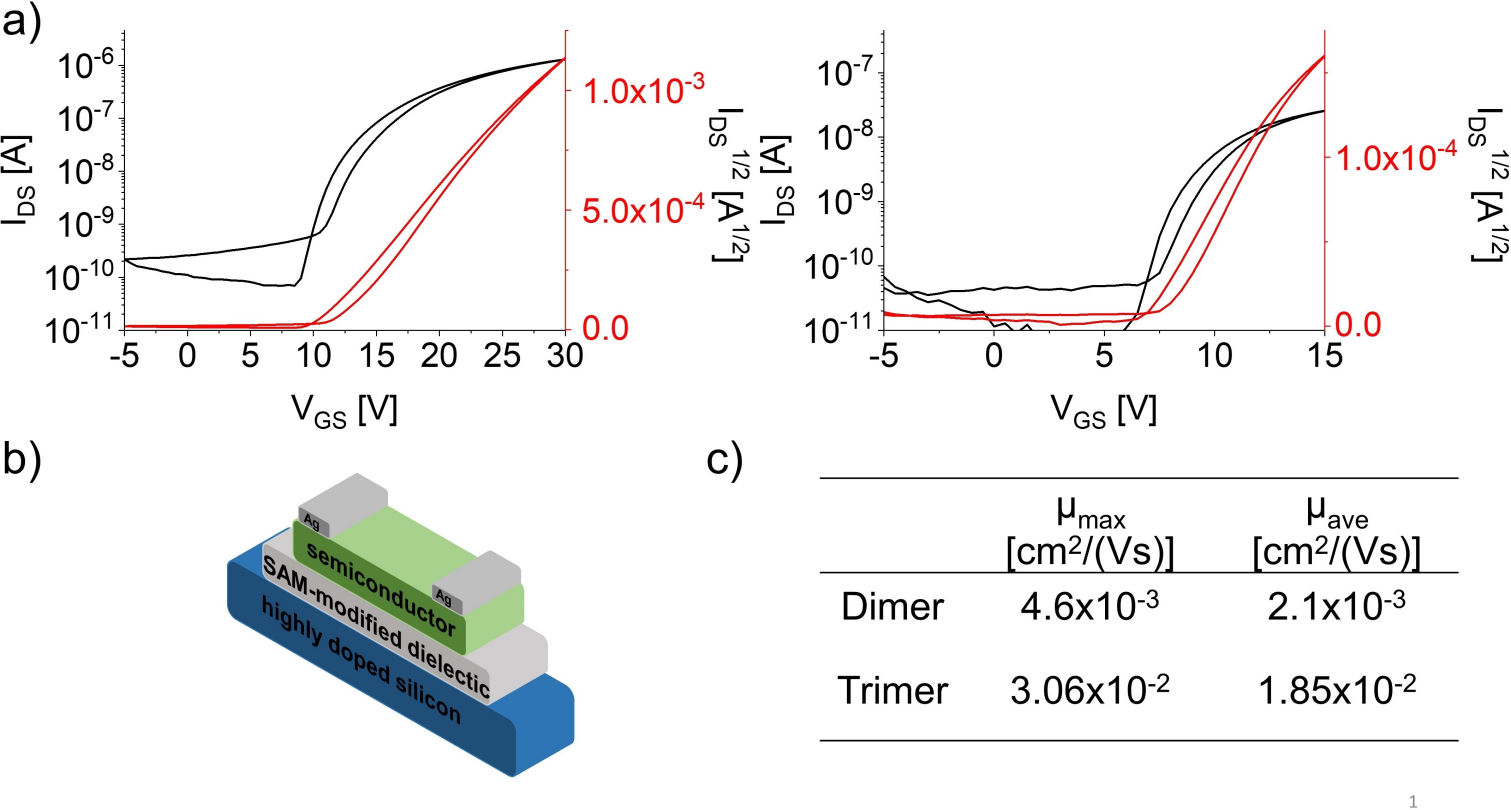
a) Transfer characteristics of trimer **3** **c_3_**  (left; *V*_DS_=35 V) and dimer **3** **c_2_** (right; *V*_DS_=20 V). b) Schematic bottom‐gate/top‐contact device architecture. c) Charge transfer mobilities of dimer **3** **c_2_** and trimer **3** **c_3_** .

In conclusion, we have synthesized and characterized novel acene‐based cyclodimers **3** **a_2_** and 3**c_2_** as well as cyclotrimers **3** **a_3_**–**c_3_** . Cyclodimer **3** **c_2_** and cyclotrimer **3** **c_3_**  were used as organic semiconductors in bottom gate/top contact thin‐film transistors. The mobility of the trimer is an order of magnitude higher than that of the cyclodimer, although the electronic and optical properties are quite similar ‐ most likely resulting from improved π–π interactions between the azaacene subunits. The cyclotrimer3c_3_ shows mobilities in the same order as that of unsubstituted tetraazatetracene in the literature, although *sec*‐butyl‐silyl groups were used. This highlights the importance of the solid‐state architecture, which could be influenced by modifying the structure of the single molecule.

## Experimental Section

**Representative procedure towards Glaser–Hay dimers 3** **c_2_ and 3** **c_3_** : Under ambient atmosphere, copper(II) acetate (3.04 g, 15.2 mmol, 22.0 equiv.) was dissolved in a mixture of 175 mL pyridine and 175 mL methanol. The mixture was heated to 60 °C. Afterwards a solution of **3** **c** (500 mg, 691 μmol, 1.00 equiv.) in 20 mL of pyridine was added dropwise using a syringe pump (rate 0.06 mmol/h). After completion of the addition, the reaction mixture was stirred for additional 4 h. After cooling to room temperature the solvent was removed under reduced pressure. Water and CH_2_Cl_2_ were added and the mixture was extracted with CH_2_Cl_2_. The combined organic layers were died over MgSO_4_ and the solvent was removed under reduced pressure. The crude product was purified by column chromatography on silica (PE/CH_2_Cl_2_ 8 : 2, 7 : 3, 1 : 1) to yield a mixture of **3** **c_2_** and **3** **c_3_**  containing some small impurities. Purification of the mixture using a GPC yielded the dimer **3** **c**_2_ (60.0 mg, 12 %) as a dark brown solid and the trimer **3** **c_3_**  (100 mg, 20 %) as a dark brown solid.

Deposition Numbers 2106563 (for **3** **a**_**2**_), 2106564 (for **3** **a**_**3**_ ), 2106565 (for **3** **b**_**3**_ ), 2106566 (for **3** **c**_**2**_), and 2106567(for **3** **c**_**3**_ ) contain the supplementary crystallographic data for this paper. These data are provided free of charge by the joint Cambridge Crystallographic Data Centre and Fachinformationszentrum Karlsruhe Access Structures service.

Data related to this article are available through heiDATA, the institutional research data repository of Heidelberg University, under https://doi.org/10.11588/data/24JDZD.

## Conflict of interest

The authors declare no conflict of interest.

## Supporting information

As a service to our authors and readers, this journal provides supporting information supplied by the authors. Such materials are peer reviewed and may be re‐organized for online delivery, but are not copy‐edited or typeset. Technical support issues arising from supporting information (other than missing files) should be addressed to the authors.

Supporting Information
