# Supplementary material for: Cyclodimers and Cyclotrimers of 2,3 ‐Bisalkynylated Anthracenes, Phenazines and Diazatetracenes
Source: Chemistry. 2021 Nov 5;27(66):16320–4. doi: 10.1002/chem.202103193 (PMC9297893; doi:10.1002/chem.202103193)
Supplement: Supplementary file 1 — Supporting Information [file CHEM-27-16320-s001.pdf]

Chemistry–A European Journal

Supporting Information

Cyclodimers and Cyclotrimers of 2,3-Bisalkynylated Anthracenes, Phenazines and Diazatetracenes

Steffen Maier, Nikolai Hippchen, Frank Rominger, Jan Freudenberg,* and Uwe H. F. Bunz*

SUPPORTING INFORMATION

Table of Contents

1 Experimental Procedures........ 2
1.1 Materials and Methods........ 2
1.2 Synthesis........ 2
2. Results and Discussion........ 11
2.1 Calculations........ 11
2.1.1 FMO and AICD calculation........ 11
2.1.2 Calculation of Transfer Integrals........ 14
2.1.3 Coordinates of the Optimized Geometries........ 15
2.2 NMR Spectroscopy........ 35
2.3. UV-vis spectra........ 48
2.4 Cyclic Voltammetry........ 49
2.5 Hirshfeld Anaylsis........ 52
2.6 TGA/DSC Measurements........ 54
2.7 OFET Fabrication and Measurement........ 57
2.6 Crystallographic Data........ 58
References........ 65

1 Experimental Procedures

1.1 Materials and Methods

Column chromatography was performed using silica gel from Sigma Aldrich. (particle size: 0.032−0.062 mm). Preparative GPC was performed on Bio-Beads® (S-X1 Beads, 200 – 400 Mesh, crosslinked polystyrene), purchased from Bio-Rad Laboratories, Inc., using toluene as solvent. NMR spectra were recorded on Bruker Avance Spectrometers using the specified frequency. Chemical shifts (δ) are given in parts per million (ppm) relative to internal solvent signals.[S1] The following abbreviations describe the signal multiplicities: s = singlet, d = doublet, dd = doublet of doublets, m = multiplet. High-resolution mass spectra (HRMS) were obtained by (matrix-assisted) laser desorption/ionization (LDI/MALDI) using *trans*-2-[3-(4-*tert*-butylphenyl)-2-methyl-2-propenylidene]malononitrile (DCTB) as matrix, electrospray ionisation (ESI) or direct analysis in real time (DART) experiments. IR spectra were recorded as a neat oil or powder of the respective analyte on a Jasco FT/IR-4100 spectrometer. CV measurements were performed on a Metrohm Autolab PGSTAT101. Absorption spectra were recorded on a Jasco UV-Vis V-660 or Jasco UV-Vis V 670. Fluorescence spectra were recorded on a Jasco FP-6500. Quantum lifetimes (τ_s) were determined on a Horiba FluoroCube-01-NL lifetime spectrofluorometer with emission monochromator (Seya-Namioka type, 200 nm to 800 nm) and diode excitation (Nano-LED N-375L, 375 ± 10 nm, < 200 ps) All calculations were performed using Gaussian16. TMS groups were used instead of TIPS groups to simplify calculations. First, the gas ‐ phase ground ‐ state equilibrium geometry of the molecules was optimized at the B3LYP/def2 ‐ SVP level of theory. Afterwards, the recieved geometries were refined using the B3LYP/def2-TZVP level of theory. FMO calculations were performed starting from the optimized geometries on the B3LYP/def2-TZVP level of theory.[S2] ACID-plots were calculated starting from the optimized geometries using AICD-3.0.3 using the CSGT-method at the B3LYP/def2-TZVP IOP(10/93=1) level of theory; isovalue: 0.02; optimization limit: 0.02; maximal arrow length: 1; magnetic field vector is oriented out of plane.[S3] TIPS-Dibromoanthracene **1a** was synthesized according to a literature procedure.[S4] Hirshfeld surfaces and fingerprint plots were generated with the program CrystalExplorer 17.5.[S5]. P-doped Si-wafers with 100 nm thermally grown silicon dioxide were purchased from Si-Mat. Metals used for device contacts were produced by Kurt J. Lesker Company (East Sussex, United Kingdom) and thermally evaporated. Pictures were taken at ambient conditions on a Nikon Eclipse LV100POL microscope with the included camera. TGA/DSC measurements were carried out on a Mettler-Toledo TGA/DSC1 instrument with a TGA/DSC-Sensor 1100 equipped with a MX1 balance (Mettler-Toledo) and a GC100 gas control box for nitrogen supply.

1.2 Synthesis

((9,10-Bis((triisopropylsilyl)ethynyl)anthracene-2,3-diyl)bis(ethyne-2,1-diyl))bis(trimethylsilane) (2a)

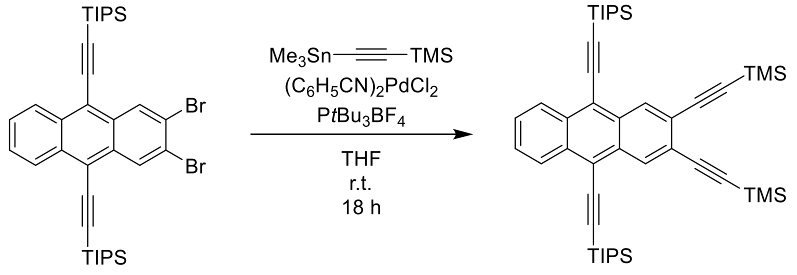

In a flame dried Schlenk tube **1a** (3.00 g, 4.31 mmol, 1.00 eq) was dissolved in 200 mL anhydrous THF under nitrogen atmosphere. The solution was degassed for 30 min. Trimethyl((trimethylstannyl)ethynyl)silane (5.62 g, 21.5 mmol, 5.00 eq.) was added and the resulting solution was degassed for additional 20 min. Subsequently, bis(benzonitrile)palladium (II) chloride (82.6 mg, 215 µmol, 5.00 mol%) and tri-*tert* butylphosphine tetrafluoroborate (125 mg, 430 µmol, 10.0 mol%) were added. The reaction mixture was stirred for 12 h. The solvent was removed under reduced pressure and the crude product was purified by column chromatography on silica to yield the product (3.08 g, 4.22 mmol, 98%) as a yellow solid.

^{1}H NMR (600 MHz, 295 K, $CDCl_3$) δ= 8.80 (s, 2H), 8.56-8.54 (m, 2H), 7.60-7.59 (m, 2H), 1.27-1.26 (m, 42 H), 0.31 (s, 18 H) ppm.

$^{13}C\{^{1}H\}$ NMR (151 MHz, 295 K, $CDCl_3$) δ= 133.0, 132.6, 131.2, 127.6, 127.5, 122.7, 118.6, 106.1, 103.5, 102.8, 99.0, 19.0, 11.7, 0.1 ppm.

ATR-IR: $\tilde{\nu}$ [cm^{-1}] = 3628, 2941, 2889, 2863, 2149, 1460, 1447, 1379, 1366, 1248, 1211, 1156, 1038, 1016, 997, 932, 898, 787, 690, 676, 658, 635, 612, 591, 556, 503, 467, 445, 440, 435, 401.

HR-MS (MALDI pos.) m/z: $[M]^+$: calcd. for $[C_{46}H_{66}Si_4]^+$: 730.4242; found: 730.4245.

Melting point: > 300 °C.

R_f (PE) = 0.2

((2,3-Diethynylanthracene-9,10-diyl)bis(ethyne-2,1-diyl))bis(triisopropylsilane) (3a)

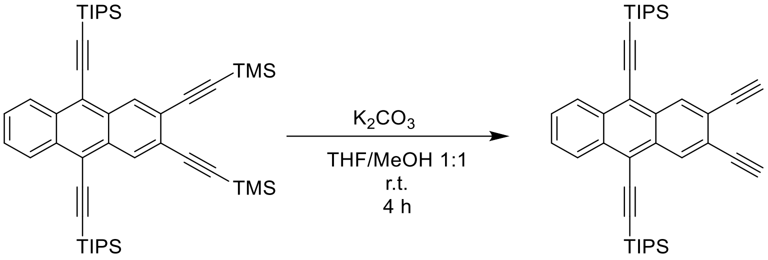

2a **3a**

2a (1.00 g, 137 µmol, 1.00 eq.) was dissolved in a mixture of 30 mL THF and 30 mL MeOH. Subsequently K_2CO_3 (95.0 mg, 684 µmol, 5.00 eq.) was added and the reaction mixture was stirred for 4 h at room temperature. Water was added and the mixture was extracted with DCM. The combined organic layers were dried over $MgSO_4$ to yield the product (786 mg, 134 µmol, 98%) as a yellow solid.

^{1}H NMR (600 MHz, 295 K, $CDCl_3$) δ= 8.85 (s, 2H), 8.59-8.58 (m, 2H), 7.63-7.62 (m, 2H), 1.27-1.26 (m, 42 H), ppm.

$^{13}C\{^{1}H\}$ NMR (151 MHz, 295 K $CDCl_3$) δ= 133.2, 133.1, 131.1, 127.8, 127.6, 121.7, 118.8, 106.4, 102.6, 82.2, 81.4, 19.0, 11.6 ppm.

ATR-IR: $\tilde{\nu}$ [cm-1] = 2939, 2924, 2885, 2861, 2364, 2358, 2341, 2336, 2330, 1557, 1495, 1480, 1464, 1456, 1431, 1382, 1285, 1196, 1187, 1105, 1053, 1020, 992, 880, 845, 771, 746, 732, 697, 683, 670, 663, 646, 639, 624, 619, 608, 597, 589, 576, 510, 504, 500, 487, 481, 463, 456.

HR-MS (MALDI pos.) m/z: $[M+H]^+$: calcd. for $[C_{40}H_{50}Si_2]^+$: 586.3446; found: 586.3448.

Melting point: > 300 °C.

R_f (PE) = 0.2

[{24-[4-Methyl-3,3-di(propan-2-yl)pent-1-yn-1-yl]-7,8,9,10,19,20,21,22-octadehydrocyclododeca[1,12-*b*:6,7-*b'*]dianthracene-5,12,17-triyl}tri(ethyne-2,1-diyl)]tris[tri(propan-2-yl)silane] ($3a_2$)/ [{36-[4-Methyl-3,3-di(propan-2-yl)pent-1-yn-1-yl]-7,8,9,10,19,20,21,22,31,32,33,34-dodecadehydrocyclooctadeca[1,18-*b*:12,13-*b'*:6,7-*b''*]trianthracene-5,12,17,24,29-pentayl}penta(ethyne-2,1-diyl)]pentakis[tri(propan-2-yl)silane]($3a_3$)

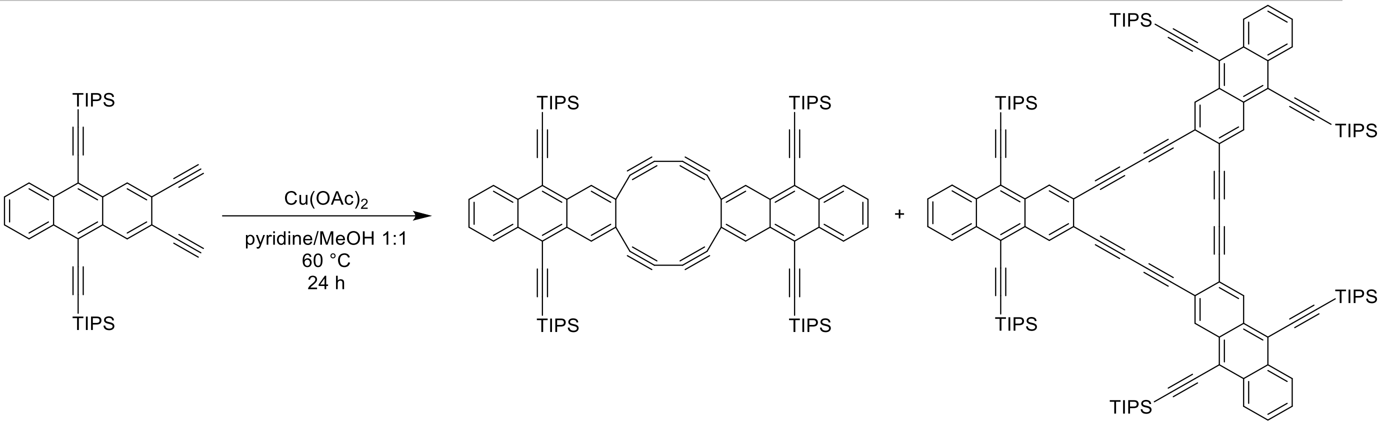

3a 3a$_2$ 3a$_3$

Copper (II) acetate (6.00 g, 30.0 mmol, 22.0 eq.) was dissolved in a mixture of 400 mL pyridine and 400 mL methanol. The mixture was heated to 60 °C. A solution of **3a** (800 mg, 1.36 mmol, 1.00 eq.) in 20 mL of pyridine was added dropwise using a syringe pump (rate 0.06 mmol/h). After completion of the addition, the reaction mixture was stirred for additional 4 h. After cooling to room temperature the solvent was removed under reduced pressure. Water and DCM were added and the mixture was extracted with DCM. The combined organic layers were died over $MgSO_4$ and the solvent was removed under reduced pressure. The crude product was purified by column chromatography on Silica (PE:DCM 9:1, 8:2, 1:1) to yield a mixture of **3a$_2$** and **3a$_3$** containing some small impurities. Purification of the mixture using GPC yielded the dimer **3a**$_2$ (104 mg, 89.0 µmol 13%) as an orange brown solid and the trimer **3a$_3$** (200 mg, 171 µmol, 25%) as an orange brown solid.
.

3a$_2$:

^{1}H NMR (600 MHz, 295 K, THF-d$_8$) δ= 8.62-8.60 (m, 8H), 7.72-7.70 (m, 4H), 1.32-1.31 (m, 84 H), ppm.

^{13}C{^{1}H} NMR (151 MHz, 295 K, THF-d$_8$) δ= 134.5, 132.2, 129.6, 129.3, 128.3, 125.7, 120.6, 107.8, 103.3, 93.0, 85.5, 19.4, 12.6 ppm.

ATR-IR: $\tilde{\nu}$ [cm^{-1}] = 2941, 2923, 2863, 2725, 2361, 2144, 2138, 2128, 1734, 1619, 1457, 1377, 1337, 1290, 1241, 1156, 1071, 1047, 1015, 996, 919, 892, 883, 857, 817, 762, 701, 605, 592, 569, 565, 503, 479, 466, 455, 449, 436, 424, 414, 401.

HR-MS (MALDI pos.) m/z: [M+H]$^+$: calcd. for $[C_{80}H_{97}Si_4]^+$: 1169.6662; found: 1169.6684.

Melting point: > 300 °C.

R$_f$ (PE:DCM 9:1) = 0.4

3a$_3$:

^{1}H NMR (300 MHz, 295 K, CDCl$_3$) δ= 8.94 (s, 2H), 8.65-8.62 (m, 2H), 7.67-7.64 (m, 2H), 1.35-1.33 (m, 42 H), ppm.

^{13}C{^{1}H} NMR (126 MHz, 295 K CD$_2$Cl$_2$) δ= 134.2, 133.8, 131.3, 129.4, 128.5, 127.9, 125.6, 121.6, 119.4, 107.5, 102.6, 81.7, 78.6, 19.1, 11.9 ppm.

ATR-IR: $\tilde{\nu}$ [cm^{-1}] = 3839, 3735, 3650, 2941, 2889, 2863, 2362, 2357, 2344, 2140, 2135, 2123, 1734, 1717, 1684, 1559, 1507, 1458, 1380, 1366, 1340, 1275, 1267, 1249, 1184, 1157, 1136, 1072, 1048, 1016, 993, 918, 894, 880, 828, 758, 735, 726, 710, 689, 675, 655, 603, 591, 564, 559, 503, 486, 472, 462, 458, 447, 436, 429, 424, 419, 410, 406, 402.

HR-MS (MALDI pos.) m/z: [M]$^+$: calcd. for $[C_{120}H_{144}Si_6]^+$: 1752.9878; found: 1752.9889.

Melting point: > 300 °C.

R$_f$ (PE:DCM 9:1) = 0.6

7,8-Dibromo-1,4-bis((triisopropylsilyl)ethynyl)phenazine (1b)

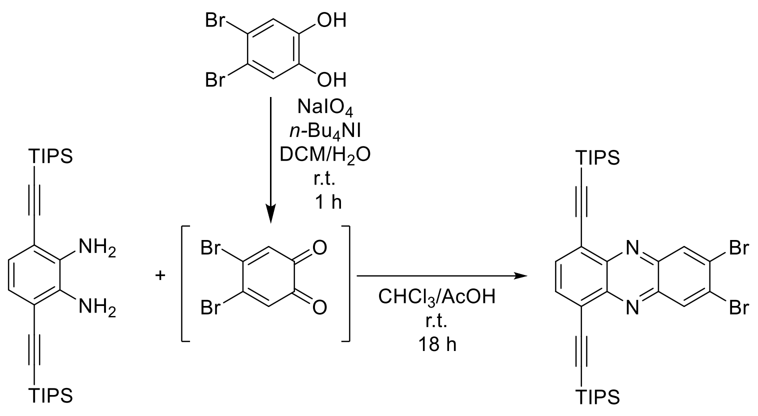

S3 **1b**

Diol **S2** (180 mg, 672 µmol, 1.00eq.) was dissolved in 5 mL of DCM. Subsequently sodium (meta)periodate (172 mg, 806 µmol, 1.20 eq.) and NBu_4I (10.0 mg, 26.9 µmol, 0.04 eq.) were added. After addition of 5 mL of water, the reaction mixture was stirred for 1 h at room temperature. The reaction mixture was extracted with DCM and the combined organic layers were dried over $MgSO_4$. After removing of the solvent, crude quinone was dissolved in a mixture of 2 mL $CHCl_3$ and 2 mL acetic acid. After addition of diamine **S3** (200 mg, 427 µmol, 0.66 eq.) the reaction mixture was stirred at room temperature overnight. The end of the reaction was monitored by TLC and a saturated aqueous solution of $NaHCO_3$ was added to neutralize the reaction mixture. The aqueous phase was extracted with DCM, the combined organic layers were dried over $MgSO_4$ and the solvent was removed under reduced pressure. The crude product was purified by column chromatography on silica gel (PE:DCM 95:5, 9:1) to yield **1b** as an orange solid (58.0 mg, 83.3 µmol, 19%).

^{1}H NMR (400 MHz, 295 K, $CDCl_3$) δ= 8.55 (s, 2H), 7.95 (s, 2H), 1.25-1.24 (m, 42 H), ppm.

^{13}C{^{1}H} NMR (101 MHz, 295 K $CDCl_3$) δ= 144.0, 142.5, 134.7, 133.9, 128.2, 124.7, 103.2, 101.7, 18.9, 11.6 ppm.

ATR-IR: $\tilde{\nu}$ [cm^{-1}] = 2955, 2941, 2890, 2863, 1463, 1446, 1418, 1406, 1367, 1244, 1129, 1073, 1050, 1040, 1035, 996, 879, 849, 791, 675, 663, 642, 628, 593, 585, 665, 659, 649, 640, 628, 593, 589, 585, 577, 566, 559, 549, 541, 534, 525, 521, 516, 509, 504, 494, 487, 481, 476, 472, 468, 463, 456, 449, 444, 439, 436, 430, 420, 417, 412, 407.

HR-MS (MALDI pos.) m/z: [M]$^+$: calcd. for $[C_{34}H_{46}N_2Si_2{}^{79}Br^{81}Br]^+$: 698.1551; found: 698.1551.

Melting point: > 300 °C.

R$_f$ (PE:DCM 9:1) = 0.3

1,4-Bis((triisopropylsilyl)ethynyl)-7,8-bis((trimethylsilyl)ethynyl)phenazine (2b)

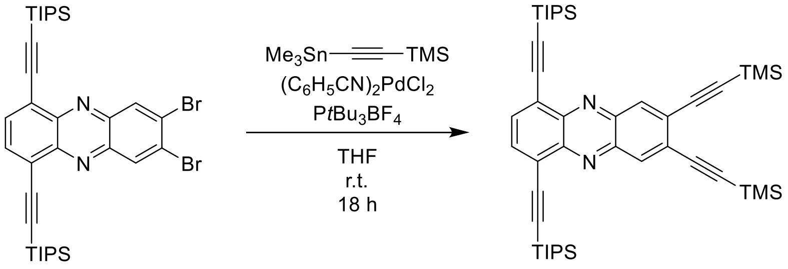

1b **2b**

In a flame dried Schlenk tube **1b** (450 mg, 644 µmol, 1.00 eq) was dissolved in 50 mL dry THF under nitrogen atmosphere. The solution was degassed for 30 min. Trimethyl((trimethylstannyl)ethynyl)silane (841 g, 3.22 mmol, 5.00 eq.) was added and the resulting solution was degassed for additional 20 min. Subsequently bis(benzonitril) palladium (II) chloride (24.7 mg, 64.4 µmol, 10.0 mol%) and tri-*tert* butylphosphine tetrafluoroborate (37.4 mg, 129 µmol, 20.0 mol%) were added. The reaction mixture was stirred for 12 h. The solvent was removed under reduced pressure and the crude product was purified by column chromatography on silica gel (PE:DCM 9:1→ 8:2) to yield the product (425 mg, 580 µmol, 90%) as an orange solid.

^{1}H NMR (400 MHz, 295 K, $CDCl_3$) δ= 8.30 (s, 2Hs), 7.92 (s, 2Hs), 7.60-7.59 (m, 2Hs), 1.25 (m, 42 Hs), 0.35 (s, 18 Hs) ppm.

^{13}C{^{1}H} NMR (101 MHz, 295 K, $CDCl_3$) δ= 144.0, 142.8, 134.4, 134.3, 127.3, 124.7, 103.5, 102.6, 102.4, 101.2, 19.0, 11.6, 0.1 ppm.

ATR-IR: $\tilde{\nu}$ [cm^{-1}] = 2942, 2891, 2864, 2155, 1457, 1443, 1419, 1412, 1383, 1366, 1350, 1249, 1229, 1186, 1145, 1126, 1071, 1048, 1037, 1013, 996, 990, 878, 841, 814, 807, 784, 760, 701, 675, 660, 643, 636, 607, 592, 553, 549, 520, 503, 486, 476, 470, 461, 455, 425, 421.

HR-MS (MALDI pos.) m/z: [M+H]$^+$: calcd. for [$C_{44}H_{65}N_2Si_4$]$^+$: 733.4219; found: 733.4208.

Melting point: > 300 °C.

R$_f$ (PE:DCM 9:1) = 0.2

7,8-Diethynyl-1,4-bis((triisopropylsilyl)ethynyl)phenazine (3b)

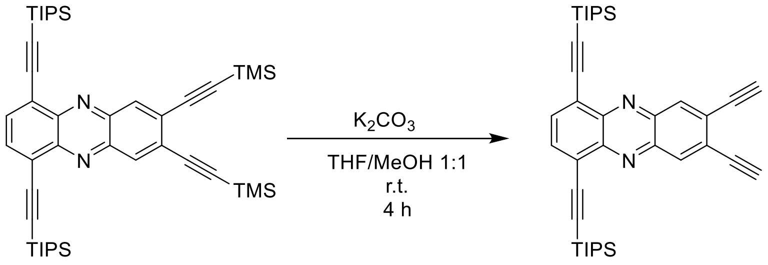

2b **3b**

2b (295 mg, 402 µmol, 1.00 eq.) was dissolved in a mixture of 10 mL THF and 10 mL MeOH. Subsequently K_2CO_3 (278 mg, 2.01 mmol, 5.00 eq.) was added and the reaction mixture was stirred for 4 h. Water was added and the mixture was extracted with DCM. The combined organic layers were dried over $MgSO_4$. The crude product was purified by column chromatography on silica gel (PE:DCM 8:2) to yield the product (227 mg, 386 µmol, 96%) as an orange solid.

^{1}H NMR (400 MHz, 295 K, CD_2Cl_2) δ= 8.41 (s, 2H), 7.98 (s, 2H), 3.65 (s, 2H), 1.26 (m, 42 H), ppm.

^{13}C{^{1}H} NMR (101 MHz, 295 K CD_2Cl_2) δ= 144.5, 143.0, 135.1, 135.0, 126.6, 125.0, 103.7, 101.7, 84.6, 81.4, 19.0, 11.9 ppm.

ATR-IR: $\tilde{\nu}$ [cm^{-1}] = 3308, 2942, 2890, 2360, 2356, 2342, 2336, 2312, 2163, 2147, 2026, 2002, 1968, 1653, 1559, 1507, 1457, 1247, 1124, 1072, 1051, 1037, 1017, 997, 891, 882, 850, 798, 675, 669, 662, 658, 654, 649, 644, 635, 631, 626, 621, 618, 609, 602, 598, 593, 585, 567, 559, 556, 546, 538, 533, 524, 519, 507, 499, 492, 485, 481, 472, 463, 453, 448, 442, 434, 429, 426, 412, 406.

HR-MS (MALDI pos.) m/z: [M]$^+$: calcd. for [$C_{38}H_{49}N_2Si_2$]$^+$: 589.3429; found: 589.3426.

Melting point: > 300 °C.

R$_f$ (PE:DCM 8:2) = 0.3

5,8-Bis{[tri(propan-2-yl)silyl]ethynyl}quinoxaline—1-[4-methyl-3,3-di(propan-2-yl)pent-1-yn-1-yl]-4,21,24-tris{[tri(propan-2-yl)silyl]ethynyl}-7,8,9,10,15,16,17,18,27,28,29,30-dodecadehydroanthra[2',3':13,14]benzo[7,8]cyclooctadeca[1,2-*b*]phenazine (3b$_3$)

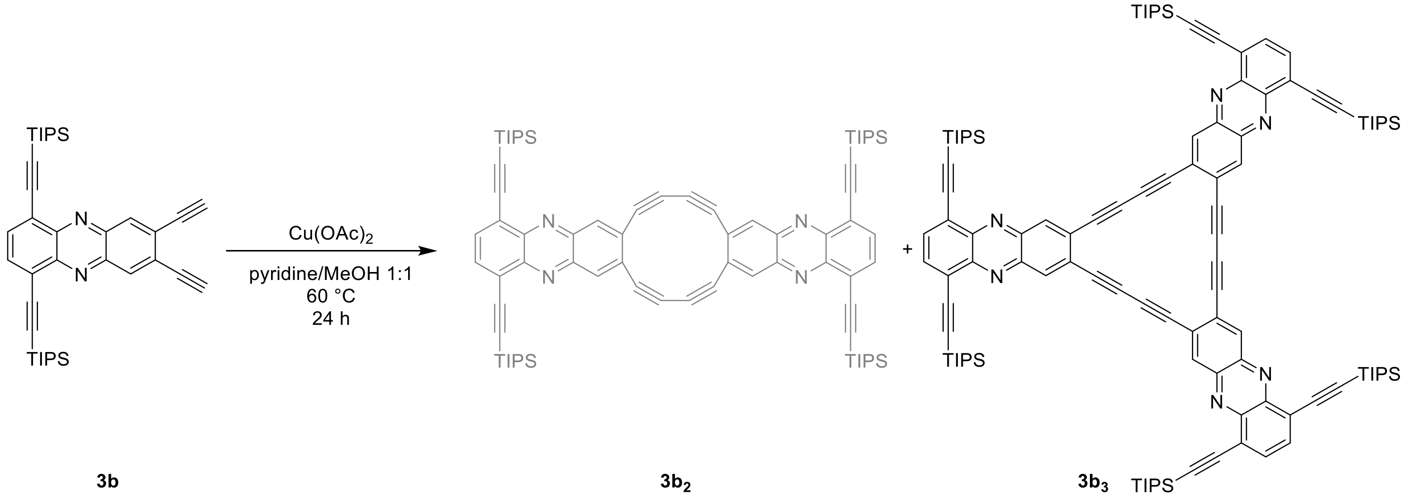

3b **3b$_2$** **3b$_3$**

Copper (II) acetate (969 mg, 4.88 mmol, 22.0 eq.) was dissolved in a mixture of 70 mL pyridine and 70 mL methanol. The mixture was heated to 60 °C. Afterwards a solution of **3b** (130 mg, 221 µ↕mol, 1.00 eq.) in 20 mL of pyridine was added dropwise using a syringe pump (rate 0.06 mmol/h). After completion of the addition, the reaction mixture was stirred for additional 4 h. After cooling to room temperature the solvent was removed under reduced pressure. Water and DCM were added and the mixture was extracted with DCM. The combined organic layers were died over $MgSO_4$ and the solvent was removed under reduced pressure. The crude product was purified by column chromatography on silica (PE:DCM 9:1, 8:2, 1:1) to yield a mixture of **3b$_3$** containing some small impurities. Purification of the mixture using a GPC yield the trimer **3b$_3$** (22 mg, 12.5 µmol, 17%) as an orange brown solid. **3b$_2$** was not isolated, probably due to its low solubility.

.

3b$_3$:

^{1}H NMR (600 MHz, 295 K, CDCl$_3$) δ= 8.51 (s, 2Hs), 7.98 (s, 2Hs), 1.29 (m, 42 Hs), ppm.

^{13}C{^{1}H} NMR (151 MHz, 295 K CDCl$_3$) δ= 144.3, 142.8, 136.0, 135.0, 125.6, 124.7, 103.2, 101.8, 81.6, 80.8, 19.0, 11.6 ppm.

ATR-IR: $\tilde{\nu}$ [cm^{-1}] = 3670, 2941, 2889, 2863, 2776, 2756, 2723, 2365, 2361, 2341, 2337, 2331, 2235, 2211, 2205, 2202, 2196, 2184, 2178, 2170, 2167, 2161, 2153, 2146, 2140, 2135, 2128, 2117, 2113, 2106, 2101, 2092, 2078, 2075, 2070, 2065, 2040, 2036, 2031, 2022, 2015, 1991, 1986, 1973, 1884, 1605, 1567, 1500, 1462, 1456, 1441, 1395, 1383, 1366, 1337, 1301, 1245, 1221, 1159, 1135, 1123, 1073, 1050, 1036, 1017, 995, 919, 908, 882, 848, 791, 758, 743, 734, 722, 697, 674, 659, 641, 600, 591, 587, 564, 545, 538, 534, 525, 506, 503, 494, 487, 477, 471, 466, 462, 454, 450, 447, 440, 432.

HR-MS (MALDI pos.) m/z: $[M]^+$: calcd. for $[C_{114}H_{138}N_6Si_6]^+$: 1758.9599; found: 1758.9830.

Melting point: > 300 °C.

$\mathbf{R_f}$ (PE:DCM 8:2) = 0.3

2,3-Dibromo-6,11-bis((*sec*-butyl-l2-silyl)ethynyl)benzo[*b*]phenazine--3,4-dimethylhexane (1c)

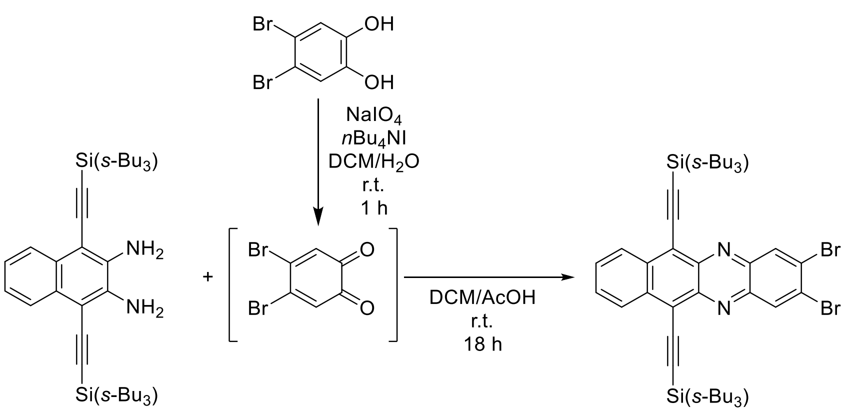

S4 1c

Diol **S2** (1.54 g, 5.75 µmol, 1.00eq.) was dissolved in 30 mL of DCM. Subsequently sodium meta periodate (1.48 g, 6.90 mmol, 1.20 eq.) and NBu_4I (70.9 mg, 230 µmol, 0.04 eq.) were added. After addition of 30 mL of water, the reaction mixture was stirred for 1 h at room temperature. The reaction mixture was extracted with DCM and the combined organic layers were dried over $MgSO_4$. After removing of the solvent, crude quinone was dissolved in a mixture of 10 mL DCM and 10 mL acetic acid. After addition of diamine **S4** (1.50 g, 2.49 mmol, 0.43 eq.) the reaction mixture was stirred at room temperature over night. The end of the reaction was monitored by TLC and an aqueous solution of $NaHCO_3$ was added to neutralize the reaction mixture. The aqueous phase was extracted with DCM, the combined organic layers were dried over $MgSO_4$ and the solvent was removed under reduced pressure. The crude product was purified by column chromatography on silica gel (PE:DCM 95:5, 9:1) to yield **1c** as a dark red solid (1.64 g, 1.98 mmol, 79%).

^{1}H NMR (600 MHz, 295 K, $CDCl_3$) δ= 8.70-8.68 (m, 2H), 8.30 (s, 2H), 7.65-7.63 (m, 2H), 1.98-1.93 (m, 6 H), 1.52-1.47 (m, 6H), 1.28-1.27 (m, 18 H), 1.11-1.08 (m, 24H) ppm.

^{13}C{^{1}H} NMR (151 MHz, 295 K $CDCl_3$) δ= 143.2, 141.5, 135.9, 134.0, 128.6, 128.4, 127.9, 121.2, 110.1, 110.1, 102.7, 25.7, 25.6, 19.3, 19.3, 19.3, 14.8, 14.8, 14.8, 14.0, 13.9 ppm.

ATR-IR: $\tilde{\nu}$ [cm^{-1}] = 3650, 3629, 2954, 2921, 2869, 2359, 2344, 2339, 2331, 1457, 1389, 1044, 762, 694, 687, 682, 670, 648, 519, 490.

HR-MS (MALDI pos.) m/z: $[M+H]^+$: calcd. for $[C_{44}H_{60}N_2Si_2{}^{79}Br_2]^+$: 831.2735; found: 831.2742.

Melting point: > 300 °C.

$\mathbf{R_f}$ (PE:DCM 9:1) = 0.25

3,4-Dimethylhexane-6,11-bis((*sec*-butyl-l2-silyl)ethynyl)-2,3-bis((trimethylsilyl)ethynyl)benzo[*b*]phenazine (2c)

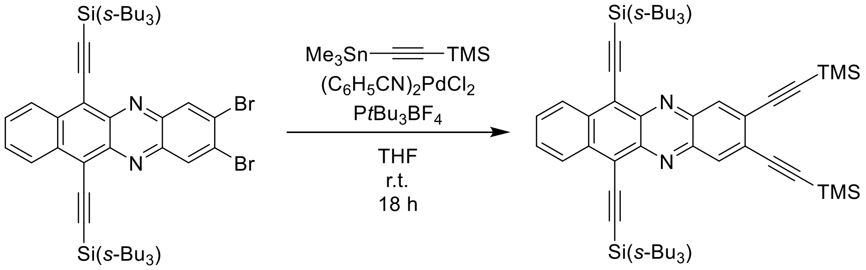

1c **2c**

In a flame dried Schlenk tube **1c** (1.64 g, 1.97 mmol, 1.00 eq) was dissolved in 100 mL dry THF under nitrogen atmosphere. The soltuion was degassed for 30 min. Trimethyl((trimethylstannyl)ethynyl)silane (2.57 g, 9.84 mmol, 5.00 eq.) was added and the resulting solution was degassed for additional 20 min. Subsequently bis(benzonitril)palladium (II) chloride (75.5 mg, 197 µmol, 10.0 mol%) and tri-*tert* butyl-phosphine-tetrafluoroborate (114 mg, 394 µmol, 20.0 mol%) were added. The reaction mixture was stirred for 12 h. The solvent was removed under reduced pressure and the crude product was purified by column chromatography on silica gel (PE:DCM 95:5, 9:1) to yield the product (1.65 g, 1.90 mmol, 97%) as a dark red solid.

^{1}H NMR (600 MHz, 295 K, $CDCl_3$) δ= 8.70-8.68 (m, 2Hs), 8.30 (s, 2Hs), 7.65-7.63 (m, 2Hs), 1.99-1.95 (m, 6Hs), 1.53-1.47 (m, 6Hs), 1.29-1.28 (m, 18Hs), 1.11-1.09 (m, 24Hs), 0.36 (s, 18 Hs) ppm.

^{13}C{^{1}H} NMR (151 MHz, 295 K, $CDCl_3$) δ= 143.6, 141.7, 135.7, 134.5, 128.3, 127.8, 127.1, 121.2, 109.7, 102.9, 102.8, 102.7, 25.7, 19.3, 14.8, 13.9, 13.9, 13.9, 0.1 ppm.

ATR-IR: $\tilde{\nu}$ [cm^{-1}] = 2956, 2926, 2868, 1458, 1453, 1441, 1429, 1410, 1389, 1369, 1265, 1250, 1187, 1149, 1098, 1042, 999, 961, 914, 889, 840, 794, 760, 742, 700, 687, 679, 661, 654, 632, 570, 517, 492, 482, 468 419.

HR-MS (MALDI pos.) m/z: [M]$^+$: calcd. for $[C_{54}H_{78}N_2Si_4]^+$: 866.5237; found: 866.5227.

Melting point: > 300 °C.

R$_f$ (PE:DCM 9:1) = 0.3

2,3-Diethynyl-6,11-bis((tri-*sec*-butylsilyl)ethynyl)benzo[*b*]phenazine (3c)

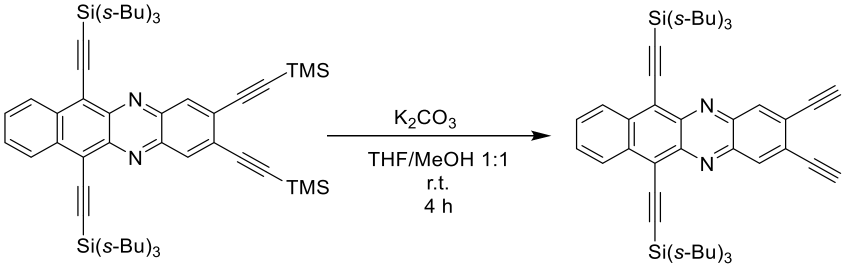

2c **3c**

2c (600 mg, 692 µmol, 1.00 eq.) was dissolved in a mixture of 40 mL THF and 40 mL MeOH. Subsequently K_2CO_3 (478 mg, 3.46 mmol, 5.00 eq.) was added and the reaction mixture was stirred for 4 h. Water was added and the mixture was extracted with DCM. The combined organic layers were dried over $MgSO_4$. to yield the product (460 mg, 637 µmol, 92%) as a dark red solid.

^{1}H NMR (400 MHz, 295 K, CD_2Cl_2) δ= 8.75-8.72 (m, 2H), 8.42 (s, 2H), 7.71-7.69 (m, 2H), 3.69 (s, 2H), 2.01-1.93 (m, 6 H), 1.57-1.49 (m ,6H), 1.32-1.30 (m, 18H), 1.13-1.10 (m, 24H) ppm.

^{13}C{^{1}H} NMR (101 MHz, 295 K, CD_2Cl_2) δ= 143.9, 142.1, 136.2, 135.4, 129.0, 128.1, 126.5, 121.6, 110.4, 103.1, 84.9, 81.5, 26.0, 19.7, 14.9, 14.1 ppm.

ATR-IR: $\tilde{\nu}$ [cm^{-1}] = 3306, 3283, 2954, 2928, 2868, 2738, 2726, 2362, 2357, 2344, 2331, 2323, 2173, 2164, 2160, 2154, 2143, 2138, 2131, 2105, 1507, 1457, 1442, 1435, 1410, 1389, 1369, 1338, 1312, 1283, 1264, 1226, 1213,

1186, 1169, 1147, 1104, 1095, 1041, 998, 965, 909, 886, 849, 806, 779, 682, 669, 661, 611, 592, 586, 552, 547, 537, 514, 510, 504, 497, 470, 466, 459, 453, 448, 443, 436, 430, 427, 423, 416, 412, 405.

HR-MS (MALDI pos.) m/z: [M+H]$^+$: calcd. for [$C_{48}H_{63}N_2Si_2$]$^+$: 723.4524; found: 723.4508.

Melting point: > 300 °C.

R$_f$ (PE:DCM 9:1) = 0.25

5,10-Bis{[tri(butan-2-yl)silyl]ethynyl}benzo[*g*]quinoxaline—5-[3,3-di(butan-2-yl)-4-methylhex-1-yn-1-yl]-22-{[tri(butan-2-yl)silyl]ethynyl}-8,9,10,11,16,17,18,19-octadehydrobenzo[*b*]benzo[7,8]cyclododeca[1,2-*i*]phenazine (3c$_2$)/5,10-Bis{[tri(butan-2-yl)silyl]ethynyl}benzo[*g*]quinoxaline—36-[3,3-di(butan-2-yl)-4-methylhex-1-yn-1-yl]-5,22,27-tris{[tri(butan-2-yl)silyl]ethynyl}-8,9,10,11,16,17,18,19,30,31,32,33-dodecadehydrobenzo[*b*]benzo[13,14]tetraceno[2',3':7,8]cyclooctadeca[1,2-*i*]phenazine (3c$_3$)

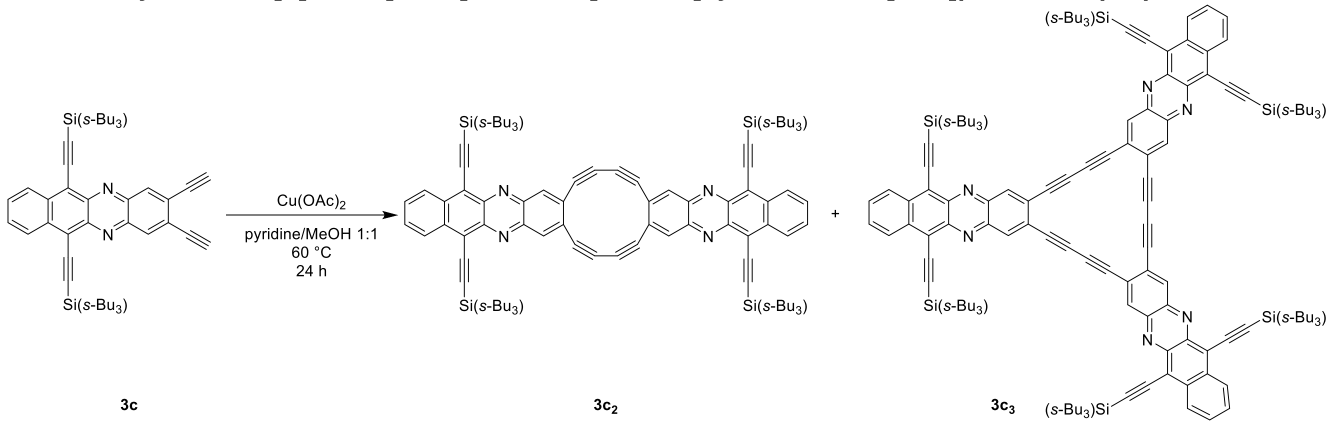

Copper (II) acetate (3.04 g, 15.2 mmol, 22.0 eq.) was dissolved in a mixture of 175 mL pyridine and 175 mL methanol. The mixture was heated to 60 °C. Afterwards a solution of **3c** (500 mg, 691 µmol, 1.00 eq.) in 20 mL of pyridine was added dropwise using a syringe pump (rate 0.06 mmol/h). After completion of the addition, the reaction mixture was stirred for additional 4 h. After cooling to room temperature the solvent was removed under reduced pressure. Water and DCM were added and the mixture was extracted with DCM. The combined organic layers were died over $MgSO_4$ and the solvent was removed under reduced pressure. The crude product was purified by column chromatography on Silica (PE:DCM 8:2, 7:3, 1:1) to yield a mixture of **3c$_2$** and **3c$_3$** containing some small impurities. Purification of the mixture using a GPC yield the dimer **3c**$_2$ (60.0 mg, 54.3 µmol, 12%) as a dark brown solid and the trimer **3c$_3$** (100 mg, 46.3 µmol, 20%) as a dark brown solid.

3c$_2$:

^{1}H NMR (600 MHz, 295 K, $CDCl_3$) δ= 8.67-8.65 (m, 4H), 8.06 (s, 4H), 7.54-7.52 (m, 4H), 2.00-1.96 (m, 12 H), 1.56-1.50 (m, 12H), 1.33-1.25 (m, 36 H), 1.17-1.10 (m, 48H) ppm.

^{13}C{^{1}H} NMR (151 MHz, 295 K $CDCl_3$) δ= 144.1, 142.0, 136.0, 131.0, 128.1, 127.9, 121.4, 110.1, 102.8, 92.3, 86.7, 25.7, 25.7, 19.3, 19.3, 19.3, 14.9, 14.9, 14.0, 14.0 ppm.

ATR-IR: $\tilde{\nu}$ [cm^{-1}] = 2953, 2923, 2867, 1450, 1417, 1389, 1369, 1335, 1261, 1186, 1144, 1095, 1042, 998, 965, 949, 883, 876, 849, 760, 700, 693, 683, 669, 661, 658, 653, 648, 644, 640, 634, 608, 512, 502, 498, 494, 487, 480, 471, 468, 461, 452, 442, 440, 431, 428, 423, 416, 408, 403.

HR-MS (MALDI pos.) m/z: [M+H]$^+$: calcd. for [$C_{96}H_{121}N_4Si_4$]$^+$: 1441.8663; found: 1441.8716.

Melting point: > 300 °C.

R$_f$ (PE:DCM 7:3) = 0.4

3c3:

^{1}H NMR (700 MHz, 295 K, $CDCl_3$) δ= 8.73-8.72 (m, 6H), 8.54 (s, 6H), 7.69-7.68 (m, 6H), 2.00-1.97 (m, 18 H), 1.55-1.50 (m, 18H), 1.34-1.33 (m, 54 H), 1.18-1.12 (m, 72H) ppm.

$^{13}C\{^1H\}$ NMR (176 MHz, 295 K $CDCl_3$) δ= 143.6, 141.9, 136.5, 136.1, 128.7, 127.9, 125.4, 121.5, 110.3, 110.3, 102.8, 82.1, 81.3, 25.7, 19.3, 19.3, 14.9, 14.0, 14.0 ppm.

ATR-IR: $\tilde{\nu}$ [cm^{-1}] = 2952, 2927, 2922, 2867, 1457, 1429, 1388, 1369, 1328, 1262, 1186, 1163, 1149, 1096, 1042, 998, 964, 950, 887, 849, 760, 702, 689, 677, 652, 648, 622, 616, 517, 510, 502, 493 ,479, 475, 469, 464, 461, 458, 454, 447, 444, 438, 430, 420, 417, 413, 406.

HR-MS (MALDI pos.) m/z: $[M+H]^+$: calcd. for $[C_{144}H_{181}N_6Si_6]^+$: 2162.2958; found: 2162.2910.

Melting point: > 300 °C.

R_f (PE:DCM 7:3) = 0.3

2. Results and Discussion

2.1 Calculations

2.1.1 FMO and AICD calculation

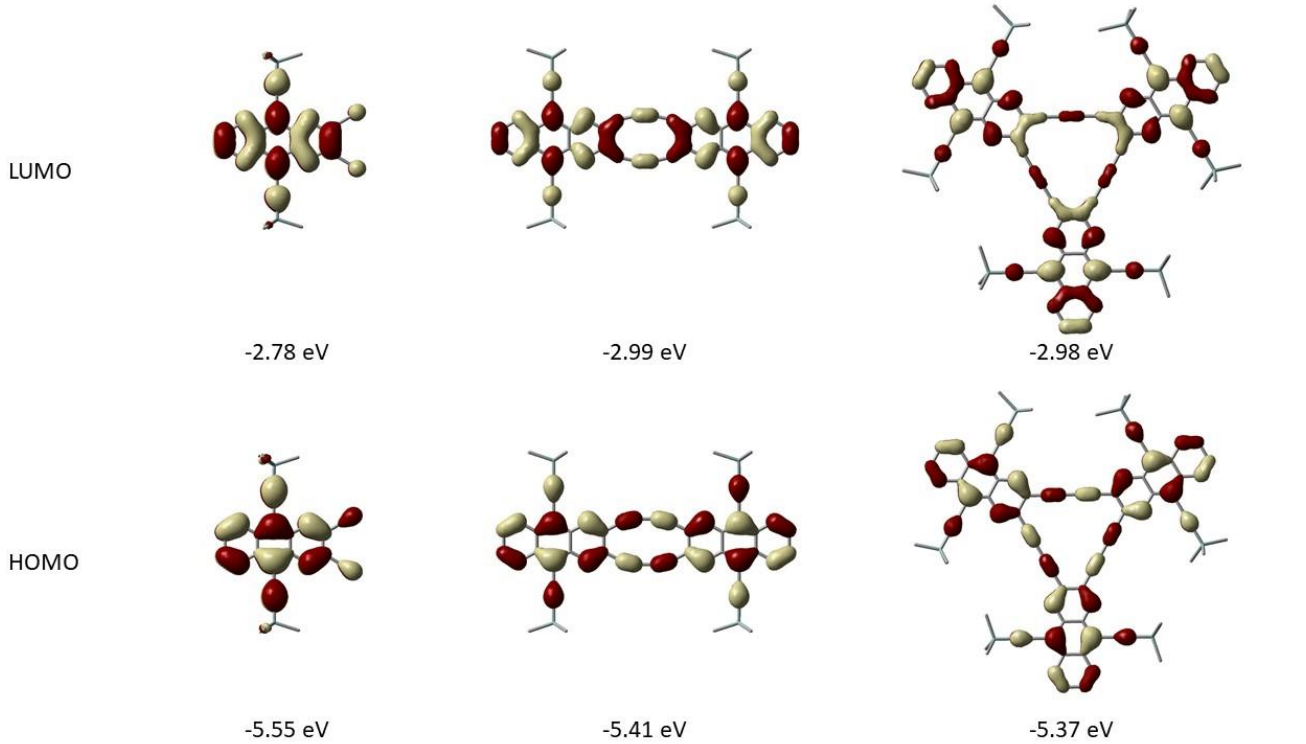

Figure S1. Calculated FMOs of **3a** (left), **3a₂** (middle) and **3a₃** (right).

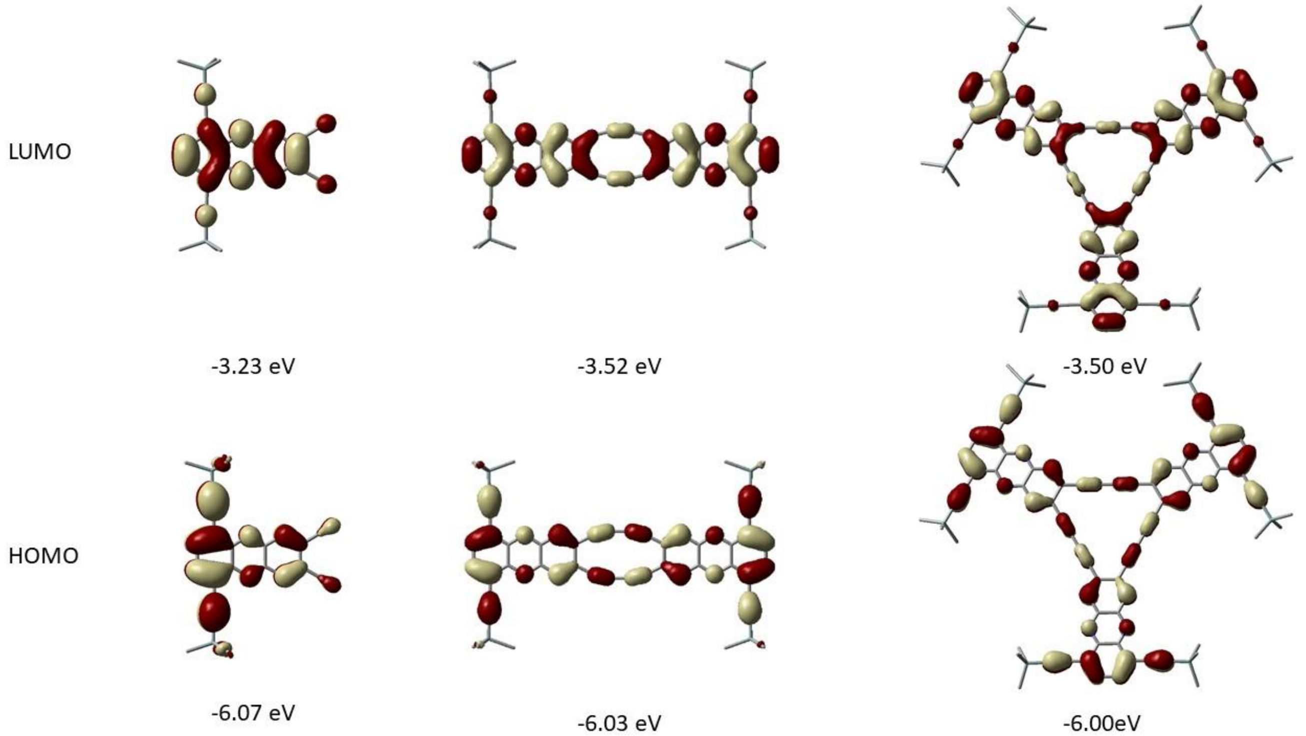

Figure S2. Calculated FMOs of **3b** (left), **3b₂** (middle) and **3b₃** (right)

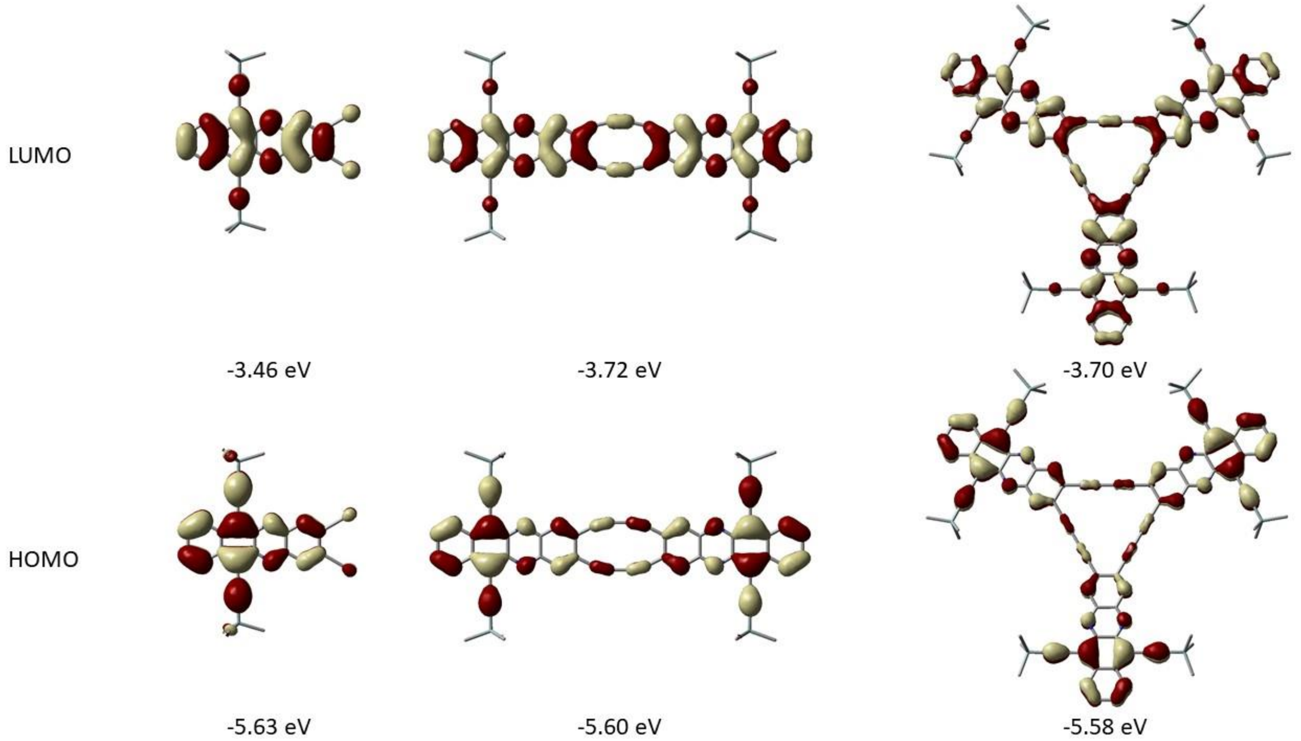

Figure S3. Calculated FMOs of **3c** (left), **3c$_2$** (middle) and **3c$_3$** (right).

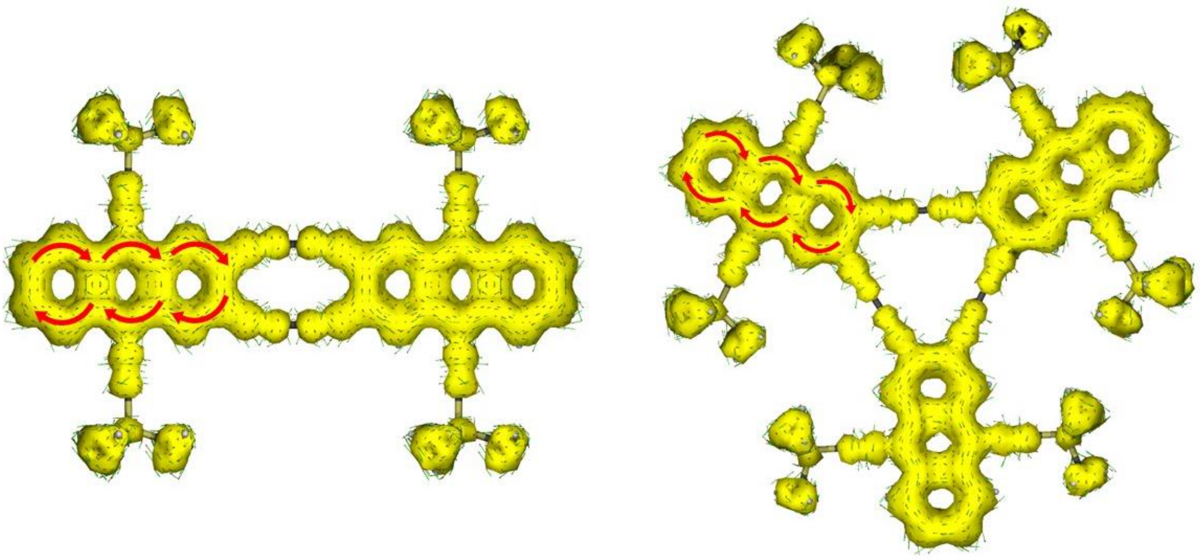

Figure S4 AICD plot of 3a$_2$ (left) and 3a$_3$ (right). Arrows indicate the ring current flow magnetic field, magnetic field vector is oriented out of plane.

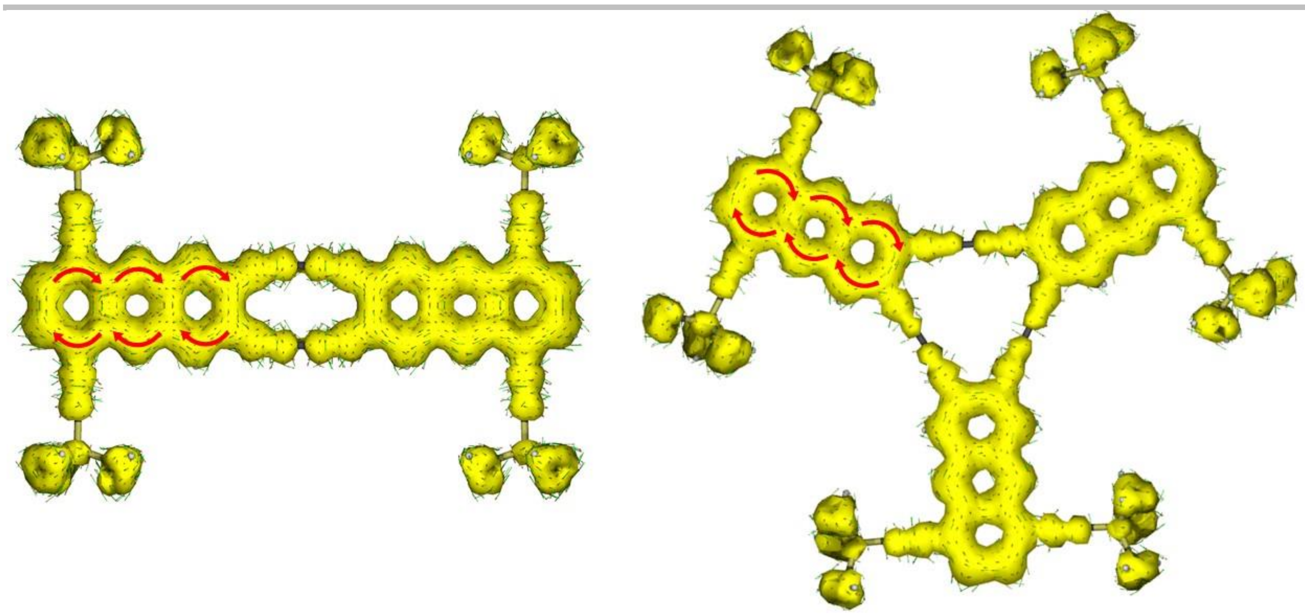

Figure S5. AICD plot of **3b$_2$** (left) and **3b$_3$** (right). Arrows indicate the ring current flow magnetic field, magnetic field vector is oriented out of plane.

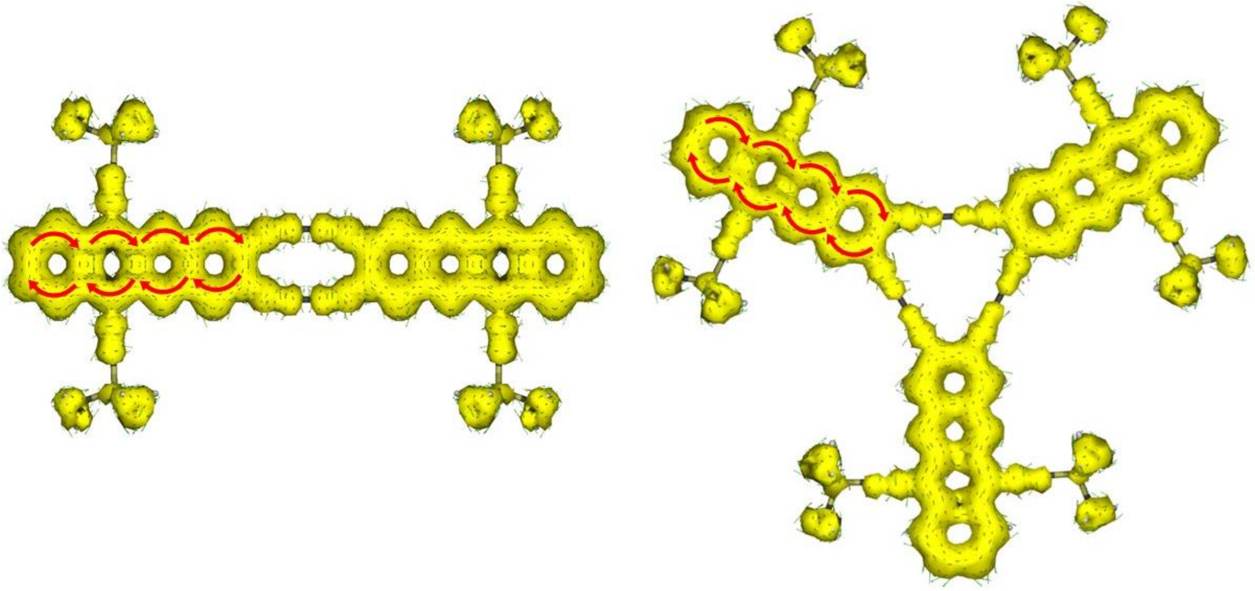

Figure S6. AICD plot of **3c$_2$** (left) and **3c$_3$** (right). Arrows indicate the ring current flow magnetic field, magnetic field vector is oriented out of plane.

2.1.2 Calculation of Transfer Integrals

Transfer integrals were determined with the Amsterdam Density Functional (ADF)[S6, S7] using a dimer pair of the crystal structure at the GGA-pw91/DZP level of theory. If there are various neighbor molecules, the transfer integrals off all possible neighbor combinations were calculated.

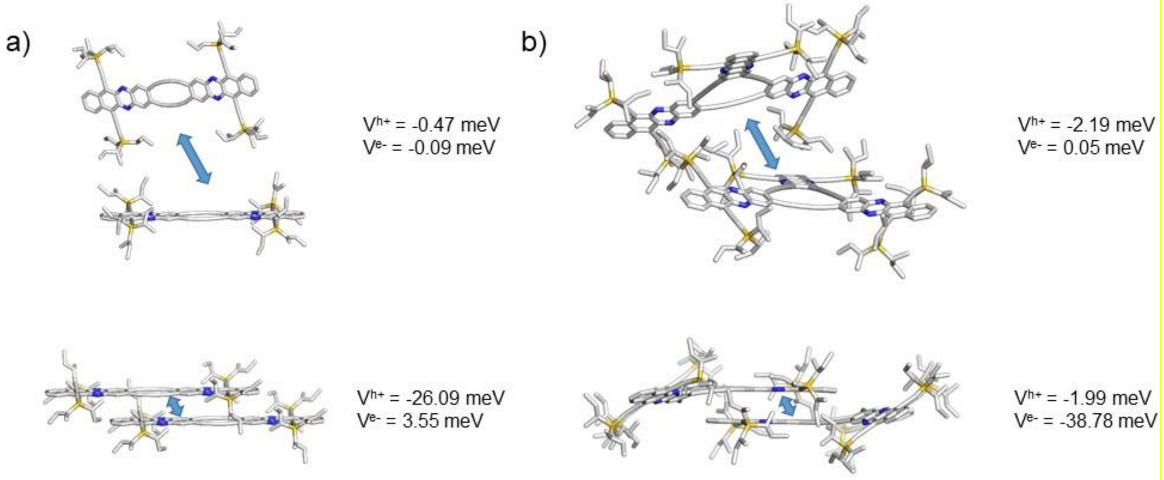

Figure S7. Dimer pairs of a) **3c₂** and b) **3c₃** for the calculation of the transfer integrals.

2.1.3 Coordinates of the Optimized Geometries

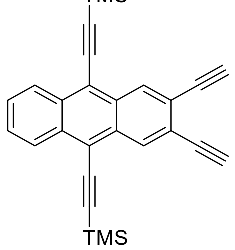

3a

:

C	0.70783400	-4.27352500	0.00025400
C	-0.70795000	-4.27350400	0.00028600
C	-1.39646100	-3.09497100	0.00026400
C	-0.71726900	-1.84482700	0.00020200
C	0.71721400	-1.84484600	0.00017000
C	1.39637700	-3.09500800	0.00020200
C	-1.42415600	-0.61736600	0.00015500
C	-0.71636300	0.60800700	0.00011900
C	0.71638100	0.60799100	0.00008800
C	1.42414100	-0.61739700	0.00009500
C	-1.39174400	1.85457500	0.00012500
C	-0.72190700	3.05355300	0.00011300
C	0.72199500	3.05353400	0.00008100
C	1.39179700	1.85454000	0.00006500
C	2.84279700	-0.61486300	0.00001500
C	4.05774300	-0.60806400	-0.00005900
C	-2.84281200	-0.61479300	0.00014600
C	-4.05775300	-0.60796400	0.00010000
C	1.44782600	4.27725100	0.00007800
C	2.06994100	5.30621700	0.00009700
C	-1.44770000	4.27729000	0.00014800
C	-2.06984000	5.30624200	0.00014300
Si	5.89972700	-0.59003800	-0.00015900
C	6.47334200	1.19782900	0.00041000
C	6.50121000	-1.47597000	-1.54360900
C	6.50153800	-1.47706800	1.54253800
Si	-5.89974100	-0.58999400	-0.00016600
C	-6.47339600	1.19786100	0.00028200
C	-6.50165200	-1.47700800	1.54249600

C	-6.50107300	-1.47597100	-1.54365100
H	1.24423900	-5.21394900	0.00027000
H	-1.24438100	-5.21391300	0.00032500
H	-2.47748700	-3.09115300	0.00028700
H	2.47740000	-3.09121600	0.00017800
H	-2.47208500	1.85660200	0.00014900
H	2.47214000	1.85653400	0.00004400
H	2.61353800	6.21872600	0.00016000
H	-2.61344000	6.21875000	0.00021600
H	6.11135500	1.72932600	0.88287800
H	7.56510500	1.25243100	0.00070400
H	6.11186700	1.72961100	-0.88210500
H	6.15490800	-2.51132900	-1.56604500
H	7.59368400	-1.48689600	-1.58145200
H	6.13851500	-0.98319300	-2.44789800
H	6.13938900	-0.98468300	2.44726200
H	6.15500800	-2.51235500	1.56456900
H	7.59402900	-1.48825200	1.57989100
H	-6.11185600	1.72960900	-0.88222500
H	-7.56516300	1.25241000	0.00045900
H	-6.11153500	1.72942500	0.88276100
H	-6.15509000	-2.51228200	1.56457700
H	-7.59414600	-1.48822600	1.57977600
H	-6.13958100	-0.98458800	2.44723300
H	-6.13830600	-0.98317500	-2.44790300
H	-6.15473800	-2.51131900	-1.56606500
H	-7.59354200	-1.48693100	-1.58160000

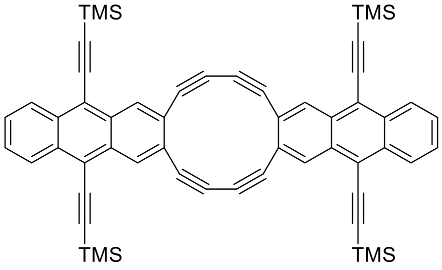

$3a_2$

C	10.44612100	-0.70732400	0.00010300
C	10.44611700	0.70735700	0.00009900
C	9.26664100	1.39602900	0.00013000
C	8.01791300	0.71667500	0.00016400
C	8.01791700	-0.71665400	0.00016800
C	9.26664800	-1.39600100	0.00013700
C	6.78836800	1.42370700	0.00019300
C	5.56425100	0.71730200	0.00020300
C	5.56425400	-0.71729300	0.00020800
C	6.78837400	-1.42369200	0.00020300
C	4.31823100	1.39801800	0.00019600
C	3.11981000	0.73138800	0.00018600
C	3.11981300	-0.73139000	0.00018800
C	4.31823700	-1.39801400	0.00020200
C	1.86471900	-1.38414600	0.00016000
C	0.68039200	-1.65821000	0.00012000
C	1.86471400	1.38413900	0.00015700
C	0.68038600	1.65820300	0.00011700
C	-0.67372500	1.65821400	0.00006300
C	-1.85810000	1.38430100	0.00000900
C	-0.67371800	-1.65820800	0.00006500

C	-1.85809200	-1.38428700	0.00000900
C	-3.11310900	-0.73138300	-0.00005700
C	-3.11311400	0.73139100	-0.00005800
C	-4.31156900	1.39786000	-0.00012500
C	-5.55764700	0.71732300	-0.00019600
C	-5.55764300	-0.71733000	-0.00019600
C	-4.31156000	-1.39786000	-0.00012500
C	-6.78175300	1.42367000	-0.00026500
C	-8.01131500	0.71661200	-0.00033500
C	-8.01131000	-0.71663500	-0.00033700
C	-6.78174400	-1.42368600	-0.00026800
C	-9.25996500	1.39613100	-0.00041000
C	-10.43942200	0.70732500	-0.00048000
C	-10.43941800	-0.70736300	-0.00047900
C	-9.25995600	-1.39616200	-0.00041000
C	6.78673800	2.84246600	0.00021200
C	6.78083600	4.05738700	0.00023600
C	6.78675200	-2.84245200	0.00023600
C	6.78085500	-4.05737200	0.00027300
C	-6.78125100	-2.84242100	-0.00027600
C	-6.77896800	-4.05734700	-0.00028500
C	-6.78126900	2.84240700	-0.00026400
C	-6.77899200	4.05733200	-0.00026500
Si	6.76379600	5.89998600	0.00030600
C	4.97635600	6.47415100	-0.00272000
C	7.64829300	6.49983900	1.54508900
C	7.65357500	6.50000400	-1.54137800
Si	6.76382800	-5.89997200	0.00037500
C	4.97639500	-6.47414900	-0.00405500
C	7.65483600	-6.50002000	-1.54058800
C	7.64710100	-6.49978200	1.54587500
Si	-6.77167300	-5.89994700	-0.00031000
C	-5.87923400	-6.48930400	1.54328800
C	-8.55348000	-6.49491300	-0.00015500
C	-5.87951700	-6.48926200	-1.54408700
Si	-6.77171700	5.89993200	-0.00026000
C	-8.55353000	6.49487900	-0.00213000
C	-5.88104900	6.48927800	1.54436500
C	-5.87780300	6.48927600	-1.54300800
H	11.38631200	-1.24408800	0.00007700
H	11.38630600	1.24412600	0.00007100
H	9.26280700	2.47704300	0.00012600
H	9.26281900	-2.47701600	0.00014000
H	4.32066800	2.47849000	0.00018700
H	4.32068000	-2.47848700	0.00019400
H	-4.31415000	2.47828100	-0.00012600
H	-4.31413400	-2.47828100	-0.00012600
H	-9.25628300	2.47717900	-0.00041200
H	-11.37959200	1.24413000	-0.00053700
H	-11.37958500	-1.24417400	-0.00053400
H	-9.25626700	-2.47720900	-0.00041000
H	4.92249000	7.56594300	-0.00281500
H	4.44569100	6.11324600	-0.88613100
H	4.44271600	6.11326300	0.87890600
H	7.65998700	7.59228100	1.58332100
H	8.68335200	6.15275500	1.56884000
H	7.15392900	6.13734800	2.44858600
H	7.16237400	6.13751300	-2.44659900
H	7.66528500	7.59244900	-1.57951100
H	8.68874900	6.15303800	-1.56157000

H	4.92253600	-7.56594100	-0.00415900
H	4.44205200	-6.11324000	0.87713600
H	4.44642800	-6.11327300	-0.88789800
H	7.66660500	-7.59246600	-1.57867200
H	8.69001700	-6.15302900	-1.55997800
H	7.16433700	-6.13757400	-2.44620700
H	8.68213300	-6.15267400	1.57044600
H	7.15200900	-6.13728400	2.44897000
H	7.65878600	-7.59222300	1.58413500
H	-5.85429300	-7.58154400	1.58113000
H	-4.84844500	-6.12983600	1.56566100
H	-6.37661300	-6.13289100	2.44756300
H	-8.59534800	-7.58720000	-0.00014900
H	-9.08908700	-6.14001600	-0.88311700
H	-9.08893600	-6.14001100	0.88289600
H	-6.37707100	-6.13283700	-2.44826200
H	-4.84873700	-6.12977900	-1.56664700
H	-5.85456800	-7.58150100	-1.58195700
H	-8.59540900	7.58716500	-0.00226200
H	-9.08816000	6.13989800	-0.88564900
H	-9.08995500	6.14005000	0.88036400
H	-5.85609900	7.58151800	1.58221900
H	-4.85030500	6.12976000	1.56793900
H	-6.37949400	6.13290200	2.44806800
H	-4.84698200	6.12984100	-1.56438000
H	-6.37430000	6.13282900	-2.44775500
H	-5.85285900	7.58151700	-1.58085200

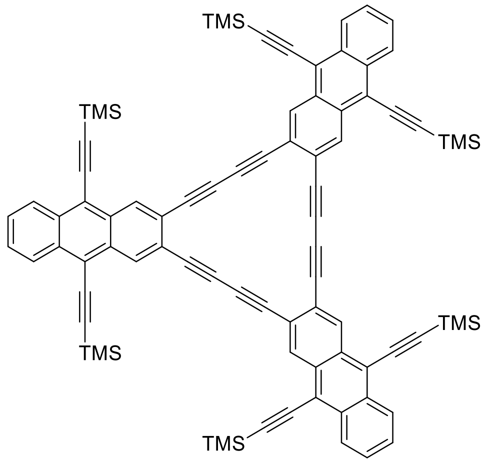

$3a_3$

C	11.48815200	-1.45424700	-0.01158400
C	11.58060400	-0.04188900	-0.01540100
C	10.44912300	0.72229800	-0.01516700
C	9.15781100	0.12609900	-0.01119200
C	9.06414200	-1.30500500	-0.00736600
C	10.26672400	-2.06443900	-0.00764600
C	7.97848800	0.91205400	-0.01079800
C	6.70947100	0.28659300	-0.00712300
C	6.61579500	-1.14493800	-0.00349900
C	7.79246800	-1.93051300	-0.00334600
C	5.51131800	1.04245500	-0.00700100
C	4.26732700	0.45400900	-0.00387700
C	4.17265300	-0.99271200	-0.00069100
C	5.32938800	-1.73824000	-0.00041200

C	7.70105300	-3.34598900	0.00109400
C	7.62766800	-4.55873500	0.00512200
C	8.07251500	2.32736600	-0.01406600
C	8.15719900	3.53938100	-0.01666400
C	2.91047900	-1.62679200	0.00127600
C	3.09811100	1.24652700	-0.00338800
C	2.09005300	1.92405500	-0.00249000
C	1.82366000	-2.16904300	0.00196600
C	-7.00156500	-9.23128200	0.00229200
C	-5.82299600	-10.01504700	0.00749400
C	-4.59677900	-9.41463200	0.00871000
C	-4.47028200	-7.99798000	0.00483100
C	-5.66455900	-7.20378800	-0.00045800
C	-6.92211600	-7.86825900	-0.00155600
C	-3.20144800	-7.36689500	0.00596700
C	-3.11166900	-5.95487600	0.00216800
C	-4.30616800	-5.16050300	-0.00334800
C	-5.57342900	-5.78948100	-0.00495500
C	-1.85935900	-5.29264900	0.00345000
C	-1.74975800	-3.92079700	0.00064100
C	-2.95693600	-3.11797700	-0.00404600
C	-4.17963200	-3.74953500	-0.00640600
C	-6.75532100	-5.00522300	-0.01134900
C	-7.76981000	-4.33668400	-0.01734000
C	-2.01985400	-8.15159500	0.01119800
C	-1.00696600	-8.82256400	0.01521200
C	-2.87733800	-1.70770800	-0.00572200
C	-0.47952600	-3.30298900	0.00188300
C	0.61143400	-2.76911200	0.00227600
C	-2.80437500	-0.49530400	-0.00607100
C	-4.49251900	10.68149200	0.03258800
C	-5.76154200	10.05463700	0.03085900
C	-5.85677600	8.69265000	0.02343700
C	-4.69435000	7.87308600	0.01739500
C	-3.40840400	8.50826000	0.01939700
C	-3.35299000	9.92944400	0.02696500
C	-4.78467700	6.45884200	0.00999400
C	-3.60805300	5.67313900	0.00585000
C	-2.32186800	6.30841100	0.00723900
C	-2.23052000	7.72030300	0.01353400
C	-3.66278100	4.25759200	0.00107300
C	-2.53079400	3.47500000	-0.00167200
C	-1.23096300	4.11697700	-0.00061800
C	-1.16440500	5.49161300	0.00319900
C	-0.95848200	8.34787900	0.01356600
C	0.13143100	8.88477200	0.01304500
C	-6.05669500	5.83122000	0.00639300
C	-7.14704200	5.29520900	0.00274900
C	-0.05008800	3.34191500	-0.00205400
C	-2.63260700	2.06616600	-0.00436300
C	-2.71692000	0.85450600	-0.00562200
C	0.96390100	2.67330400	-0.00236200
Si	8.28857200	5.37660100	-0.02032600
C	10.11058400	5.83414500	-0.00266600
C	7.42922600	6.03699200	1.51318600
C	7.46127500	6.02905300	-1.57470600
Si	7.52703600	-6.39784900	0.01084200
C	6.58000300	-6.93606300	1.54048500
C	9.27667100	-7.08115800	0.04498400
C	6.63519500	-6.94959500	-1.54683700

Si	0.53330700	-9.83238600	0.01987100
C	0.45152400	-11.04548600	-1.41190300
C	0.63576700	-10.75163800	1.65472800
C	1.99784100	-8.67449300	-0.17882400
Si	-9.31155000	-3.32909500	-0.02599800
C	-9.27790900	-2.20453600	-1.52943400
C	-9.37025700	-2.31787400	1.55518400
C	-10.77729200	-4.50116700	-0.11165000
Si	-8.79915300	4.48110800	-0.00765700
C	-8.74369000	3.01402900	1.16288800
C	-9.17136200	3.91504100	-1.75936200
C	-10.07698900	5.73355300	0.56444000
Si	1.78578500	9.69428900	0.01175600
C	3.09674200	8.35760000	-0.12749800
C	1.98150000	10.64667000	1.61890800
C	1.87359500	10.86079600	-1.45799200
H	12.39128200	-2.05122700	-0.01177400
H	12.55384400	0.43226400	-0.01847000
H	10.51623600	1.80120300	-0.01801600
H	10.19266300	-3.14289900	-0.00469200
H	5.58106100	2.12056800	-0.00959800
H	5.25809500	-2.81623400	0.00177200
H	-7.96910600	-9.71695500	0.00126100
H	-5.89679000	-11.09512900	0.01045800
H	-3.69464800	-10.01017300	0.01267300
H	-7.82030400	-7.26680400	-0.00574300
H	-0.95924400	-5.89011500	0.00659200
H	-5.07876300	-3.15060800	-0.01019000
H	-4.42780300	11.76214300	0.03845400
H	-6.65911800	10.65990800	0.03569300
H	-6.82440500	8.21074500	0.02264000
H	-2.38221500	10.40493800	0.02840500
H	-4.63102600	3.77841300	0.00033500
H	-0.19532100	5.96915600	0.00395300
H	10.62641300	5.43755300	-0.87956300
H	10.23578100	6.91999700	-0.00377100
H	10.60835500	5.44134500	0.88628400
H	6.37466400	5.75478800	1.52767600
H	7.48514100	7.12815800	1.54725900
H	7.89155700	5.64829400	2.42267000
H	7.94010400	5.63283200	-2.47232900
H	6.40630000	5.75001700	-1.60799200
H	7.52134000	7.11979400	-1.61498000
H	5.56930600	-6.52323600	1.54501900
H	6.49781900	-8.02537900	1.58012300
H	7.08070800	-6.60349700	2.45190100
H	9.84421700	-6.75867500	-0.83036000
H	9.26408500	-8.17410800	0.05126700
H	9.81400400	-6.74804900	0.93527100
H	6.55695100	-8.03939000	-1.58107700
H	5.62429700	-6.53952900	-1.58991200
H	7.16684300	-6.62264500	-2.44263300
H	0.37217600	-10.52324100	-2.36737400
H	1.34965100	-11.66785900	-1.44228100
H	-0.41162300	-11.70801500	-1.32081900
H	0.66357100	-10.05707800	2.49661300
H	1.53860200	-11.36658300	1.69589300
H	-0.22446500	-11.40965300	1.79333800
H	1.93741900	-8.11768600	-1.11603000
H	2.04377200	-7.95148900	0.63803100

H	2.93546100	-9.23623700	-0.18225000
H	-9.22611800	-2.78308700	-2.45384800
H	-10.17839300	-1.58611800	-1.56973800
H	-8.41379300	-1.53761400	-1.50476300
H	-9.36438800	-2.96279400	2.43611000
H	-10.27721400	-1.70867300	1.59040800
H	-8.51242500	-1.64641900	1.62774000
H	-11.71723400	-3.94336200	-0.12343300
H	-10.74125400	-5.11454100	-1.01429200
H	-10.79793700	-5.17183000	0.74981800
H	-8.50127700	3.32883900	2.17976700
H	-9.71072600	2.50514600	1.19011900
H	-7.99129700	2.28716000	0.85039200
H	-9.17922700	4.75801700	-2.45316400
H	-10.14947100	3.42942100	-1.80906400
H	-8.42385400	3.20106700	-2.11079100
H	-11.07466700	5.28726000	0.57939300
H	-9.85539600	6.09257500	1.57147200
H	-10.10869300	6.59883500	-0.10091700
H	2.97870600	7.78262100	-1.04811000
H	4.09710600	8.79793700	-0.13304800
H	3.04199200	7.66131400	0.71172100
H	1.20700300	11.40909500	1.72407300
H	2.95239100	11.14766100	1.65521800
H	1.91415600	9.98143700	2.48198800
H	2.84327300	11.36418200	-1.49371200
H	1.73997900	10.32175400	-2.39795800
H	1.09970400	11.62894700	-1.40077100

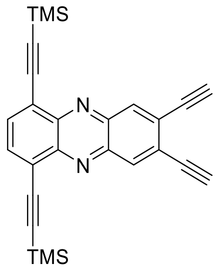

3b

C	0.70355000	-2.98815400	-0.00004400
C	-0.70355800	-2.98815200	-0.00004400
C	-1.43489800	-1.81872900	-0.00003000
C	-0.72198900	-0.56549700	-0.00001400
C	0.72198700	-0.56549900	-0.00001600
C	1.43489200	-1.81873200	-0.00003100
N	-1.40812900	0.57525100	0.00000100
C	-0.71762100	1.71761800	0.00001500
C	0.71762400	1.71761600	0.00001400
N	1.40812900	0.57524800	-0.00000200
C	-1.40710500	2.95716600	0.00003300
C	-0.72621700	4.14899600	0.00004900
C	0.72622500	4.14899500	0.00004800
C	1.40711000	2.95716300	0.00003100
C	2.85242100	-1.84358400	-0.00003500
C	4.06451000	-1.89710600	-0.00003900
C	-2.85242600	-1.84357700	-0.00003100
C	-4.06451500	-1.89709700	-0.00003100
C	1.44094900	5.37897700	0.00006600
C	2.05553300	6.41200500	0.00007700

C	-1.44093800	5.37898000	0.00006600
C	-2.05551500	6.41201200	0.00009100
Si	5.90479300	-1.92934200	-0.00001500
C	6.51059200	-1.04579500	-1.54279200
C	6.47014100	-3.72095100	-0.00187300
C	6.51049400	-1.04903500	1.54465400
Si	-5.90479800	-1.92933200	-0.00002400
C	-6.51053800	-1.04834400	-1.54428900
C	-6.51055700	-1.04646800	1.54316000
C	-6.47014900	-3.72094200	0.00105200
H	1.22939800	-3.93333700	-0.00005500
H	-1.22940900	-3.93333400	-0.00005600
H	-2.48799500	2.93835600	0.00003400
H	2.48800000	2.93835100	0.00002900
H	2.59381000	7.32793000	0.00000100
H	-2.59368900	7.32799800	0.00030000
H	6.16231000	-0.01125900	-1.56451300
H	7.60313100	-1.03507000	-1.57907400
H	6.14885000	-1.53856500	-2.44752000
H	6.10454700	-4.25103100	0.88007900
H	7.56150700	-3.78291600	-0.00189000
H	6.10462300	-4.24917900	-0.88496700
H	6.16214400	-0.01457100	1.56856400
H	6.14876700	-1.54375100	2.44832600
H	7.60303300	-1.03831200	1.58098300
H	-6.14867900	-1.54255500	-2.44818400
H	-7.60307500	-1.03776800	-1.58068000
H	-6.16234200	-0.01381500	-1.56766300
H	-6.14895500	-1.53975500	2.44766400
H	-7.60309700	-1.03558300	1.57937600
H	-6.16210700	-0.01200100	1.56542600
H	-7.56151500	-3.78290400	0.00119800
H	-6.10468500	-4.25059700	-0.88120900
H	-6.10450400	-4.24959600	0.88383800

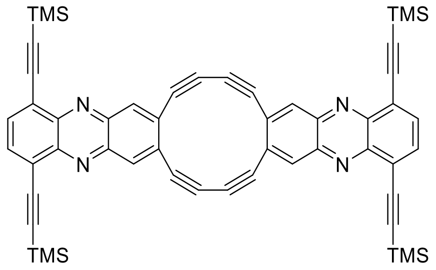

$3b_2$

C	-10.23586300	-0.70156400	0.02435500
C	-10.23469800	0.70436300	0.00341700
C	-9.06409000	1.43567700	-0.00543300
C	-7.81321800	0.72111600	0.00685900
C	-7.81457000	-0.72190100	0.02521400
C	-9.06636900	-1.43457000	0.03576300
N	-6.66943400	1.40581100	0.00103600
C	-5.52866800	0.71587600	0.01011100
C	-5.52992000	-0.72103800	0.02386000
N	-6.67204600	-1.40879300	0.03244200
C	-4.28910700	1.40978900	0.00615400
C	-3.09819000	0.73087200	0.01241700
C	-3.09960600	-0.74042800	0.02120200

C	-4.29168600	-1.41726800	0.02773700
C	-1.84117700	-1.38625100	0.02150600
C	-0.65755500	-1.66101100	0.01835100
C	-1.83840100	1.37412500	0.01080000
C	-0.65406400	1.64588500	0.01201200
C	0.69971700	1.64433500	0.01339200
C	1.88340000	1.36974800	0.01470500
C	0.69619000	-1.66264100	0.01419400
C	1.88046000	-1.39069000	0.00996800
C	3.14023500	-0.74748300	0.00768400
C	3.14183500	0.72383300	0.01319700
C	4.33413000	1.40035500	0.01600700
C	5.57227900	0.70393200	0.01145000
C	5.57066700	-0.73297400	0.00128500
C	4.33099800	-1.42664300	0.00095500
N	6.71445100	1.39155800	0.01633200
C	7.85681000	0.70449200	0.00950300
C	7.85516400	-0.73869400	-0.00488700
N	6.71125200	-1.42317500	-0.00763100
C	9.10902400	1.41670600	0.01651500
C	10.27816700	0.68311000	0.00637900
C	10.27654800	-0.72285200	-0.01033700
C	9.10571000	-1.45377600	-0.01640300
C	9.12848300	-2.87129900	-0.03520600
C	9.17850900	-4.08369500	-0.05365200
C	9.13516200	2.83417100	0.03496900
C	9.18817100	4.04644700	0.05282600
C	-9.09114600	-2.85202200	0.05921300
C	-9.08736700	2.85312300	-0.02758900
C	-9.13772100	4.06546300	-0.04846100
C	-9.14113600	-4.06437100	0.07992500
Si	9.21245300	5.89495800	0.08293200
C	9.02951500	6.42128600	-1.74401500
C	10.90093800	6.36057700	0.84365500
C	7.72304400	6.38410300	1.17173800
C	10.23793900	5.98727900	-2.58970100
C	8.69631400	7.90202900	-1.98507200
C	11.01697600	5.89677500	2.30497600
C	11.31869500	7.83279300	0.70320900
C	6.39013700	5.95142700	0.53879200
C	7.66889300	7.85347000	1.61848100
Si	9.19828400	-5.93225100	-0.08487700
C	7.70657600	-6.41715300	-1.17243600
C	9.01590600	-6.45922700	1.74194000
C	10.88487600	-6.40144900	-0.84759800
C	6.37537700	-5.98160500	-0.53787000
C	7.64838500	-7.88612900	-1.61994700
C	10.22620000	-6.02861700	2.58668100
C	8.67940300	-7.93931500	1.98243800
C	11.00053400	-5.93703700	-2.30875200
C	11.29930900	-7.87473500	-0.70848600
Si	-9.17377200	-5.90992900	0.15961200
C	-10.27156300	-6.29371500	1.67780800
C	-7.36724800	-6.46292600	0.45663600
C	-9.82333100	-6.54958800	-1.52381800
C	-10.20462200	-7.73169400	2.21516900
C	-11.73160800	-5.85184700	1.48928400
C	-6.35961500	-5.63388300	-0.35720300
C	-7.10992300	-7.96224500	0.23035600
C	-10.87067000	-5.63780000	-2.18280500

C	-10.33116700	-8.00151300	-1.48744300
Si	-9.15781000	5.91402900	-0.08264500
C	-10.84503500	6.38179300	-0.84477000
C	-7.66698300	6.39743900	-1.17204300
C	-8.97408600	6.44362900	1.74327600
C	-10.96168200	5.91531800	-2.30518900
C	-11.25959600	7.85520800	-0.70739100
C	-6.33524200	5.96303000	-0.53783300
C	-7.60935400	7.86576600	-1.62176800
C	-10.18369400	6.01416700	2.58958200
C	-8.63751200	7.92409800	1.98134500
H	-11.18148600	-1.22649000	0.03176900
H	-11.17942100	1.23092100	-0.00567800
H	-4.31072900	2.49072600	-0.00207800
H	-4.31509000	-2.49815800	0.03487500
H	4.35792600	2.48126700	0.02152000
H	4.35236800	-2.50760300	-0.00470600
H	11.22392500	1.20787900	0.01132300
H	11.22109200	-1.24975900	-0.01865500
H	8.16882700	5.83498700	-2.08890900
H	11.61343900	5.76478700	0.25924500
H	7.86154700	5.77586500	2.07431700
H	10.45567100	4.92387300	-2.47452100
H	10.05130600	6.17360000	-3.65170600
H	11.13953000	6.54299900	-2.31950800
H	7.77228500	8.20387900	-1.49023500
H	8.56821200	8.09251000	-3.05521400
H	9.48960400	8.56527200	-1.63427700
H	10.77270800	4.83894300	2.41867900
H	12.03575200	6.04395800	2.67642100
H	10.35088200	6.46321600	2.96069100
H	11.35794800	8.15453300	-0.33836400
H	12.31543700	7.98708100	1.12819600
H	10.63852100	8.50523600	1.22995100
H	6.39393000	4.89386900	0.26893500
H	5.56306700	6.11513200	1.23653600
H	6.16788100	6.52699500	-0.36353500
H	8.56088800	8.15154300	2.17136300
H	6.80967000	8.01829200	2.27603300
H	7.56136300	8.53704000	0.77385800
H	7.84562800	-5.80874100	-2.07481400
H	8.15696400	-5.87107400	2.08803000
H	11.59937500	-5.80770700	-0.26354100
H	6.38201100	-4.92421600	-0.26740900
H	5.54719200	-6.14290200	-1.23485000
H	6.15266900	-6.55715400	0.36435900
H	8.53914900	-8.18610100	-2.17378000
H	6.78817300	-8.04845400	-2.27682500
H	7.53990800	-8.56990000	-0.77560500
H	10.44633900	-4.96566000	2.47194000
H	10.04019400	-6.21515400	3.64875800
H	11.12619600	-6.58630700	2.31523500
H	7.75417300	-8.23866600	1.48832700
H	8.55189900	-8.13013700	3.05259200
H	9.47076500	-8.60423800	1.63046700
H	10.75864600	-4.87856400	-2.42157300
H	12.01858300	-6.08640000	-2.68132000
H	10.33244300	-6.50151000	-2.96413000
H	11.33888200	-8.19723000	0.33284200
H	12.29524900	-8.03109700	-1.13459300

H	10.61702300	-8.54524700	-1.23495800
H	-9.83639300	-5.64270500	2.44762000
H	-7.18593800	-6.24950000	1.51823600
H	-8.93325400	-6.53113400	-2.16669000
H	-9.18861200	-8.02597700	2.48183300
H	-10.81610200	-7.82685400	3.11794300
H	-10.58331600	-8.45815800	1.49314400
H	-11.80593400	-4.81721700	1.14999300
H	-12.28190800	-5.93146400	2.43189400
H	-12.25049400	-6.47983100	0.76119600
H	-6.45324800	-4.56583100	-0.16171700
H	-5.33569000	-5.93822900	-0.11766000
H	-6.49477300	-5.78603100	-1.43204700
H	-7.77882400	-8.60334200	0.80570400
H	-6.08541300	-8.21888000	0.51628300
H	-7.22171500	-8.22838400	-0.82356300
H	-10.50961400	-4.61513900	-2.29330800
H	-11.12962200	-6.01283500	-3.17795900
H	-11.79466100	-5.60008700	-1.60118600
H	-9.60338100	-8.69496800	-1.06444800
H	-10.56377200	-8.34868400	-2.49879800
H	-11.24885500	-8.08739000	-0.90136800
H	-11.55900300	5.78874900	-0.25935800
H	-7.80663600	5.78767900	-2.07341700
H	-8.11482500	5.85605800	2.08956200
H	-10.71971900	4.85672400	-2.41672100
H	-11.98002700	6.06399000	-2.67721000
H	-10.29416500	6.47898100	-2.96184800
H	-11.29840100	8.17914300	0.33351900
H	-12.25589400	8.01080600	-1.13293100
H	-10.57783500	8.52509700	-1.23533500
H	-6.34151800	4.90605400	-0.26575200
H	-5.50761800	6.12337400	-1.23569600
H	-6.11191500	6.53998200	0.36334400
H	-8.50059200	8.16478700	-2.17535100
H	-6.74967900	8.02723500	-2.27955500
H	-7.50031200	8.55080100	-0.77852200
H	-10.40385300	4.95102800	2.47657500
H	-9.99687000	6.20227400	3.65123800
H	-11.08394400	6.57139100	2.31802600
H	-7.71272900	8.22281600	1.48601600
H	-8.50911700	8.11647000	3.05111300
H	-9.42923700	8.58843900	1.62909100

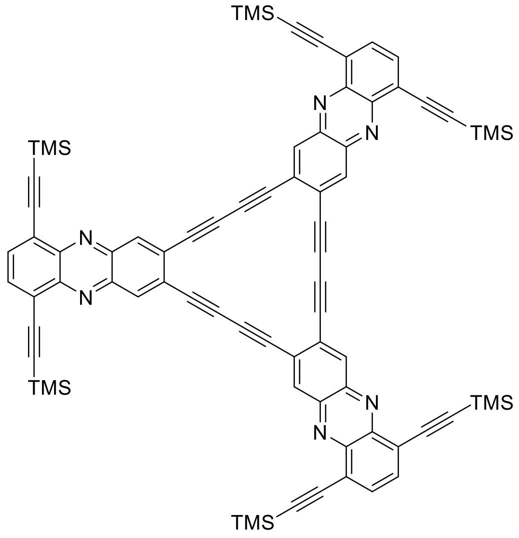

3b$_3$

C	11.37522400	0.70522000	-0.02161500
C	11.20239700	2.10154500	-0.02186400
C	9.95177000	2.68408900	-0.01624500
C	8.79605700	1.82262700	-0.01020600
C	8.97355100	0.38921100	-0.01046900
C	10.30438200	-0.16459200	-0.01611500
N	7.57935900	2.36319800	-0.00474200
C	6.53022600	1.53802500	-0.00008400
C	6.70678600	0.11277500	-0.00053300
N	7.92540100	-0.43168000	-0.00537300
C	5.21688500	2.07158200	0.00477300
C	4.11407800	1.24958900	0.00782000
C	4.29339900	-0.19691500	0.00641500
C	5.56330100	-0.72490000	0.00323900
C	3.16488900	-1.04652900	0.00752800
C	2.19839400	-1.78122300	0.00751600
C	2.81255300	1.79854100	0.01074500
C	1.69552200	2.27409800	0.01208700
C	0.45024900	2.80105100	0.01281700
C	-0.66883100	3.27184400	0.01288600
C	-2.80344500	0.33354200	0.00415600
C	-2.95246800	1.53839000	0.00479400
C	-3.13147600	2.93962700	0.00626700
C	-1.97043900	3.82062700	0.01255400
C	-2.15133700	5.18407300	0.01576500
C	-3.44981300	5.75272800	0.01172600
C	-4.59389800	4.88463700	0.00312600
C	-4.39587200	3.48096500	0.00140800
N	-3.59060600	7.08003300	0.01493200
C	-4.82677100	7.57442000	0.00833600
C	-5.97746200	6.70135900	-0.00268000
N	-5.83423300	5.37768700	-0.00414500
C	-5.01607800	9.00337800	0.01075100
C	-6.30583700	9.49292900	-0.00017200
C	-7.42660800	8.64260300	-0.01246300
C	-7.30261600	7.26849200	-0.01335900
C	-5.07624100	-10.19910700	-0.00371200
C	-3.78076800	-10.74784600	-0.00085600
C	-2.65097000	-9.95595500	0.00206200

C	-2.81910600	-8.52443200	0.00091600
C	-4.14915100	-7.96099400	-0.00256100
C	-5.29399300	-8.83666100	-0.00465700
N	-1.74241700	-7.74122100	0.00353500
C	-1.93220900	-6.42002800	0.00232300
C	-3.25464900	-5.85976600	-0.00105900
N	-4.33569700	-6.64262400	-0.00325300
C	-0.81334000	-5.54969200	0.00464600
C	-0.97323900	-4.18356900	0.00388200
C	-2.31518600	-3.61509400	0.00146800
C	-3.40772200	-4.45048500	-0.00110600
C	-2.48584300	-2.21288200	0.00278800
C	-2.63654800	-1.00827900	0.00384500
C	0.15357000	-3.33167400	0.00591400
C	1.12204600	-2.59964700	0.00706600
C	10.50285300	-1.56838500	-0.01573800
C	10.70547800	-2.76464500	-0.01513700
C	9.80206400	4.09387100	-0.01570600
C	9.70646900	5.30345400	-0.01505200
C	-3.90217800	9.88026100	0.02290000
C	-2.96945900	10.65617800	0.03235700
C	-8.44702500	6.43173100	-0.02749700
C	-9.44512400	5.74204200	-0.04016200
C	-6.60936900	-8.30788800	-0.00458500
C	-7.74776300	-7.88829700	-0.00325900
C	-1.35519200	-10.53114100	0.00594700
C	-0.25999500	-11.05337000	0.01116600
Si	10.98194700	-4.58474900	-0.01667300
C	12.40980000	-4.96993600	1.14199000
C	9.40741300	-5.42085700	0.57190000
C	11.39729900	-5.11711000	-1.76994200
Si	9.51704500	7.13471500	-0.01632500
C	7.74117800	7.54480800	0.43373600
C	10.70462600	7.85470100	1.24913000
C	9.92790400	7.77015200	-1.73602300
Si	-1.52908800	11.80273100	0.04973600
C	-0.80451200	11.80929300	1.78284300
C	-0.26458000	11.18198900	-1.19206100
C	-2.12798800	13.52005600	-0.42073700
Si	-10.93662300	4.66304900	-0.05076500
C	-11.27771600	4.10985500	1.71177500
C	-12.38446400	5.65549300	-0.72019900
C	-10.58447800	3.18179100	-1.14980100
Si	-9.46082600	-7.21375700	0.00049200
C	-9.85670900	-6.62124600	1.73853700
C	-9.53917200	-5.78760700	-1.21825400
C	-10.63878600	-8.58545300	-0.51058300
Si	1.41831500	-11.81000000	0.02782000
C	2.07207500	-11.75557300	1.78811600
C	1.27673000	-13.58649700	-0.56662300
C	2.52457900	-10.81621500	-1.11874600
H	12.37790100	0.29949800	-0.02566900
H	12.07596800	2.73941400	-0.02604700
H	5.10411300	3.14681600	0.00557800
H	5.71605000	-1.79518300	0.00288800
H	-1.30239900	5.85346100	0.02049500
H	-5.26906800	2.84352100	-0.00460300
H	-6.45818800	10.56380500	0.00034300
H	-8.41674500	9.07791800	-0.02184100
H	-5.92909200	-10.86440400	-0.00512700

H	-3.66501100	-11.82328700	-0.00044300
H	0.17408200	-5.98984700	0.00709100
H	-4.41079100	-4.04718600	-0.00305100
H	12.60656900	-6.04490700	1.16958800
H	13.32553900	-4.46967000	0.81990600
H	12.19057400	-4.64268500	2.16044200
H	9.52496800	-6.50768900	0.58033500
H	8.56704700	-5.17757200	-0.08132000
H	9.14658800	-5.10227800	1.58318200
H	10.58395600	-4.87606900	-2.45741100
H	12.29906800	-4.61826900	-2.13108800
H	11.56855600	-6.19588700	-1.81599500
H	7.58285300	8.62641300	0.44321400
H	7.04409100	7.10806200	-0.28422800
H	7.48680900	7.15814700	1.42266400
H	10.62366000	8.94447700	1.27958200
H	11.73932200	7.60204600	1.00801400
H	10.49216900	7.47423500	2.25037800
H	9.25994400	7.33955200	-2.48480100
H	9.82666500	8.85780200	-1.78044500
H	10.95232000	7.51660600	-2.01658600
H	0.06015100	12.47595100	1.83859400
H	-1.53848700	12.14865000	2.51665400
H	-0.47814800	10.80913500	2.07497400
H	0.61421200	11.83209700	-1.20748100
H	-0.68196700	11.15653200	-2.20074100
H	0.06734500	10.17215200	-0.94285300
H	-2.57539300	13.52242200	-1.41690700
H	-2.87867700	13.88383400	0.28407100
H	-1.29835700	14.23187900	-0.42391500
H	-12.15728100	3.46165700	1.74969000
H	-10.43156100	3.55330700	2.11977500
H	-11.46177500	4.96471500	2.36577000
H	-13.29257100	5.04739400	-0.74439800
H	-12.18743300	6.00430100	-1.73609600
H	-12.58399900	6.53105000	-0.09877200
H	-9.72503800	2.61709900	-0.78289700
H	-11.44442300	2.50743700	-1.17844900
H	-10.36654300	3.49302800	-2.17346700
H	-10.86778000	-6.20805100	1.78531500
H	-9.79465400	-7.44020200	2.45813200
H	-9.15992400	-5.84329800	2.05709200
H	-10.54226000	-5.35360700	-1.24232600
H	-9.29158600	-6.11878500	-2.22884100
H	-8.83654900	-4.99795800	-0.94465300
H	-10.58391100	-9.42976200	0.17989400
H	-11.67139900	-8.22678700	-0.52021400
H	-10.40314000	-8.95552000	-1.51061800
H	3.07250200	-12.19305500	1.84255500
H	2.13344900	-10.72828900	2.15293300
H	1.42409300	-12.31309800	2.46757500
H	2.25571500	-14.07288900	-0.57064400
H	0.87571700	-13.63196600	-1.58133300
H	0.61648900	-14.16799300	0.08035200
H	2.14207300	-10.82495300	-2.14141100
H	2.58928700	-9.77521400	-0.79590200
H	3.53692800	-11.22857800	-1.13410500

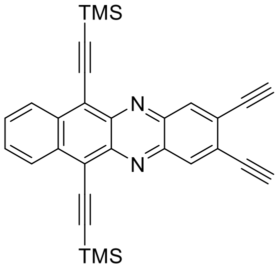

3c

C	0.72116400	-2.56804100	-0.00347900
C	-0.72119700	-2.56803000	-0.00346900
C	-1.43626200	-1.35304100	-0.00237900
C	-0.72261300	-0.12169600	-0.00178800
C	0.72261500	-0.12170600	-0.00179500
C	1.43624700	-1.35306100	-0.00239500
N	-1.40909900	1.03054600	-0.00134200
C	-0.72108000	2.16451700	-0.00123700
C	0.72111400	2.16450700	-0.00124400
N	1.40911700	1.03052700	-0.00135600
C	-1.40943800	3.40917200	-0.00119100
C	-0.72877500	4.59795700	-0.00112200
C	0.72884400	4.59794700	-0.00113000
C	1.40949000	3.40915200	-0.00120700
C	2.85160100	-1.33673900	-0.00173600
C	4.06595000	-1.34283800	-0.00090800
C	-2.85161700	-1.33669900	-0.00170100
C	-4.06596500	-1.34278500	-0.00086400
C	1.44074900	5.82913500	-0.00116700
C	2.05224800	6.86414500	-0.00121800
C	-1.44066200	5.82915600	-0.00115100
C	-2.05216000	6.86416700	-0.00119100
C	1.39869500	-3.82321600	-0.00464900
C	0.70994500	-4.99815100	-0.00578500
C	-0.71001200	-4.99814100	-0.00577400
C	-1.39874600	-3.82319600	-0.00462800
Si	5.90532400	-1.29232200	0.00315400
C	6.53345300	-2.33213100	-1.43065300
C	6.51566200	-1.99492300	1.63571800
C	6.44786400	0.49441600	-0.18998800
Si	-5.90534000	-1.29227700	0.00315000
C	-6.53338800	-2.33086600	-1.43157600
C	-6.44788200	0.49462100	-0.18849900
C	-6.51575800	-1.99627300	1.63508300
H	-2.49034300	3.39070800	-0.00143700
H	2.49039500	3.39067300	-0.00146600
H	2.58796200	7.78155800	-0.00138700
H	-2.58817200	7.78140600	-0.00126600
H	2.47956500	-3.81800400	-0.00470400
H	1.24481400	-5.93941600	-0.00674500
H	-1.24489400	-5.93939900	-0.00672500
H	-2.47961600	-3.81796900	-0.00466600
H	6.16512500	-1.95071000	-2.38514000
H	7.62610500	-2.32375700	-1.46432100
H	6.20938300	-3.37090200	-1.33864800
H	6.13790600	-1.41441900	2.47979400
H	7.60793400	-1.97952600	1.67859500
H	6.18944000	-3.02851300	1.76879000

H	7.53829400	0.57127000	-0.19080700
H	6.07902200	0.91780700	-1.12632200
H	6.06670300	1.11015800	0.62706000
H	-6.20929200	-3.36970700	-1.34045600
H	-7.62603800	-2.32249100	-1.46528000
H	-6.16503100	-1.94861200	-2.38571900
H	-6.06679400	1.10967100	0.62910400
H	-7.53831300	0.57146300	-0.18933900
H	-6.07897000	0.91881500	-1.12444200
H	-7.60803200	-1.98092000	1.67791800
H	-6.18953700	-3.02997500	1.76729100
H	-6.13804900	-1.41648600	2.47967300

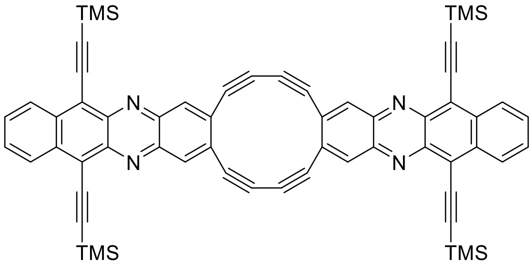

3c$_2$

C	10.28572400	-0.72086300	-0.00001300
C	10.28573000	0.72076500	-0.00002300
C	9.06961300	1.43612100	-0.00001500
C	7.83991600	0.72244000	0.00000000
C	7.83991000	-0.72251400	0.00000900
C	9.06960000	-1.43620800	0.00000500
N	6.68587400	1.40866700	0.00000400
C	5.55274100	0.72208900	0.00001400
C	5.55273500	-0.72214500	0.00002200
N	6.68586200	-1.40873200	0.00002100
C	4.30948400	1.41582900	0.00001500
C	3.12015300	0.73843700	0.00002100
C	3.12014700	-0.73847400	0.00002700
C	4.30947400	-1.41587500	0.00002900
C	1.86109100	-1.38073600	0.00003700
C	0.67653800	-1.65339000	0.00004000
C	1.86110200	1.38070900	0.00001500
C	0.67655200	1.65337400	0.00001100
C	-0.67657400	1.65339200	0.00001700
C	-1.86112900	1.38074700	0.00002400
C	-0.67658700	-1.65337200	0.00002500
C	-1.86113400	-1.38069500	0.00001100
C	-3.12018800	-0.73842700	-0.00001300
C	-3.12018500	0.73848300	-0.00001200
C	-4.30951200	1.41588300	-0.00002900
C	-5.55277300	0.72215000	-0.00004600
C	-5.55277500	-0.72208400	-0.00004400
C	-4.30951800	-1.41582200	-0.00002800
N	-6.68590100	1.40873400	-0.00005900
C	-7.83994700	0.72251400	-0.00006600
C	-7.83995000	-0.72244100	-0.00005800
N	-6.68590600	-1.40866500	-0.00004800
C	-9.06964000	1.43620300	-0.00007100
C	-10.28576100	0.72085500	-0.00009000
C	-10.28576400	-0.72077200	-0.00008200
C	-9.06964500	-1.43612500	-0.00005400
C	11.53999300	-1.39872200	-0.00001700

C	12.71537100	-0.70974900	-0.00003400
C	12.71537700	0.70963100	-0.00004700
C	11.54000500	1.39861300	-0.00004100
C	-11.54003200	1.39871200	-0.00012200
C	-12.71540900	0.70973700	-0.00014600
C	-12.71541100	-0.70964300	-0.00014000
C	-11.54003800	-1.39862300	-0.00010700
C	-9.05443600	-2.85140300	-0.00003300
C	-9.06382300	-4.06567500	-0.00001600
C	-9.05442500	2.85148200	-0.00006100
C	-9.06380200	4.06575300	-0.00003700
C	9.05438100	-2.85148600	0.00002100
C	9.05440700	2.85139900	-0.00002300
C	9.06382600	4.06567100	-0.00003100
C	9.06374100	-4.06575800	0.00003700
Si	-9.01434900	5.90555400	0.00012300
C	-8.10667200	6.47199700	-1.54343600
C	-10.77929700	6.55054200	-0.00049500
C	-8.10785100	6.47176400	1.54446000
Si	-9.01438100	-5.90547600	0.00003000
C	-10.77933200	-6.55046200	0.00066000
C	-8.10783400	-6.47186000	-1.54421400
C	-8.10677000	-6.47175000	1.54368800
Si	9.01417200	-5.90555600	0.00004400
C	10.77908100	-6.55065300	0.00142500
C	8.10586000	-6.47178800	1.54330400
C	8.10826300	-6.47186800	-1.54460200
Si	9.01465400	5.90547900	-0.00004500
C	8.10755200	6.47195300	1.54383900
C	10.77970200	6.55020000	0.00007900
C	8.10777600	6.47193600	-1.54406700
H	4.33262900	2.49681900	0.00001100
H	4.33261100	-2.49686600	0.00004000
H	-4.33265100	2.49687300	-0.00002100
H	-4.33266000	-2.49681200	-0.00002400
H	11.53487500	-2.47962000	-0.00000600
H	13.65651300	-1.24479500	-0.00003600
H	13.65652400	1.24466800	-0.00006200
H	11.53489600	2.47951100	-0.00005000
H	-11.53491600	2.47961000	-0.00012600
H	-13.65655200	1.24478000	-0.00017200
H	-13.65655600	-1.24468200	-0.00016400
H	-11.53492800	-2.47952100	-0.00010300
H	-8.04637300	7.56296500	-1.57851800
H	-7.08907000	6.07715600	-1.56697300
H	-8.61654200	6.13393700	-2.44782400
H	-10.79163800	7.64357600	-0.00099400
H	-11.32476500	6.21089000	0.88251700
H	-11.32443600	6.21008800	-0.88340000
H	-7.09022200	6.07703100	1.56866000
H	-8.04769400	7.56273200	1.57982200
H	-8.61835000	6.13345500	2.44840100
H	-10.79165800	-7.64349800	0.00114400
H	-11.32446800	-6.21002900	0.88357400
H	-11.32481300	-6.21081000	-0.88234300
H	-8.04786900	-7.56283800	-1.57956800
H	-7.09013200	-6.07731000	-1.56831200
H	-8.61818000	-6.13345700	-2.44820500
H	-8.61691000	-6.13393300	2.44801500
H	-8.04610900	-7.56270000	1.57869400

H	-7.08930600	-6.07656700	1.56743200
H	10.79135100	-7.64368900	0.00156600
H	11.32505800	-6.21074400	-0.88117200
H	11.32375700	-6.21052700	0.88474200
H	8.04539300	-7.56274600	1.57842800
H	7.08830700	-6.07680100	1.56646700
H	8.61546600	-6.13374900	2.44784900
H	8.61915500	-6.13369000	-2.44837000
H	8.04804900	-7.56283500	-1.57987200
H	7.09067100	-6.07707000	-1.56926200
H	8.04744800	7.56292900	1.57902900
H	7.08989000	6.07728400	1.56767500
H	8.61765900	6.13373400	2.44803500
H	10.79220600	7.64323300	0.00007500
H	11.32501900	6.21006100	-0.88283700
H	11.32489100	6.21006900	0.88307800
H	7.09012300	6.07725500	-1.56805400
H	8.04766500	7.56291100	-1.57926900
H	8.61802200	6.13372100	-2.44818600

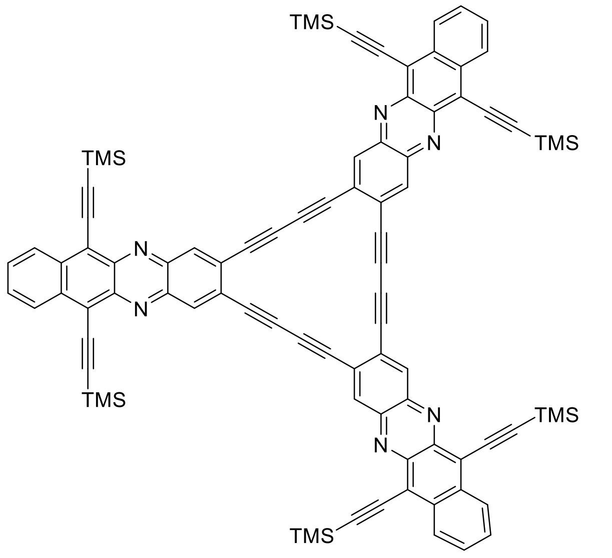

3c$_3$

C	11.16885700	2.41546000	0.00472000
C	10.77614800	3.80296500	0.00559700
C	9.41179800	4.16037100	0.00417700
C	8.42172100	3.13885900	0.00202400
C	8.81556000	1.74755600	0.00111500
C	10.19410200	1.39622100	0.00230500
N	7.12575800	3.48541200	0.00088500
C	6.22200200	2.51473900	-0.00049300
C	6.61512700	1.12606900	-0.00101800
N	7.89354300	0.77318300	-0.00041000
C	4.83835800	2.83939000	-0.00135100
C	3.87589000	1.86054400	-0.00222600
C	4.27437300	0.45320100	-0.00251800
C	5.60712400	0.12425900	-0.00210400
C	3.28750400	-0.55636700	-0.00317500
C	2.44172400	-1.42782600	-0.00360100
C	2.50614300	2.20241600	-0.00286300
C	1.32884700	2.50026800	-0.00328500
C	0.01818900	2.83067400	-0.00314700
C	-1.15927100	3.12779800	-0.00294900

C	-2.82484700	-0.09792900	-0.00200100
C	-3.15603400	1.07043100	-0.00131400
C	-3.54579200	2.42733100	-0.00126800
C	-2.52719500	3.47692800	-0.00210000
C	-2.90981600	4.79529800	-0.00235000
C	-4.28180000	5.16616000	-0.00167100
C	-5.28687800	4.13042200	-0.00055800
C	-4.87504000	2.77023700	-0.00046500
N	-4.61666700	6.44942500	-0.00216900
C	-5.92186500	6.75941700	-0.00150100
C	-6.92882100	5.72169600	-0.00017000
N	-6.57966800	4.42646300	0.00023200
C	-6.30851400	8.12845500	-0.00201100
C	-7.67901800	8.46145600	-0.00094300
C	-8.68314200	7.42657900	0.00044900
C	-8.30895900	6.06682200	0.00068000
C	-3.49070600	-10.87843800	0.00197400
C	-2.09263400	-11.23181300	0.00035800
C	-1.10124600	-10.22867800	-0.00134300
C	-1.49132800	-8.86058200	-0.00145400
C	-2.89323700	-8.50629500	-0.00003300
C	-3.88643100	-9.52472700	0.00171800
N	-0.54345500	-7.91131800	-0.00288000
C	-0.93239700	-6.64338400	-0.00274600
C	-2.33167700	-6.28980700	-0.00140300
N	-3.27631700	-7.22067700	-0.00013600
C	0.04038900	-5.60730800	-0.00366200
C	-0.32637700	-4.28444200	-0.00328000
C	-1.74446700	-3.92616400	-0.00231400
C	-2.69554300	-4.91600800	-0.00137900
C	-2.12517500	-2.56670700	-0.00215400
C	-2.45636400	-1.39838200	-0.00210000
C	0.65427300	-3.26887600	-0.00369800
C	1.49999100	-2.39742600	-0.00376700
C	-4.45523100	-11.92869300	0.00367900
C	-4.07527900	-13.23683300	0.00378700
C	-2.69889900	-13.58476700	0.00219800
C	-1.74297500	-12.61429100	0.00055300
C	12.56061400	2.10489800	0.00629000
C	13.50374100	3.08780300	0.00868800
C	13.11714200	4.45381700	0.00969700
C	11.79878700	4.79675200	0.00820100
C	-8.10753500	9.82153600	-0.00122400
C	-9.43073100	10.14531900	-0.00020000
C	-10.41929800	9.12642700	0.00120400
C	-10.05557800	7.81367100	0.00154300
C	10.56431900	0.03023900	0.00178300
C	10.90142000	-1.13633700	0.00134600
C	9.01186300	5.51794700	0.00517600
C	8.68975200	6.68877800	0.00592400
C	-5.31229000	9.13365200	-0.00332800
C	-4.47334300	10.01157300	-0.00480100
C	-9.28342000	5.04050200	0.00237700
C	-10.13492200	4.17476500	0.00375400
C	-5.25476300	-9.16321200	0.00332300
C	-6.43410500	-8.87380700	0.00480900
C	0.27456500	-10.56060300	-0.00304300
C	1.44985600	-10.86602200	-0.00465200
Si	11.34731500	-2.92178000	-0.00077500
C	13.22058300	-3.07037700	0.00197900

C	10.62262600	-3.71301800	1.54069500
C	10.62752800	-3.70801100	-1.54710600
Si	8.13879000	8.44469600	0.00430500
C	7.09558200	8.73815100	1.53822000
C	9.65978100	9.54819100	0.02226900
C	7.12738700	8.74472600	-1.54958100
Si	-3.15469900	11.29530300	-0.00694900
C	-2.10561900	11.06185700	-1.54711400
C	-3.96972400	12.98860800	-0.01654700
C	-2.11362700	11.07536900	1.54066300
Si	-11.37683100	2.81664500	0.00355700
C	-13.09509600	3.57744800	0.00020900
C	-11.11526400	1.77725700	-1.53874600
C	-11.11934600	1.78121500	1.54921900
Si	-8.20461600	-8.37183700	0.00762600
C	-8.53469500	-7.36186800	-1.54103000
C	-9.26532300	-9.92307700	0.01989400
C	-8.52483600	-7.34429700	1.54675400
Si	3.24636100	-11.26493000	-0.00757700
C	4.02050900	-10.51354500	1.52975100
C	3.44344800	-13.13374400	0.00296000
C	4.01159200	-10.53195800	-1.55821600
H	4.56286800	3.88480500	-0.00115700
H	5.92058700	-0.91040300	-0.00249400
H	-2.17118400	5.58473900	-0.00319300
H	-5.64199500	2.00828900	0.00015600
H	1.08354000	-5.89124100	-0.00447600
H	-3.74836200	-4.67036000	-0.00050500
H	-5.50192000	-11.65889200	0.00484900
H	-4.82458900	-14.01821600	0.00509100
H	-2.41091900	-14.62836600	0.00228000
H	-0.69380100	-12.87425700	-0.00065500
H	12.85006900	1.06344500	0.00550700
H	14.55504200	2.82936000	0.00983600
H	13.87709800	5.22484600	0.01176100
H	11.49956900	5.83542600	0.00903300
H	-7.35123400	10.59380400	-0.00232600
H	-9.73374600	11.18464900	-0.00048100
H	-11.46732100	9.39785800	0.00198600
H	-10.80455800	7.03429600	0.00257500
H	13.52538800	-4.12012000	0.00144700
H	13.65612500	-2.59575900	-0.87987200
H	13.65351700	-2.59747900	0.88603400
H	10.85629900	-4.78043700	1.57415500
H	9.53648100	-3.60465500	1.56318000
H	11.02249000	-3.25247600	2.44632900
H	11.03022600	-3.24426200	-2.44984700
H	10.86136700	-4.77529100	-1.58357500
H	9.54143900	-3.59960900	-1.57264500
H	6.73894900	9.77100700	1.57182600
H	6.22458900	8.08001300	1.55187300
H	7.66951100	8.55061300	2.44795300
H	9.37240200	10.60285400	0.02254700
H	10.28590200	9.37301200	-0.85517800
H	10.26869600	9.36784100	0.91069100
H	6.25698100	8.08654500	-1.58385900
H	6.77134200	9.77766900	-1.58638900
H	7.72005600	8.56090500	-2.44798100
H	-1.30127500	11.80132500	-1.58321900
H	-1.65201100	10.06895700	-1.56584500

H	-2.70478800	11.17182800	-2.45324300
H	-3.21631100	13.78062800	-0.01889500
H	-4.59936600	13.12941400	0.86455800
H	-4.59600700	13.12092200	-0.90135800
H	-1.66015700	10.08266100	1.57030400
H	-1.30942900	11.81508800	1.57456100
H	-2.71745500	11.19318000	2.44270700
H	-13.86277100	2.79927700	-0.00013600
H	-13.25287700	4.20165400	0.88230700
H	-13.25021300	4.19982900	-0.88365200
H	-11.82953000	0.95036800	-1.57379100
H	-10.10886600	1.35466900	-1.56071700
H	-11.24631900	2.37363000	-2.44391300
H	-10.11265000	1.35951900	1.57530100
H	-11.25354600	2.37987500	2.45242600
H	-11.83303800	0.95386600	1.58432000
H	-9.57695500	-7.03371700	-1.57423800
H	-7.90040000	-6.47380700	-1.57263700
H	-8.33673400	-7.94524800	-2.44249700
H	-10.32761700	-9.66530400	0.02185200
H	-9.06747700	-10.53041000	0.90568500
H	-9.07320100	-10.54049900	-0.86016100
H	-8.32109600	-7.91737500	2.45352800
H	-7.89035100	-6.45598100	1.56417900
H	-9.56686700	-7.01572600	1.58289800
H	5.09361600	-10.71954600	1.56285200
H	3.88421100	-9.43045400	1.54788100
H	3.57143600	-10.92135400	2.43759900
H	4.50074000	-13.41126600	0.00096800
H	2.97804900	-13.58535500	-0.87571600
H	2.98412500	-13.57480700	0.89015100
H	3.55757500	-10.95097100	-2.45847000
H	3.87477800	-9.44921900	-1.58879100
H	5.08458300	-10.73798000	-1.59488100

2.2 NMR Spectroscopy

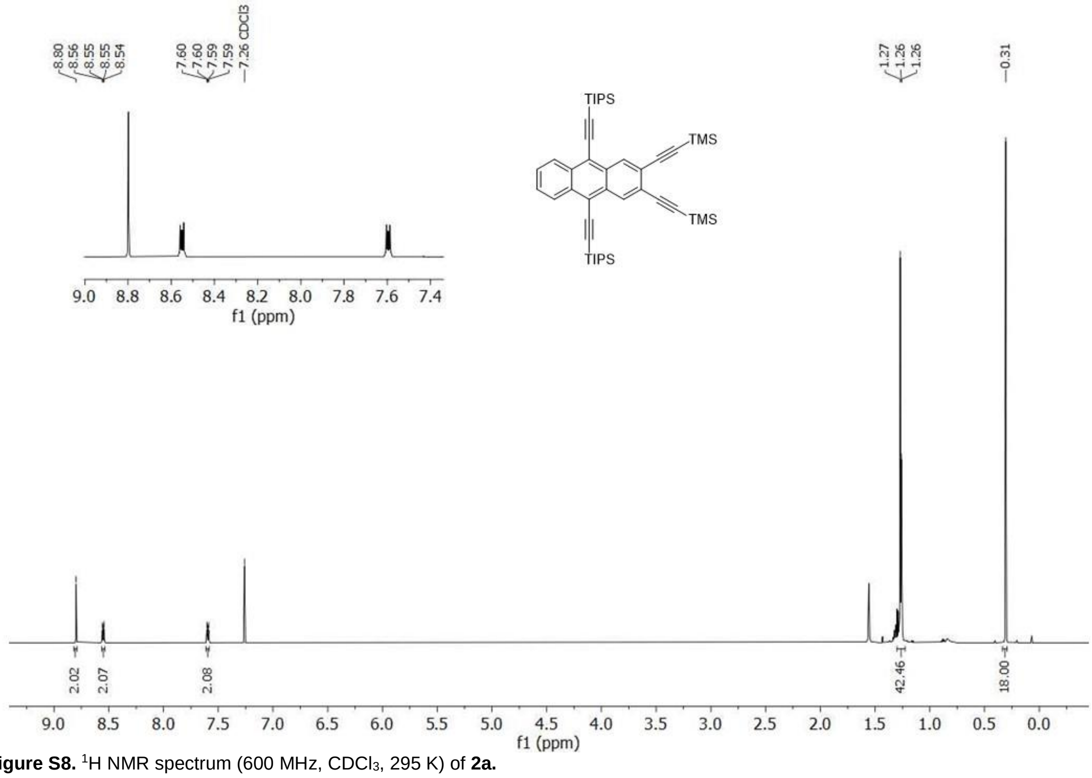

Figure S8. ^{1}H NMR spectrum (600 MHz, $CDCl_3$, 295 K) of **2a.**

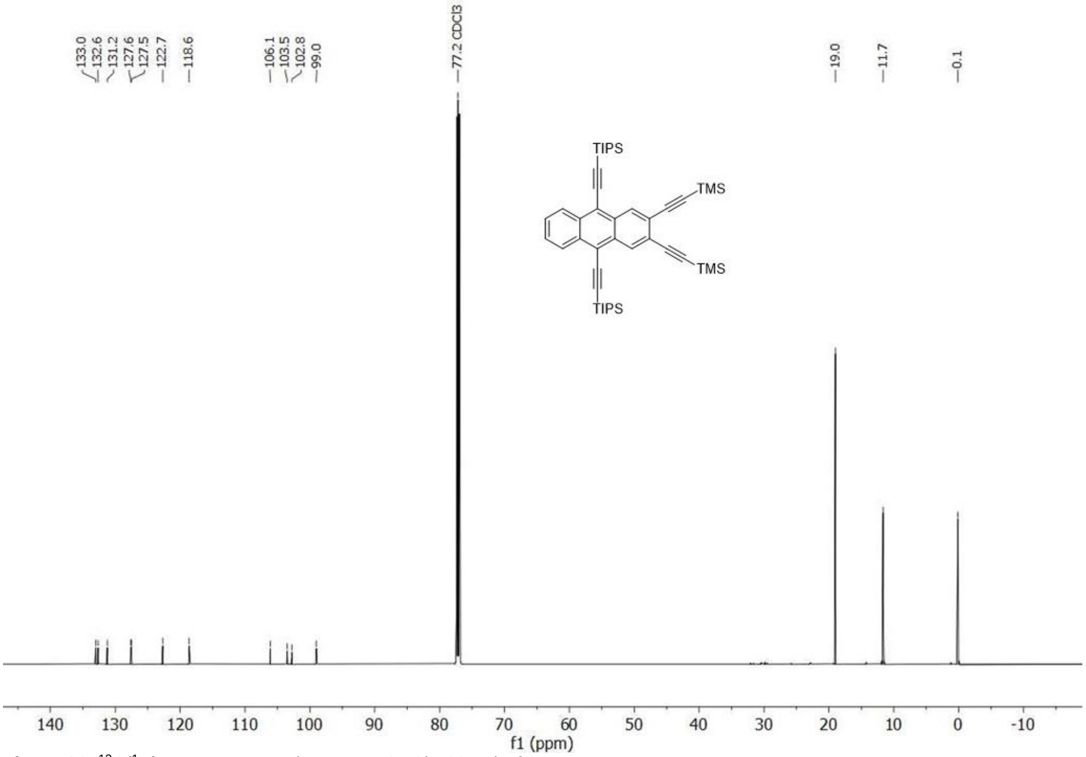

Figure S9. $^{13}C\{^{1}H\}$ NMR spectrum (151 MHz, $CDCl_3$, 295 K) of **2a.**

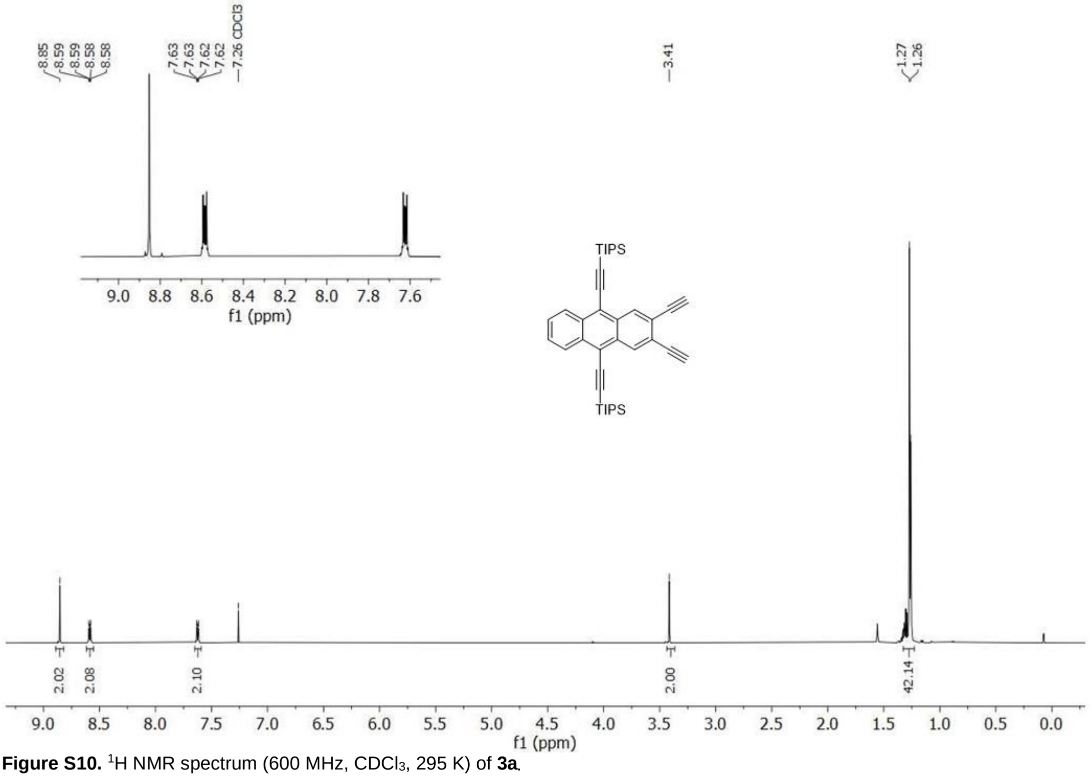

Figure S10. ^{1}H NMR spectrum (600 MHz, $CDCl_3$, 295 K) of **3a**.

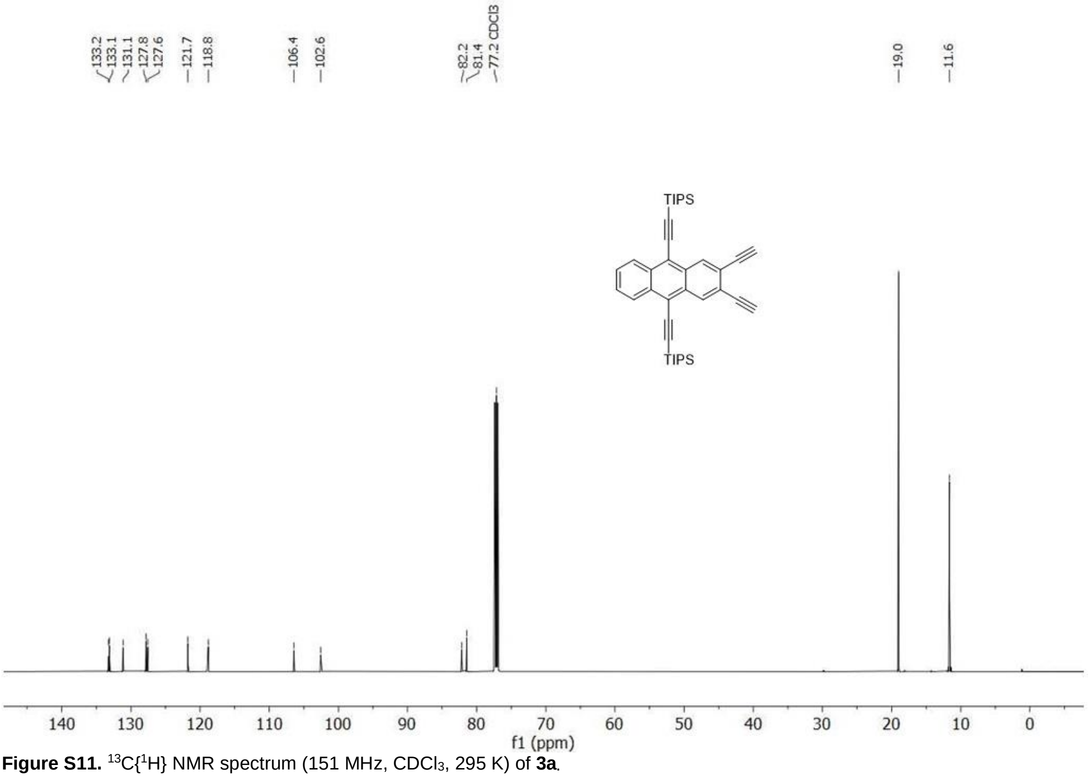

Figure S11. $^{13}C\{^{1}H\}$ NMR spectrum (151 MHz, $CDCl_3$, 295 K) of **3a**.

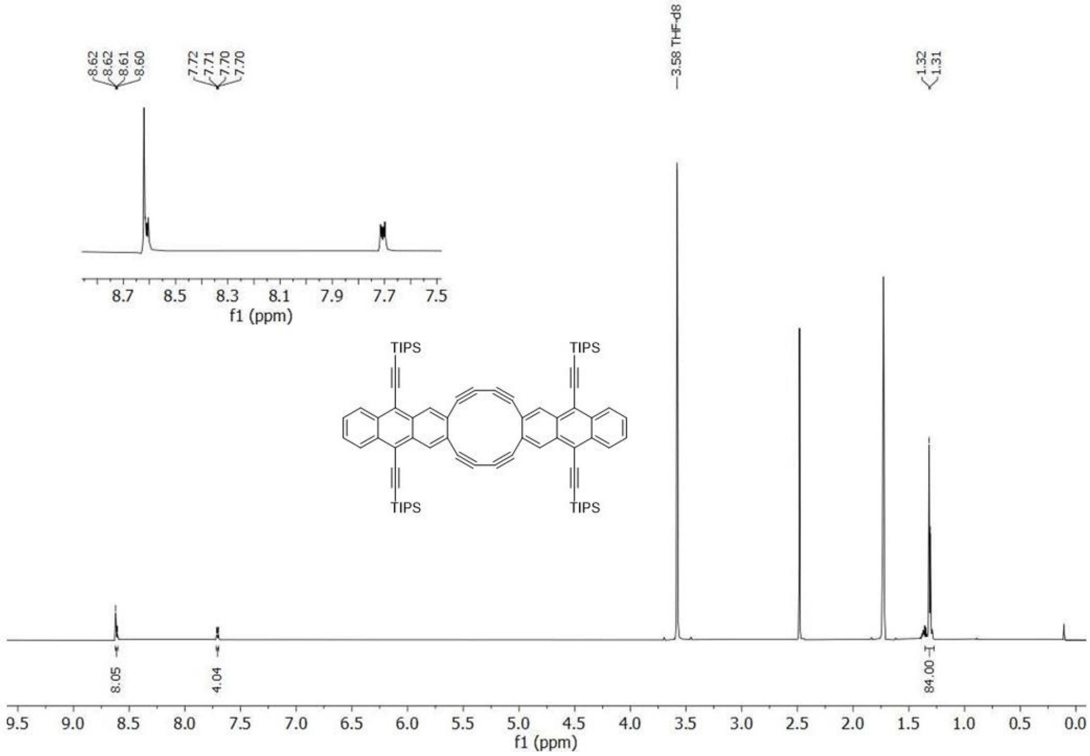

Figure S12. ^{1}H NMR spectrum (600 MHz, THF-d_8, 295 K) of **3a₂**.

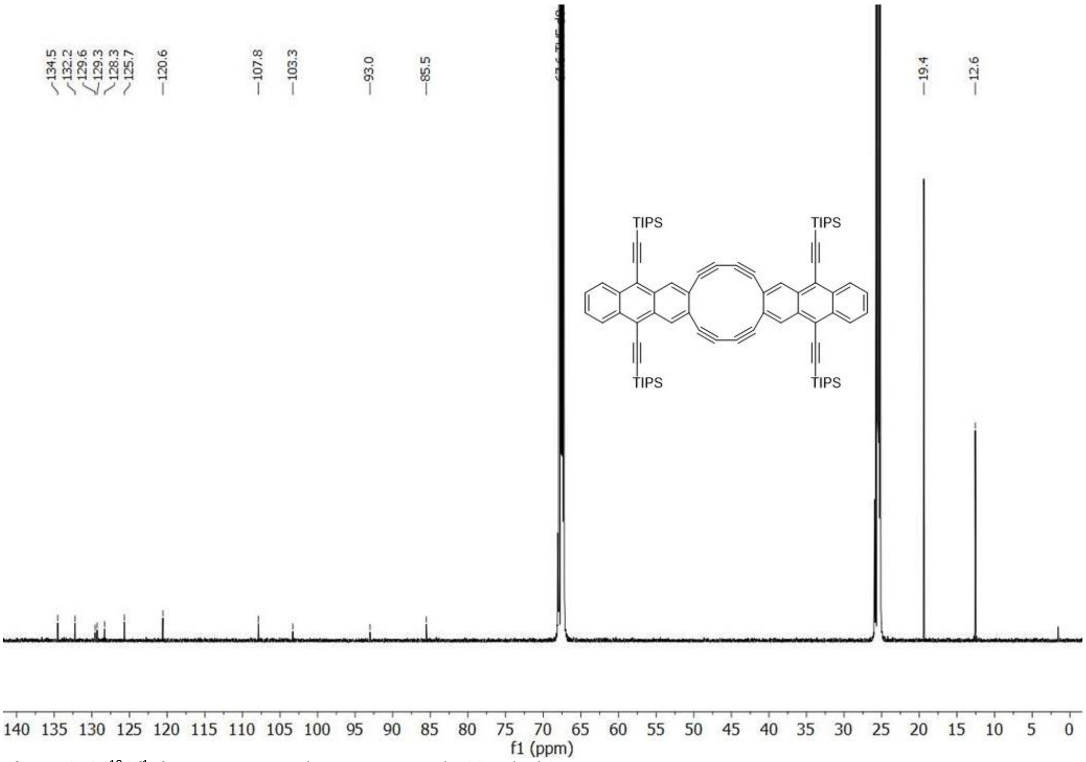

Figure S13. $^{13}C\{^{1}H\}$ NMR spectrum (151 MHz, THF-d_8, 295 K) of **3a₂**.

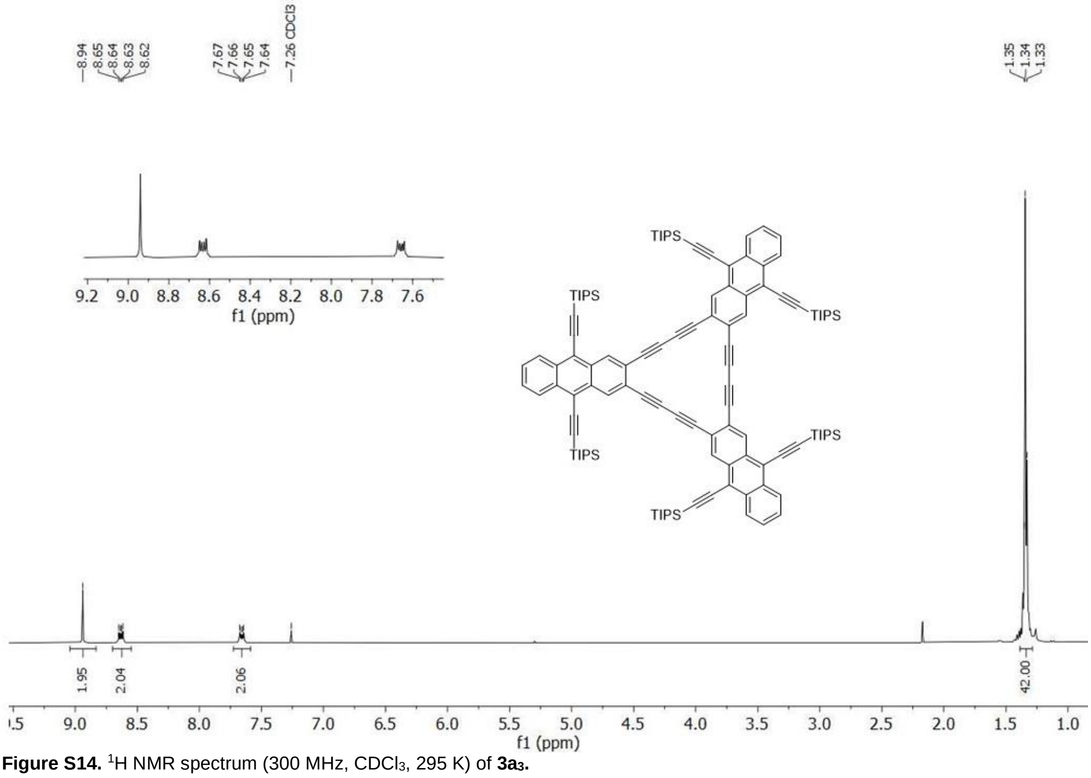

Figure S14. ^{1}H NMR spectrum (300 MHz, $CDCl_3$, 295 K) of **3a$_3$.**

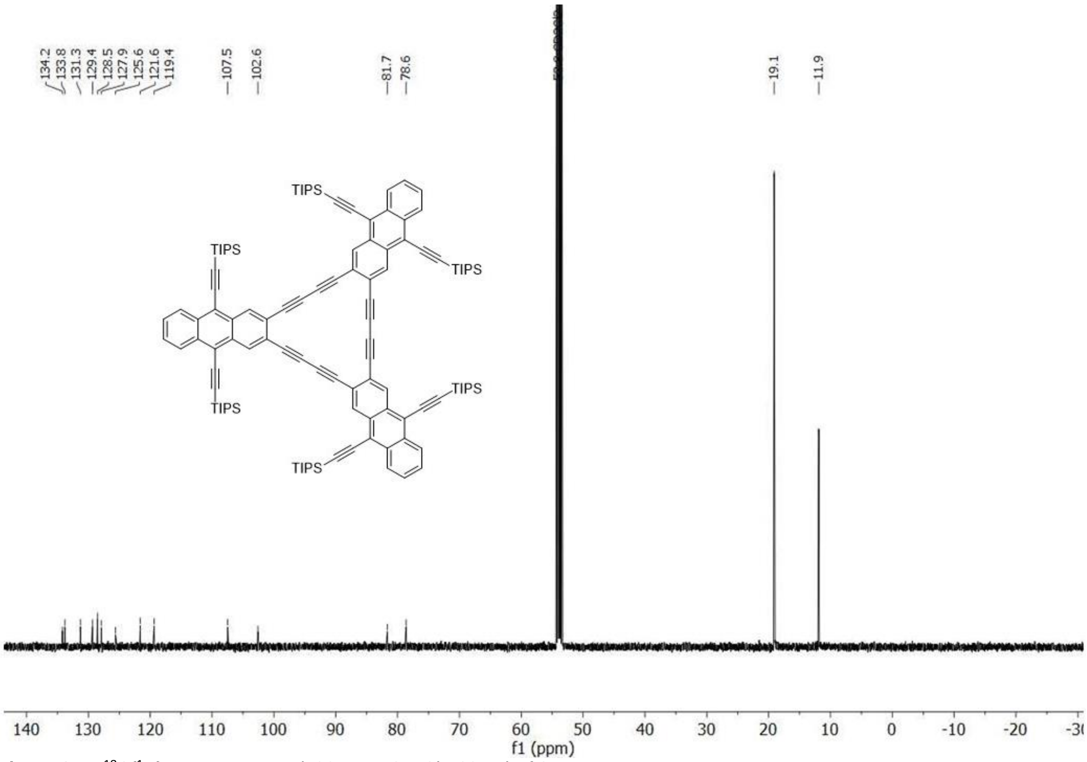

Figure S15. $^{13}C\{^{1}H\}$ NMR spectrum (126 MHz, CD_2Cl_2, 295 K) of **3a$_3$.**

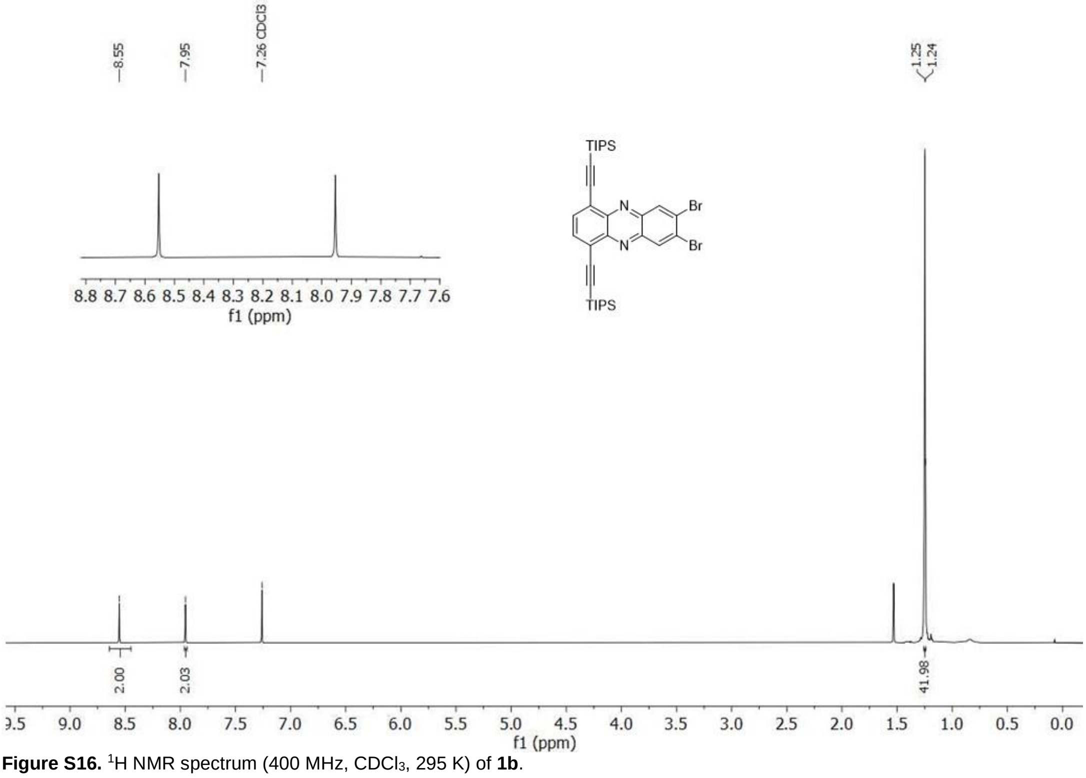

Figure S16. ^{1}H NMR spectrum (400 MHz, $CDCl_3$, 295 K) of **1b**.

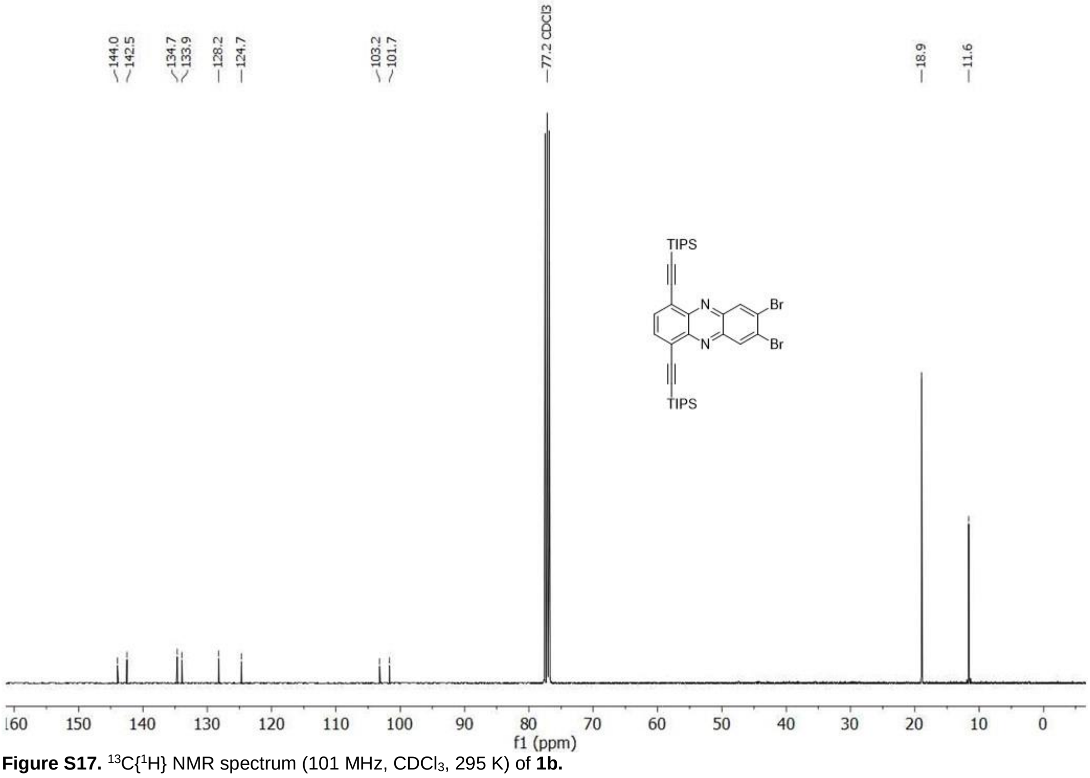

Figure S17. $^{13}C\{^{1}H\}$ NMR spectrum (101 MHz, $CDCl_3$, 295 K) of **1b.**

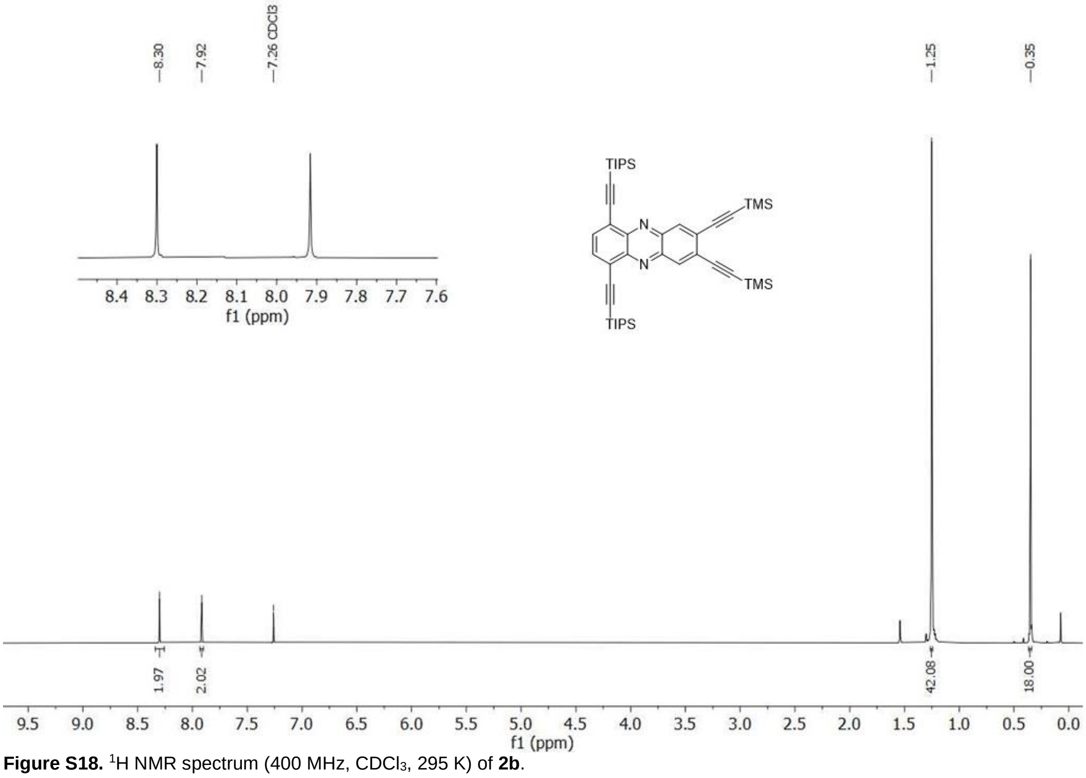

Figure S18. ^{1}H NMR spectrum (400 MHz, $CDCl_3$, 295 K) of **2b**.

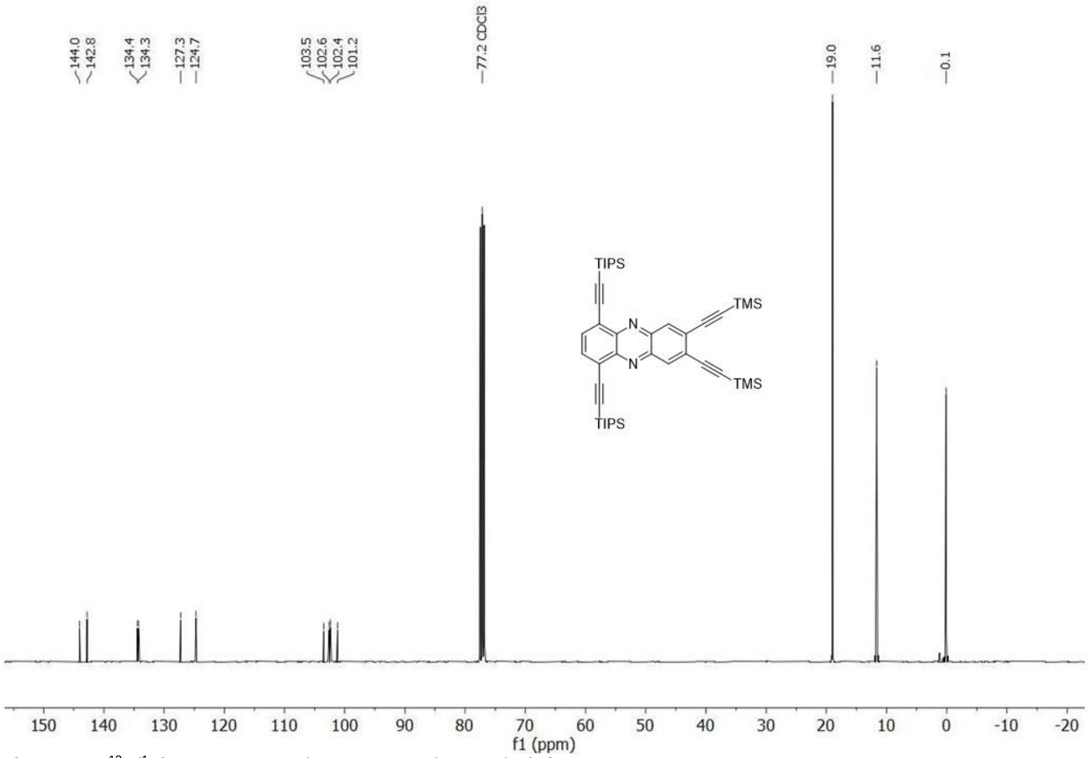

Figure S19. ^{13}C{^{1}H} NMR spectrum (101 MHz, $CDCl_3$, 295 K) of **2b.**

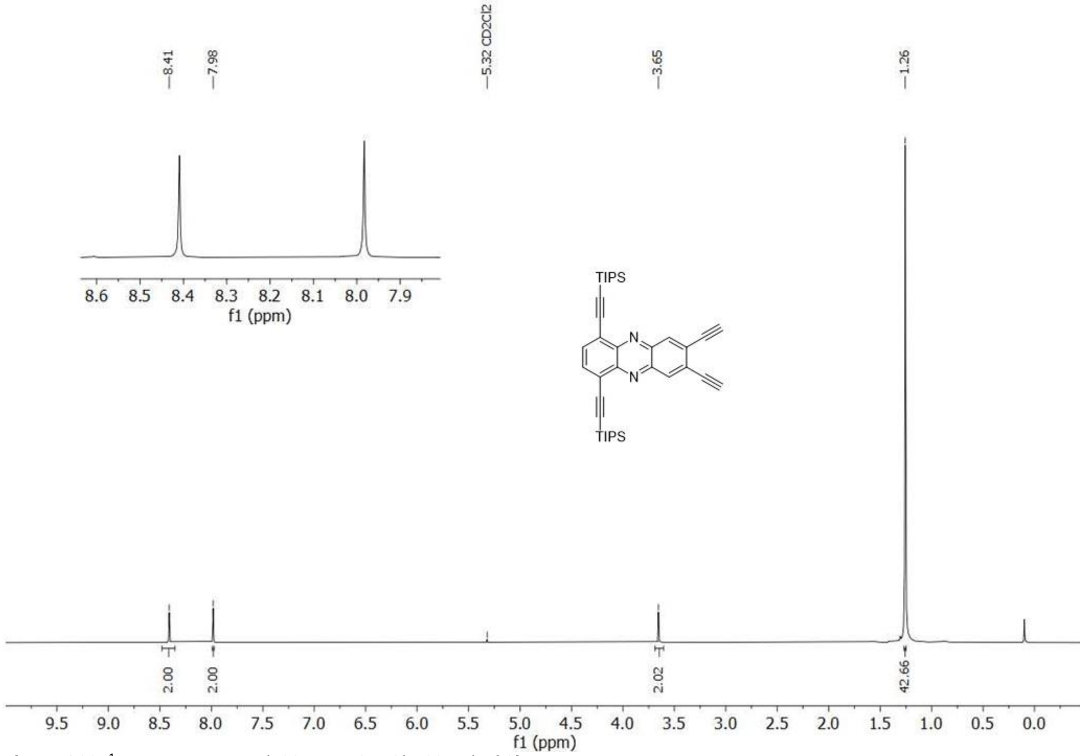

Figure S20. ^{1}H NMR spectrum (400 MHz, CD_2Cl_2, 295 K) of **3b.**

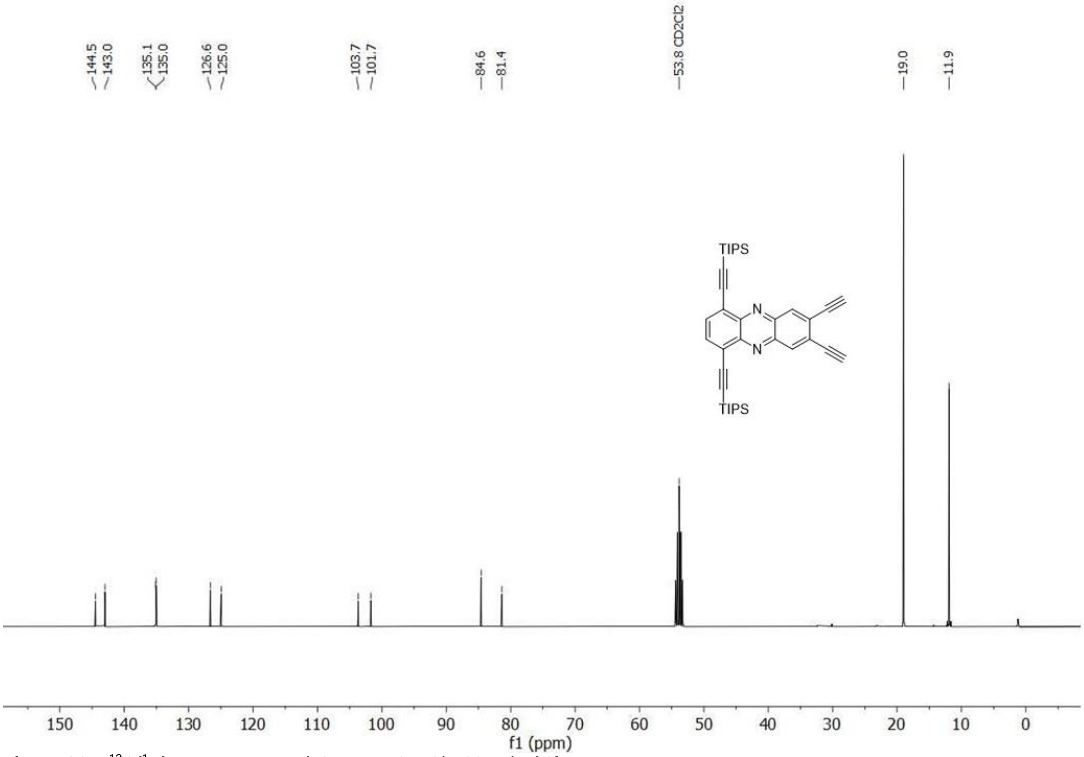

Figure S21. $^{13}C\{^{1}H\}$ NMR spectrum (101 MHz, CD_2Cl_2, 295 K) of **3b.**

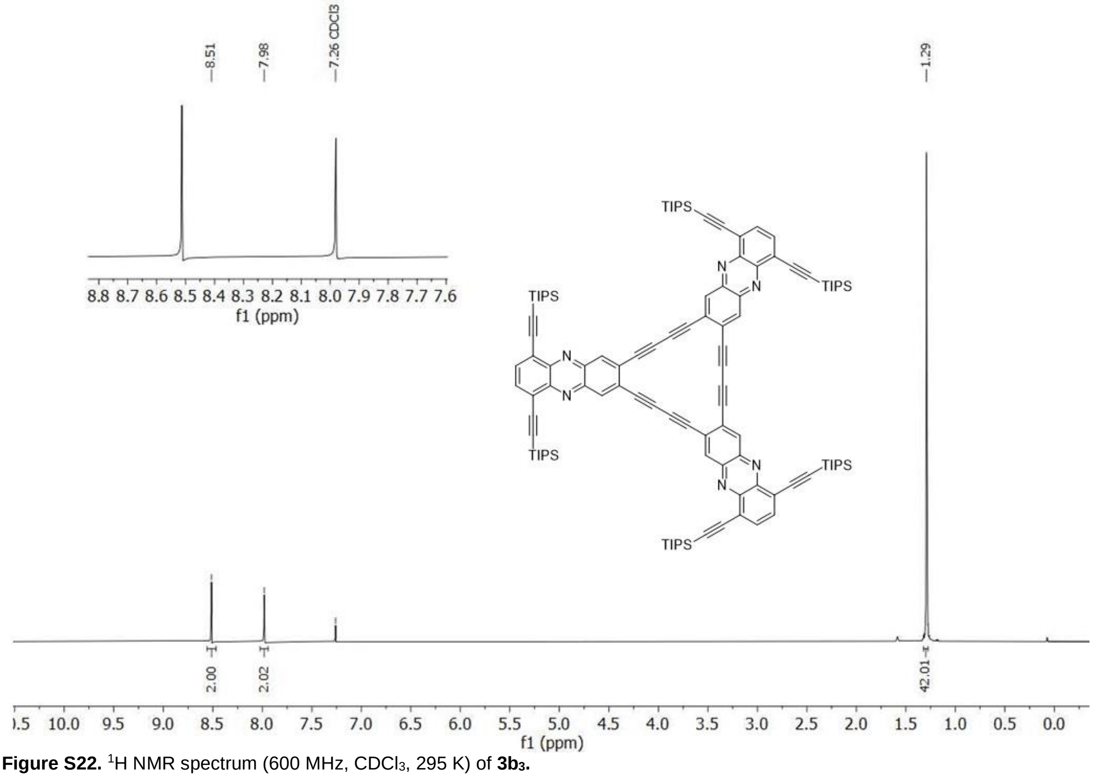

Figure S22. ^{1}H NMR spectrum (600 MHz, $CDCl_3$, 295 K) of **$3b_3$.**

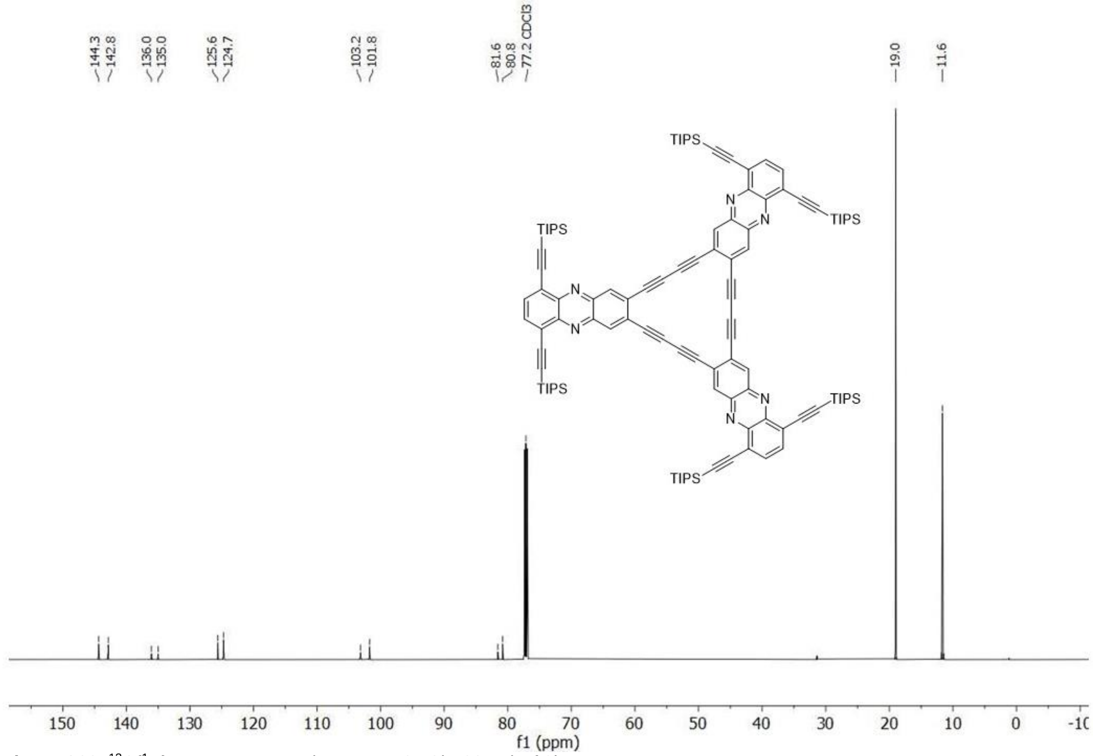

Figure S23. $^{13}C\{^1H\}$ -NMR spectrum (151 MHz, $CDCl_3$, 295 K) of $3b_3$**.**

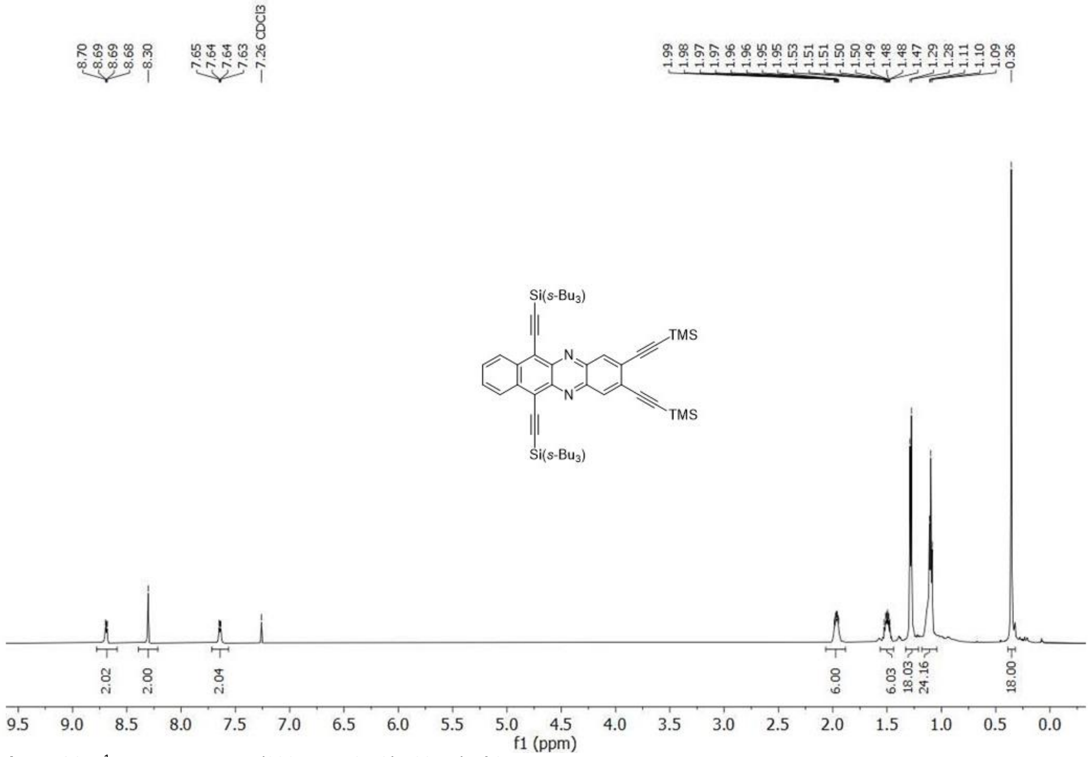

Figure S24. ^{1}H NMR spectrum (600 MHz, $CDCl_3$, 295 K) of **2c.**

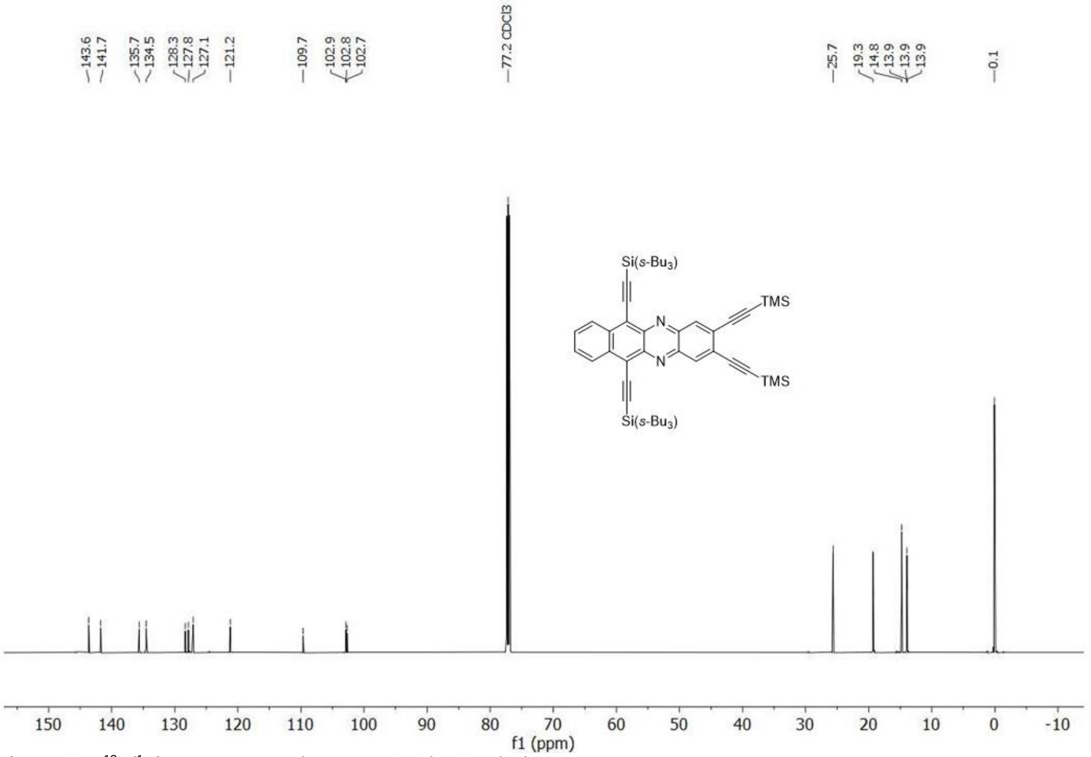

Figure S25. $^{13}C\{^{1}H\}$ NMR spectrum (151 MHz, $CDCl_3$, 295 K) of **2c.**

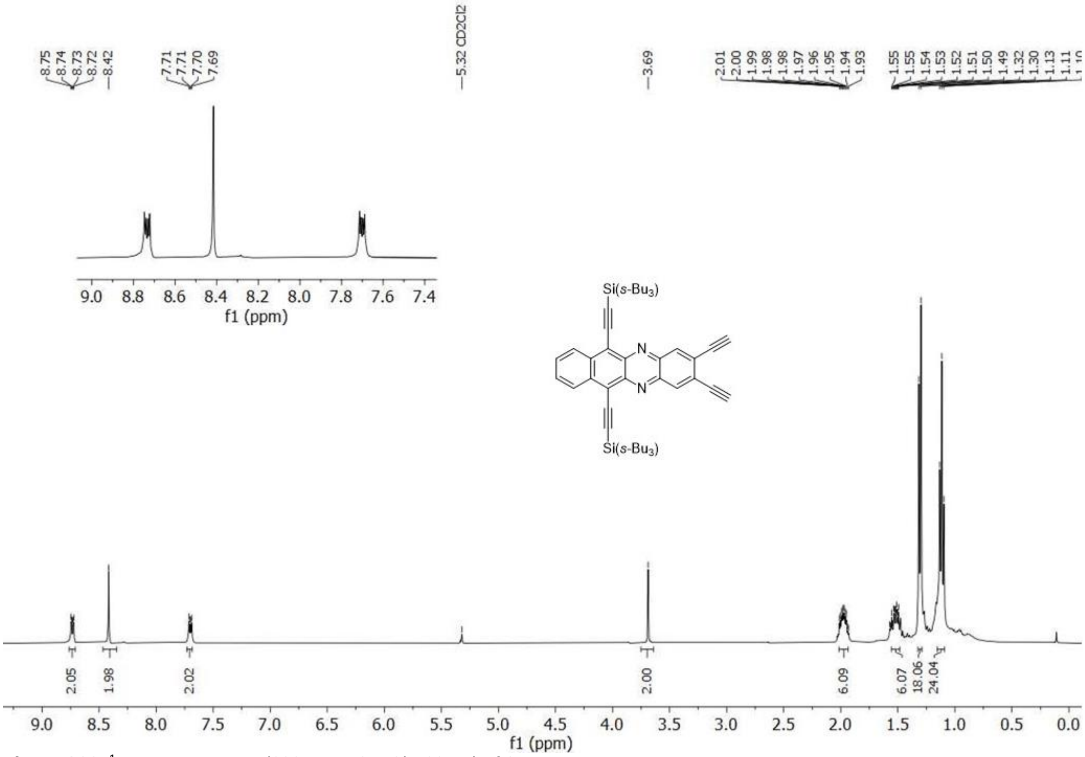

Figure S26. ^{1}H NMR spectrum (600 MHz, CD_2Cl_2, 295 K) of **3c.**

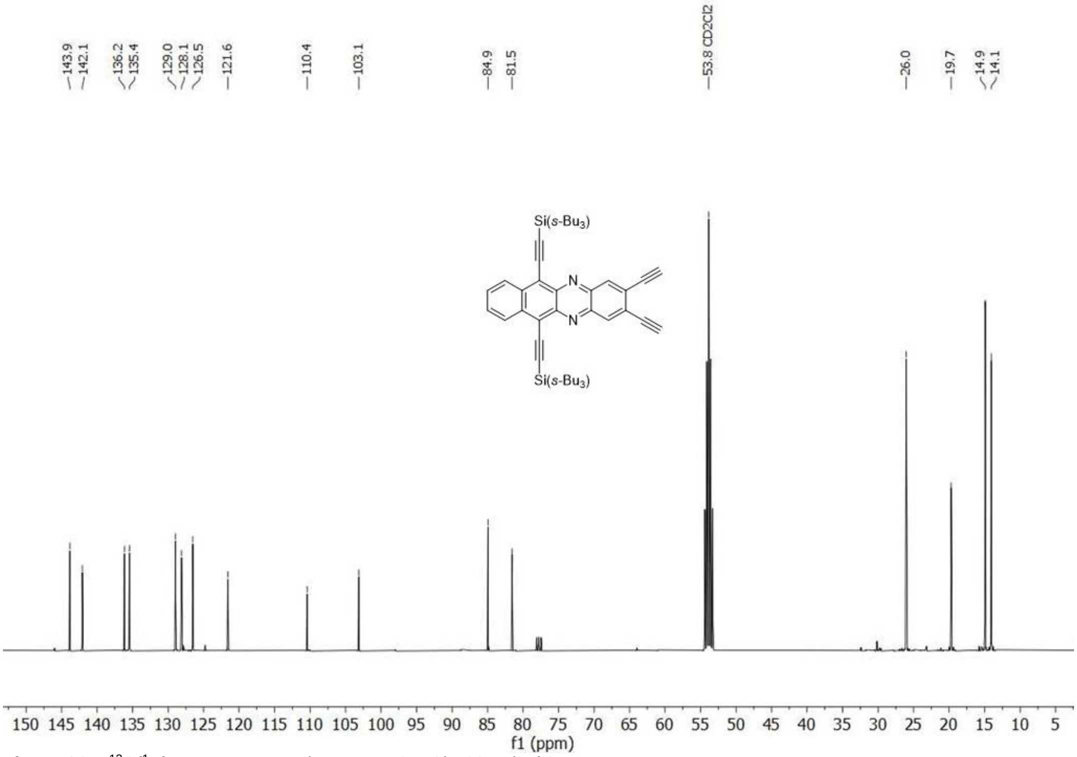

Figure S27. $^{13}C\{^{1}H\}$-NMR spectrum (151 MHz, CD_2Cl_2, 295 K) of **3c.**

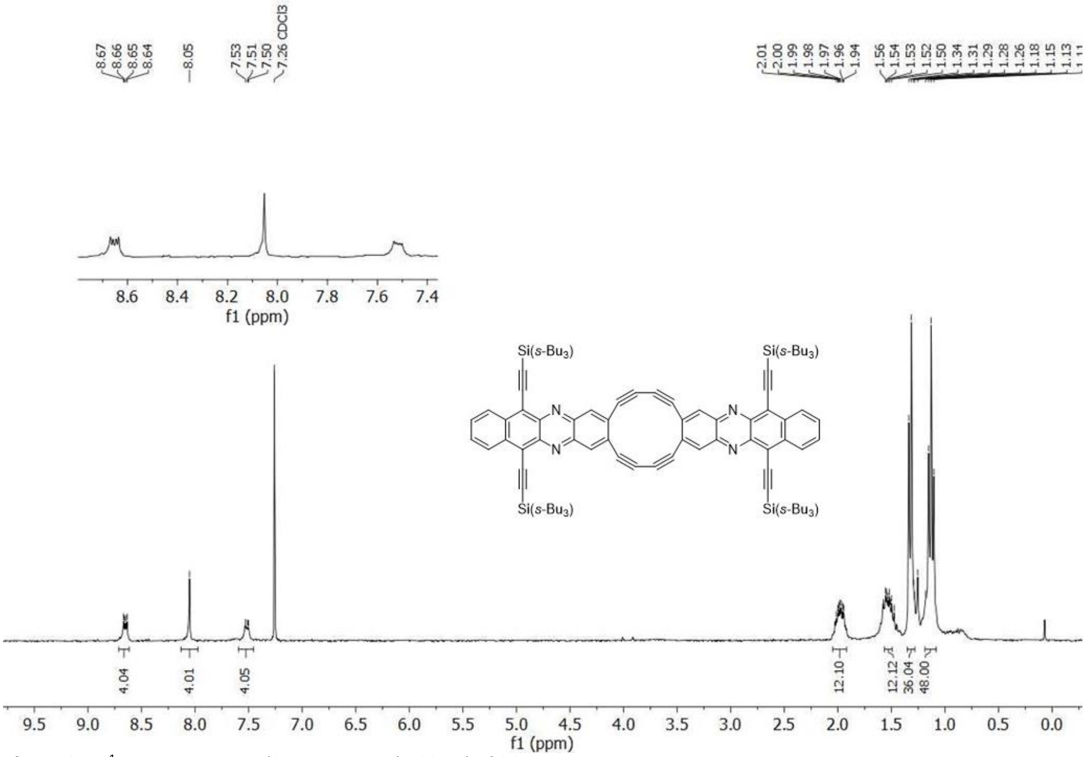

Figure S28. ^{1}H NMR spectrum (600 MHz, $CDCl_3$, 295 K) of **3C₂**.

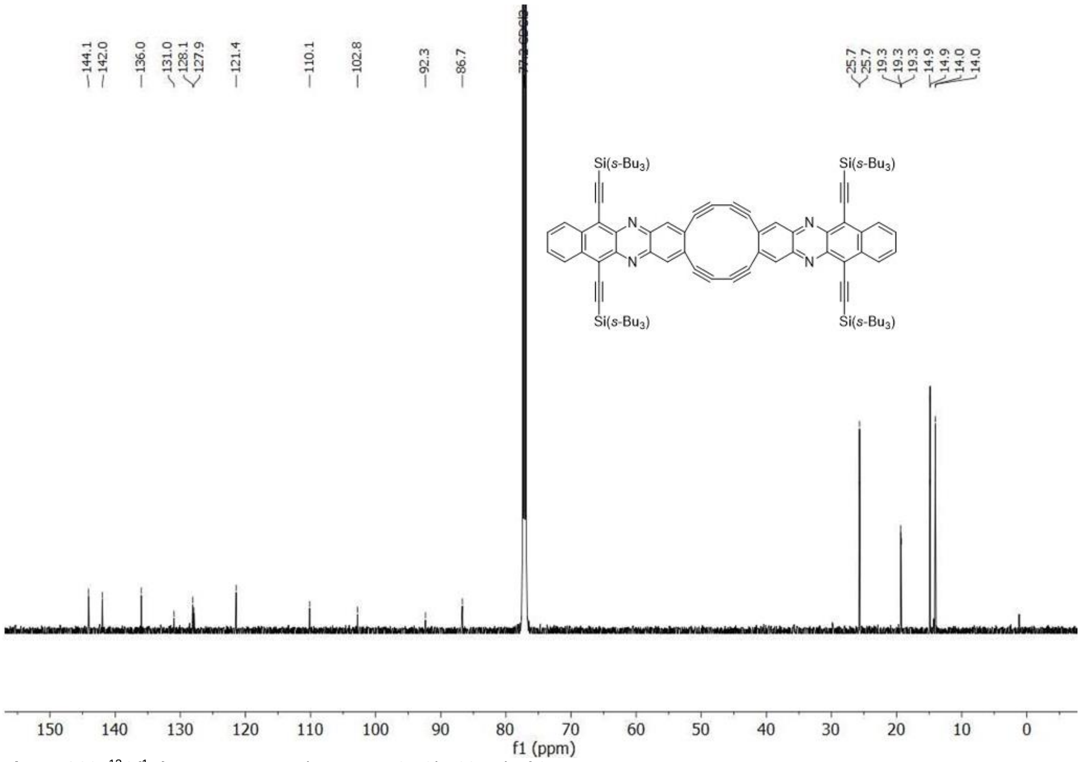

Figure S29. $^{13}C\{^{1}H\}$ NMR spectrum (151 MHz, $CDCl_3$, 295 K) of **3c₂.**

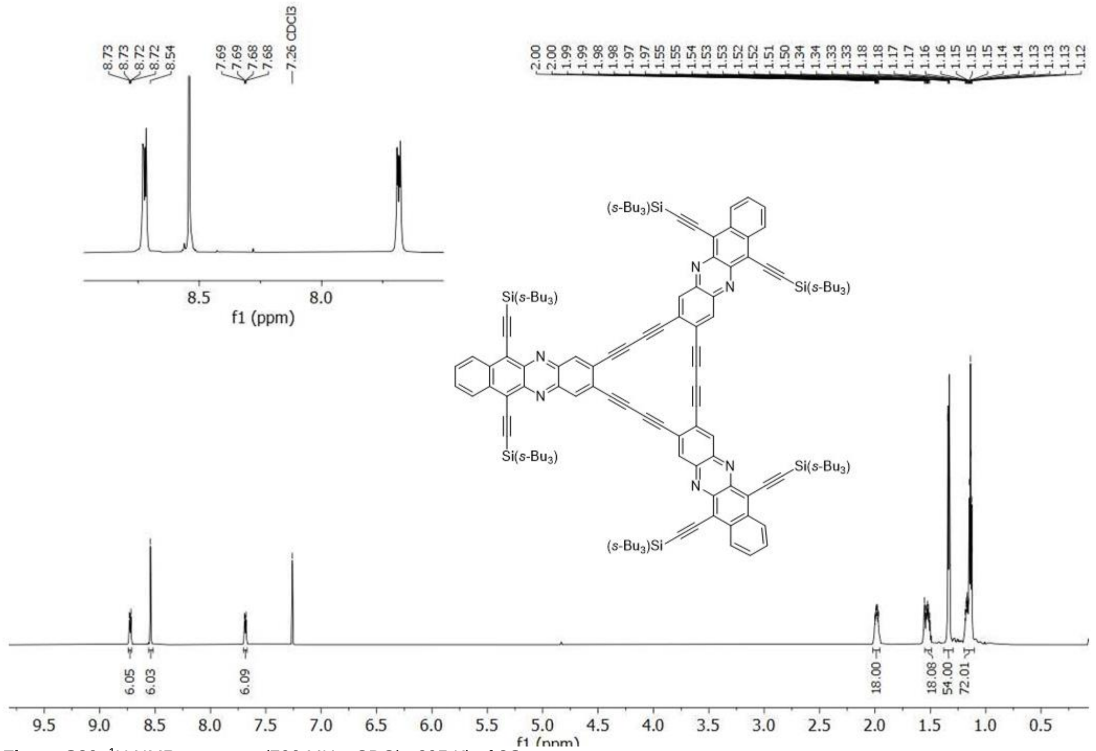

Figure S30. ^{1}H NMR spectrum (700 MHz, $CDCl_3$, 295 K) of **$3C_3$.**

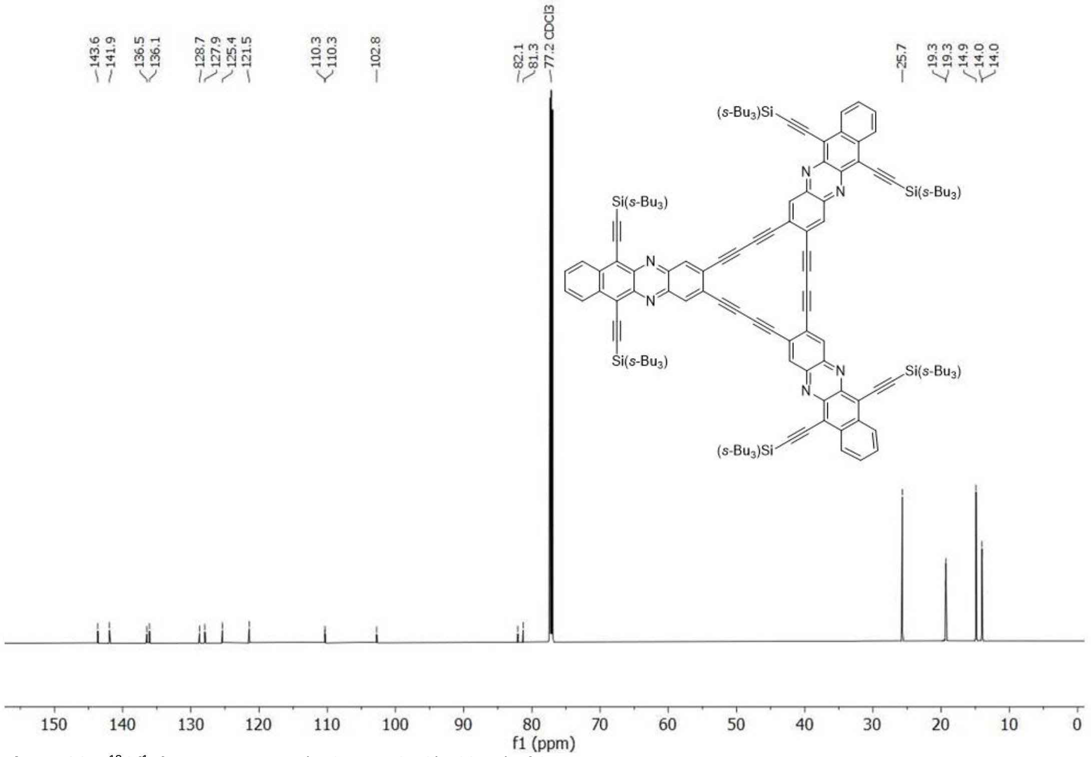

Figure S31. $^{13}C\{^{1}H\}$ NMR spectrum (176 MHz, $CDCl_3$, 295 K) of **$3c_3$.**

2.3. UV-vis spectra

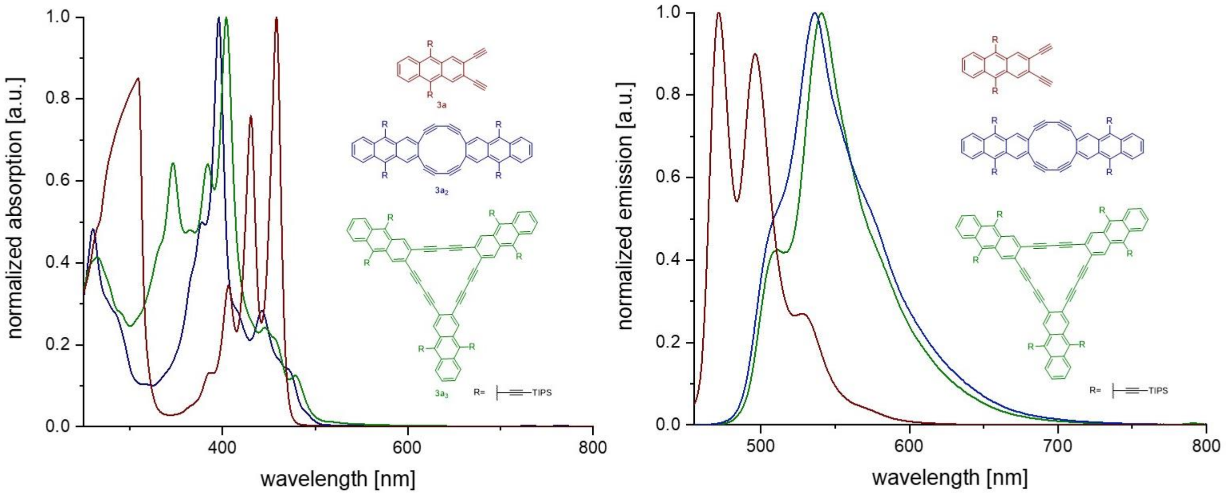

Figure S32. Normalized absorption (left) and emission (right) spectra of **3a**, **3a₂** and **3a₃** in DCM.

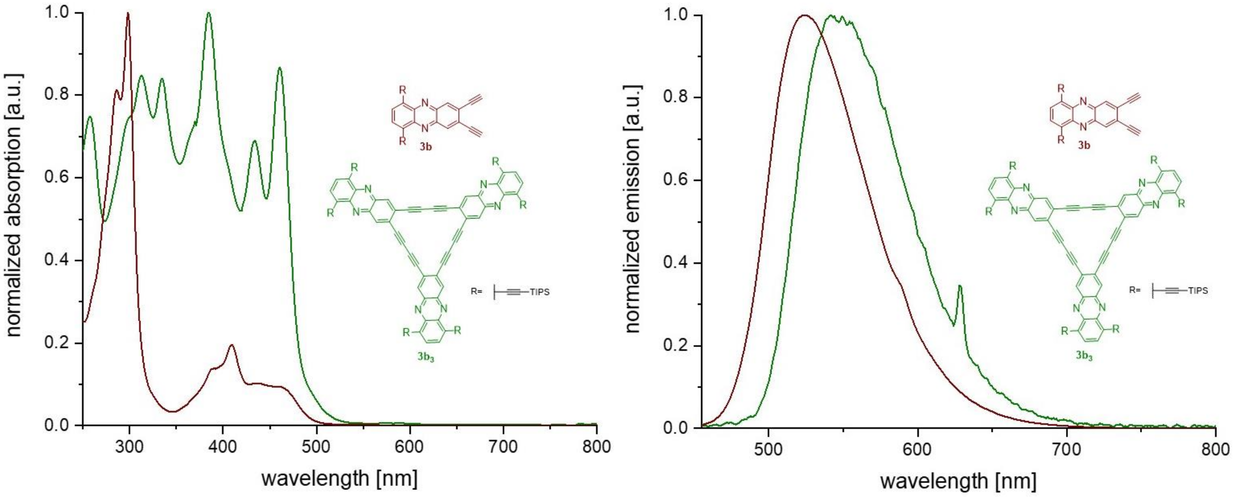

Figure S33. Normalized absorption (left) and emission (right) spectra of **3b** and **3b₂** in DCM. The peak around 640 nm is due to the double excitation wavelength.

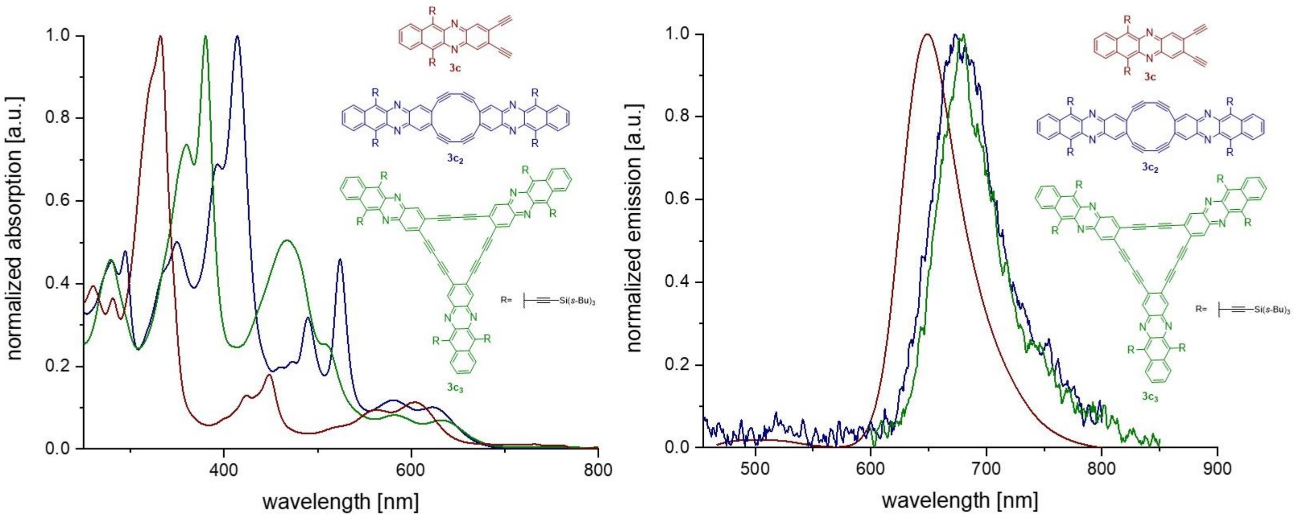

Figure S34. Normalized absorption (left) and emission (right) spectra of **3c**, **3c₂** and **3c₃** in DCM. The emission of **3c₂** and **3c₃** is very faint.

2.4 Cyclic Voltammetry

The cyclic voltammetry (CV) experiments were carried out using a platinum working electrode, a platinum wire auxiliary electrode, a silver wire reference electrode, a 0.1 mol L^{-1} NBu_4PF_6 solution in degassed, dry DCM, and ferrocene/ferrocenium as the reference redox system and internal standard (−5.1 eV)[S8] at room temperature and 0.2 V s^{-1} or 0.5 V s^{-1}. To determine the first reduction potentials ($E_{(0/-)}$) of the samples and the first oxidation potential of ferrocene, the half-wave potentials were used. For irreversible redox events, the maximum of the peak was used. For **3a$_2$** no CV spectrum could be obtainend due to low solubility.

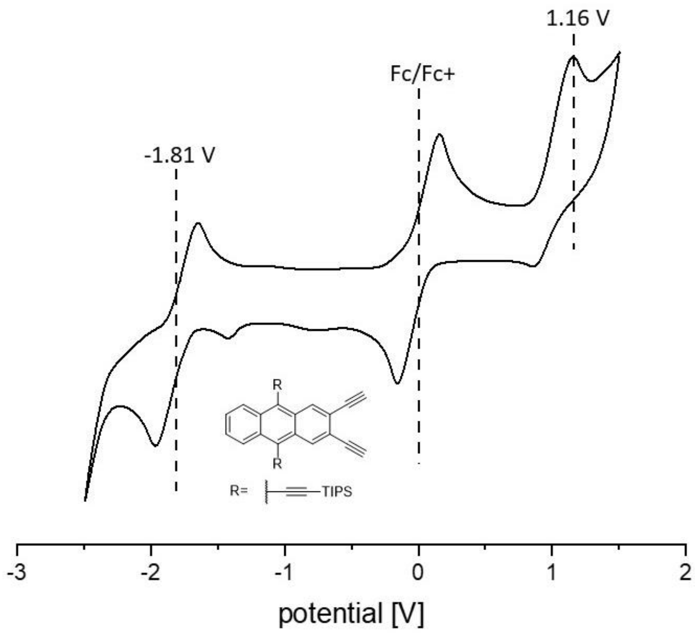

Figure S35. CV spectrum of **3a** containing ferrocene as internal standard.

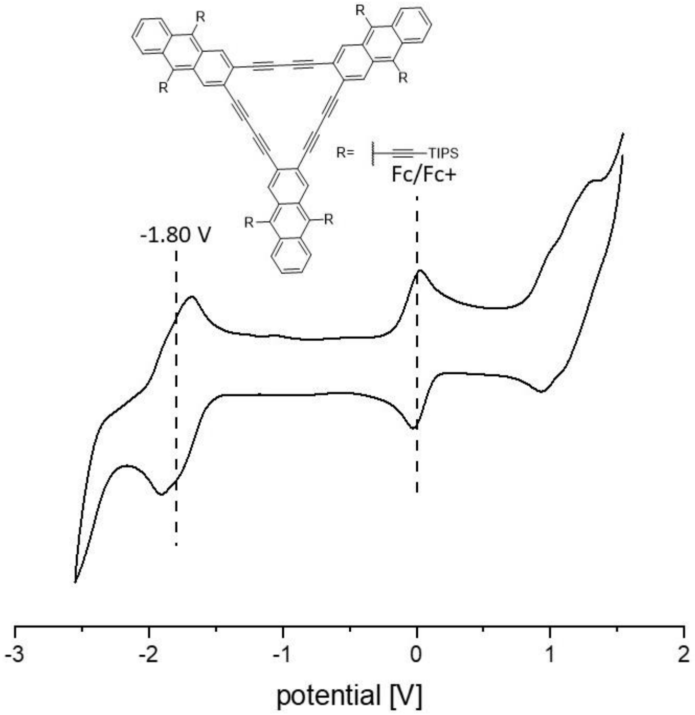

Figure S36. CV spectrum of **3a$_3$** containing ferrocene as internal standard.

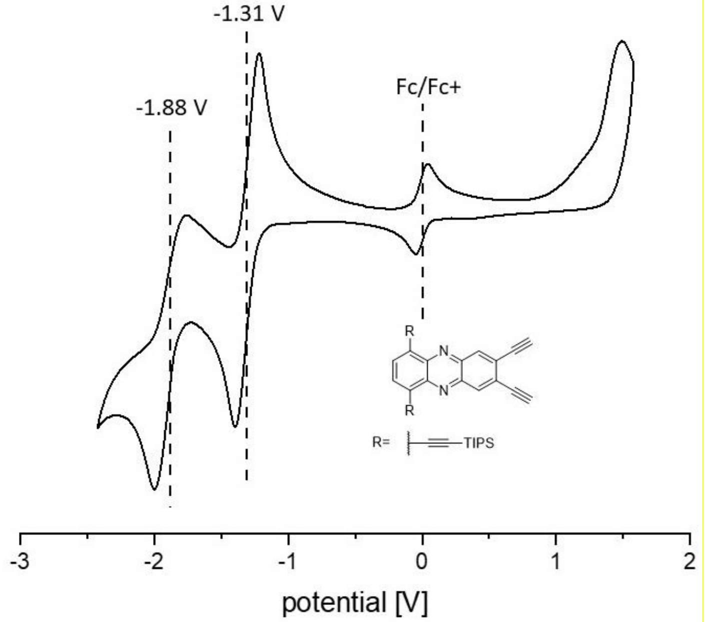

Figure S37. CV spectrum of **3b** containing ferrocene as internal standard.

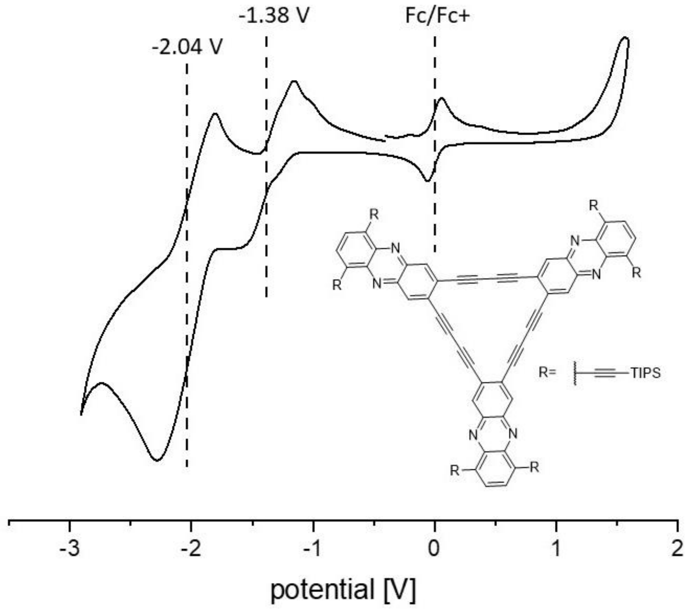

Figure S38. CV spectrum of **3b$_3$** containing ferrocene as internal standard.

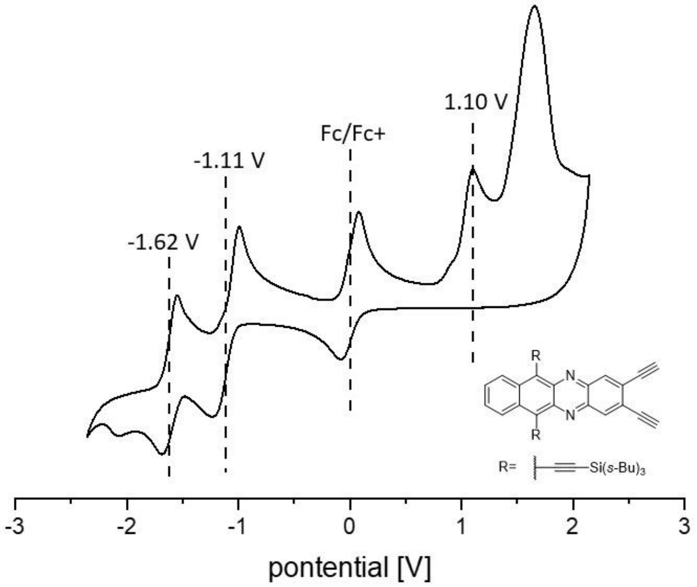

Figure S39. CV spectrum of **3c** containing ferrocene as internal standard.

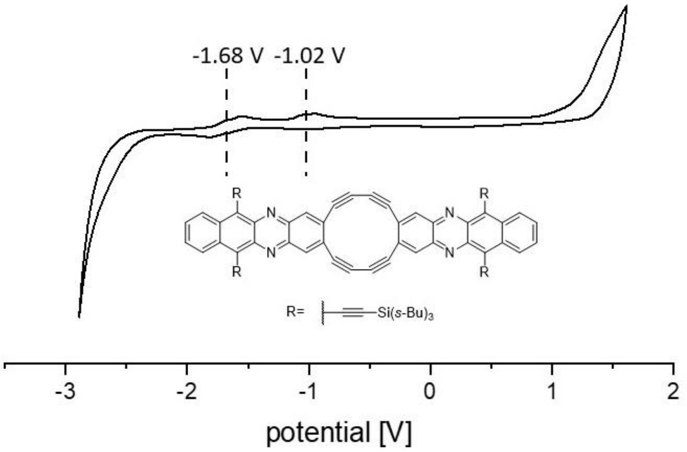

Figure S40. CV spectrum of **3c**$_2$.

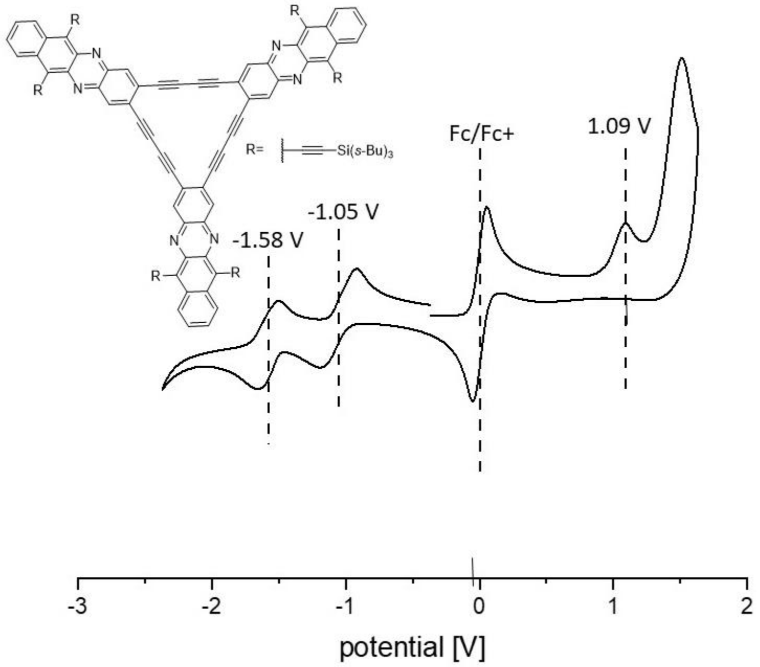

Figure S41. CV spectrum of **3c$_3$** containing ferrocene as internal standard. Here the first cycle is shown, due to decomposition on the electrode.

Table S1. Summary of the Ionization and reduction potentials. Irreversible redox events are marked with nr.

Compound	E(0/+) [eV]	E(0/-) [eV]	E(-/2-) [eV]
**3a**	1.16 (nr)	-1.81	-
**3a$_3$**	-	-1.80	-
**3b**	-	-1.31	-1.88
**3b$_3$**		-1.38	-2.04
**3c**	1.10 (nr)	-1.11	-1.62
**3c$_2$**	-	-1.02	-1.68
**3c$_3$**	1.09 (nr)	-1.05	-1.58

2.5 Hirshfeld Anaylsis

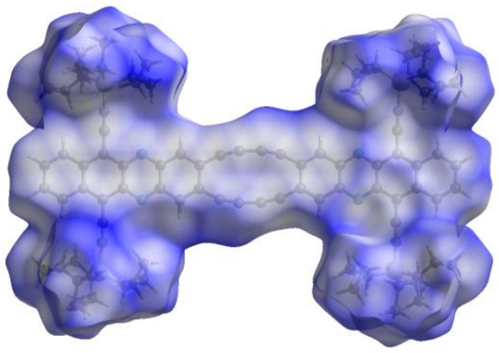

Figure S42. d_{norm} Hirshfeld surface of trimer **3c$_2$**.

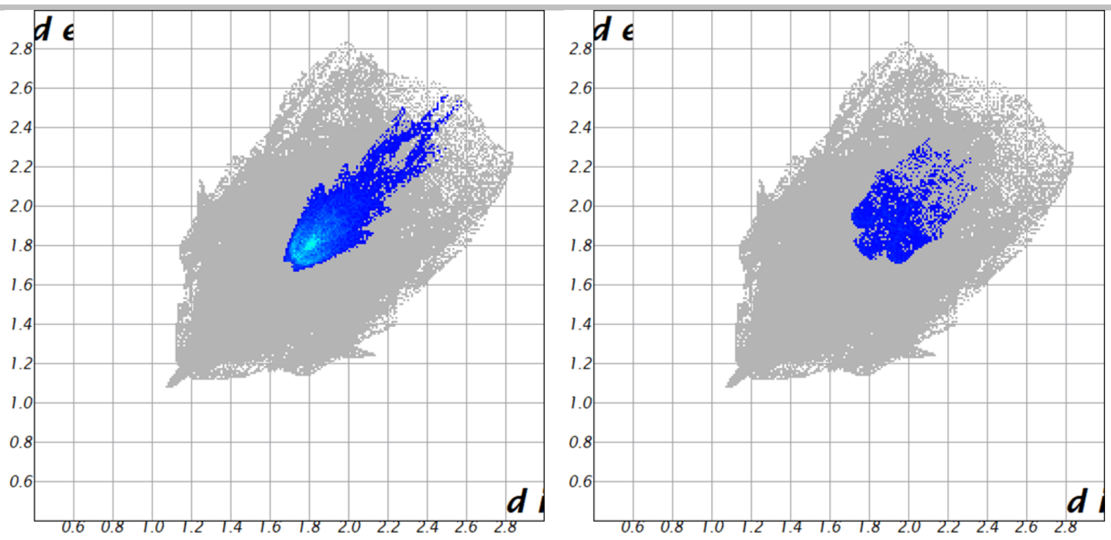

Figure S43. Fingerprint plot of trimer **3c₂**, with C-C (left) and C-N (right) contacts.

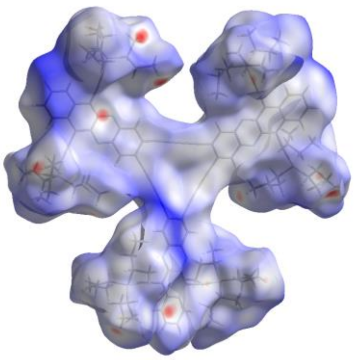

Figure S44. d_{norm} Hirshfeld surface of trimer **3c₃.**

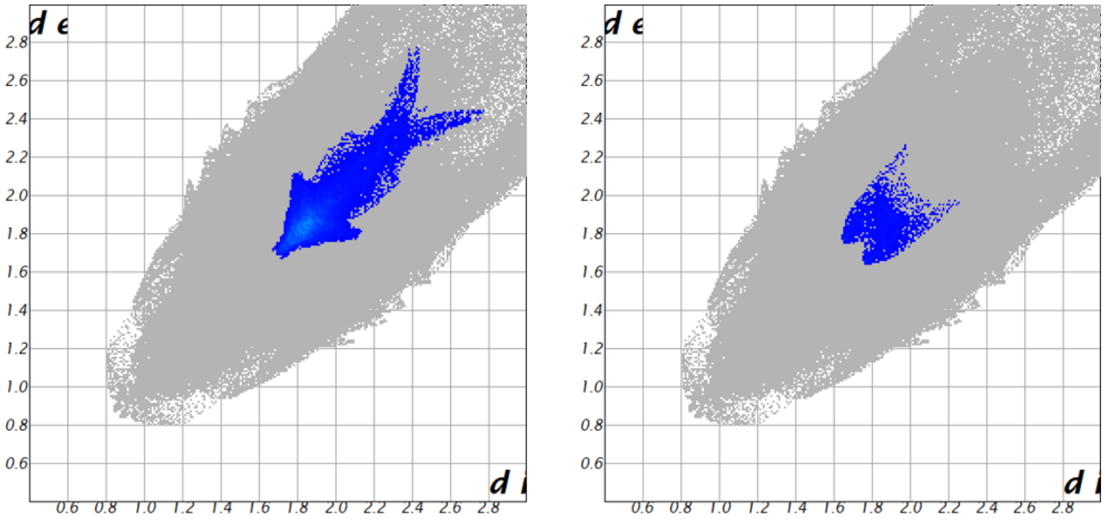

Figure S45. Fingerprint plot of trimer **3c₃**, with C-C (left) and C-N (right) contacts.

2.6 TGA/DSC Measurements

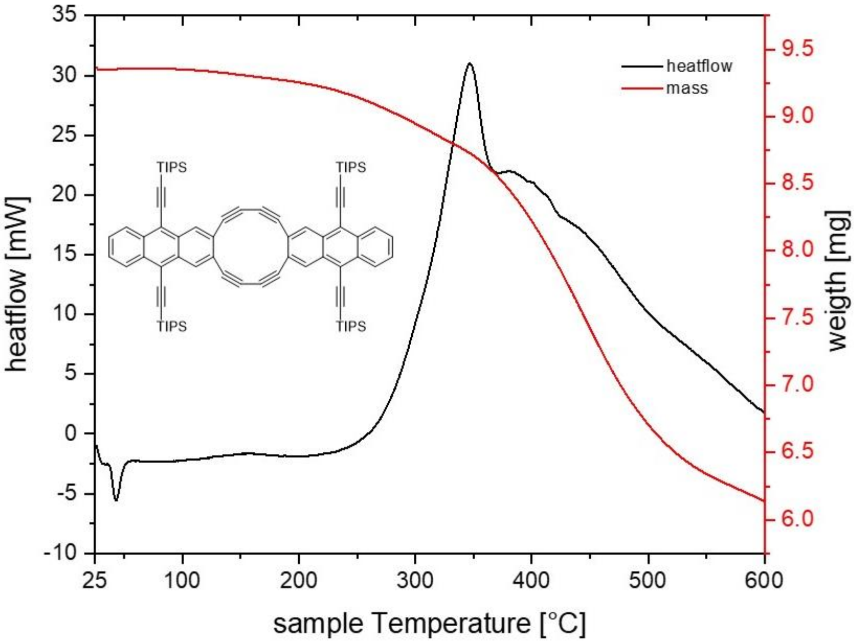

Figure S46. TGA/DSC spectrum of **3a$_2$** under a nitrogen atmosphere and a heatrate of 10 K/min.

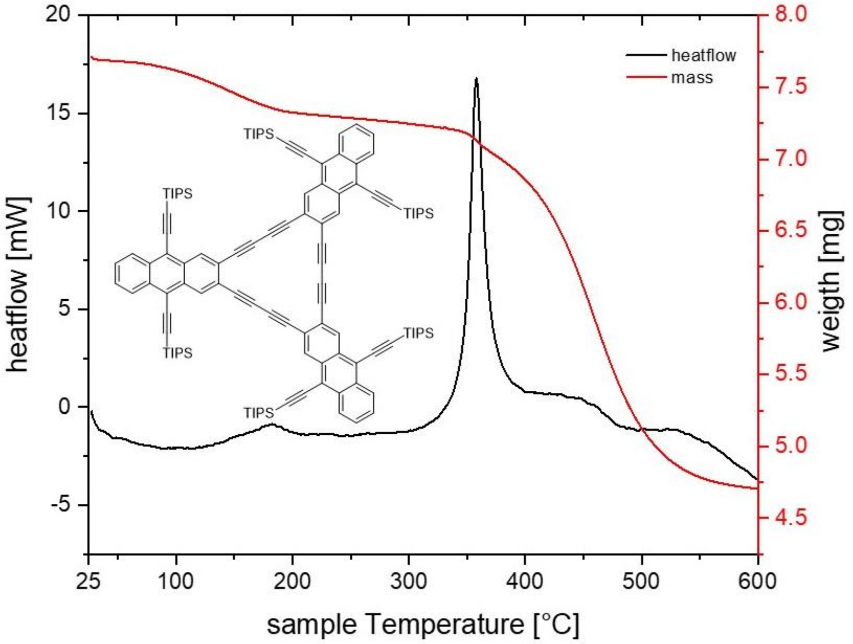

Figure S47. TGA/DSC spectrum of **3a$_3$** under a nitrogen atmosphere and a heatrate of 10 K/min.

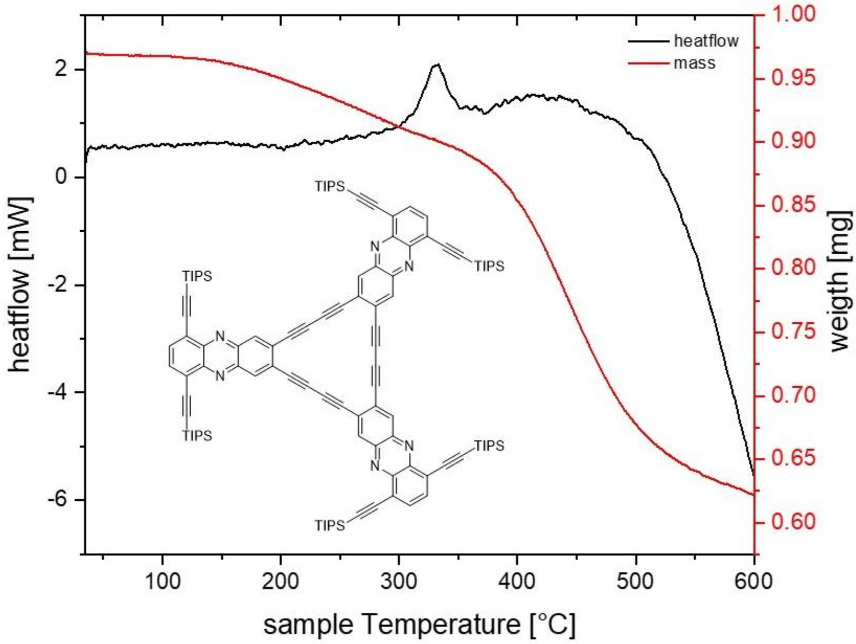

Figure S48. TGA/DSC spectrum of **3b₃** under a nitrogen atmosphere and a heatrate of 10 K/min. (The sample was drop-casted from a DCM solution into the crucible: Hence a mass lost at the beginning is probably due to remaining solvent).

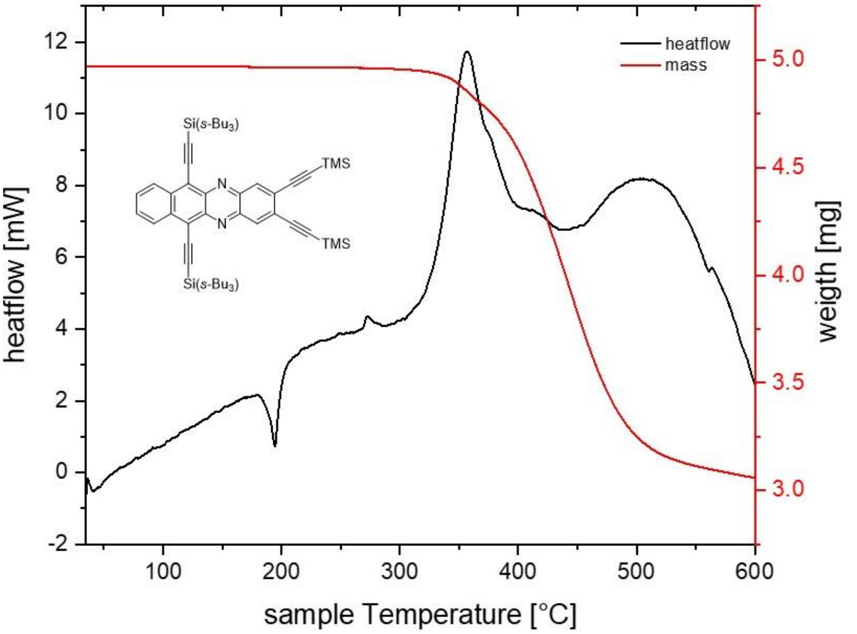

Figure S49. TGA/DSC spectrum of **2c** under a nitrogen atmosphere and a heatrate of 10 K/min.

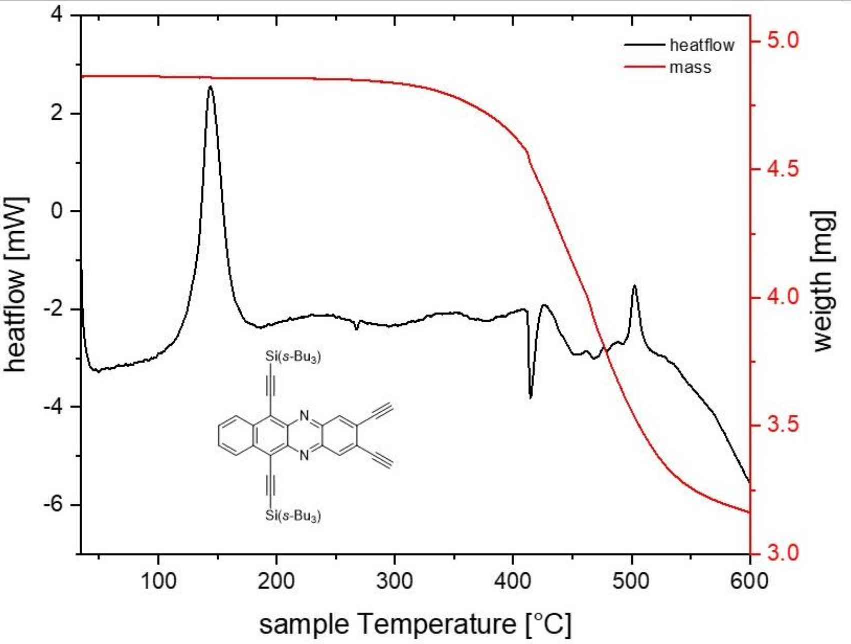

Figure S50. TGA/DSC spectrum of **3c** under a nitrogen atmosphere and a heatrate of 10 K/min.

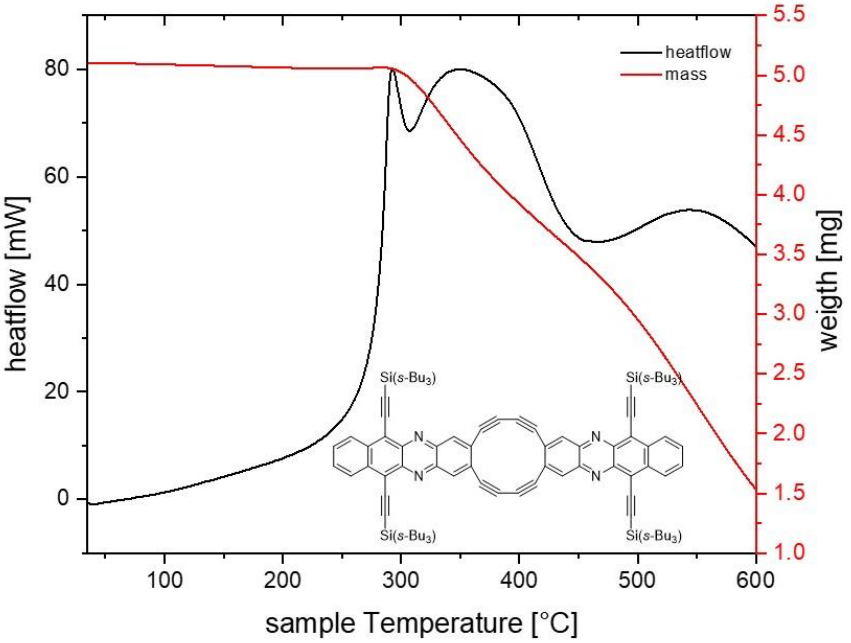

Figure S51. TGA/DSC spectrum of **3c₂** under a nitrogen atmosphere and a heatrate of 10 K/min.

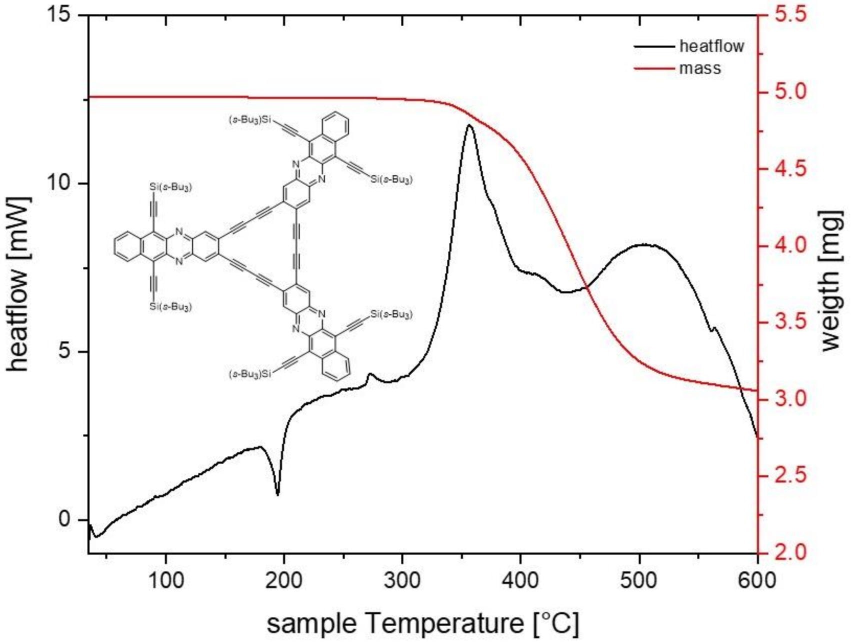

Figure S52. TGA/DSC spectrum of **3c$_3$** under a nitrogen atmosphere and a heatrate of 10 K/min.

2.7 OFET Fabrication and Measurement

A sliced, highly doped silicon wafer with 100 nm thick thermally grown SiO_2 was cleaned by ultra-sonication in acetone, isopropanol and ethanol successively for 10 min. It was washed with water and dried in a stream of nitrogen. The wafer was placed in freshly produced *Caro´s* acid and heated to 100 °C for 20 min. After cleaning with water and drying, a 150mM solution of $Al(NO_3)_3$ * 9 H_2O in ethanol was spin-coated (5000 rpm; 40 s) onto the substrate. Right after that the wafer was heated to 300 °C for 30 min. For the formation of the self-assembled monolayer, the substrate was placed in a 15.0 mM solution of 12-cyclohexyldodecylphosphonic acid (CDPA)[S9] in isopropanol for 16 h. Then the substrate was cleaned with ultra-sonication in isopropanol for 10 min, rinsed with water and dried in a stream of nitrogen. The capacitance of the dielectric layer is 26.5 nF cm^{-2}.

Drop-cast films were prepared by dropping the prepared solution (**3c$_2$**: toluene, 0.5 mg/mL; **3c$_3$**: DCM, 0.5 mg/mL) onto the substrate and covering the wafer against unwanted wind flows. To form the electrodes, a 40 nm thick layer of silver was deposited through a shadow mask onto the organic layer in a vacuum evaporator at a pressure below 2 x 10^{-6} bar. Transistor characteristics were measured with a semiconductor characterization system (Keithley 4200-SCS) in a nitrogen filled glove box. The field effect mobilities were determined in the saturated regime using the equation $\mu = \left(\frac{\partial\,(I_{DS})^{1/2}}{\partial\,V_G}\right)^2 \frac{2L}{W\,C_i}$, where I_{DS} is the source-drain current, W is the channel width, L the channel length, C_i is the capacitance per unit area of the gate dielectric layer, μ is the field effect mobility.[S10]

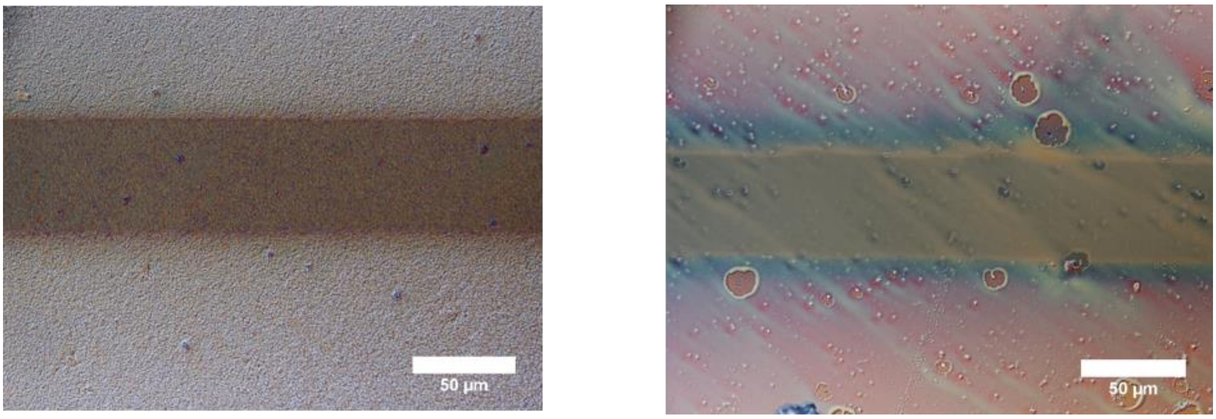

Figure S53. Exemplary measured channels of dimer **3c$_2$** (left) and trimer **3c$_3$** (right) (50x magnification, cross-polarized filters).

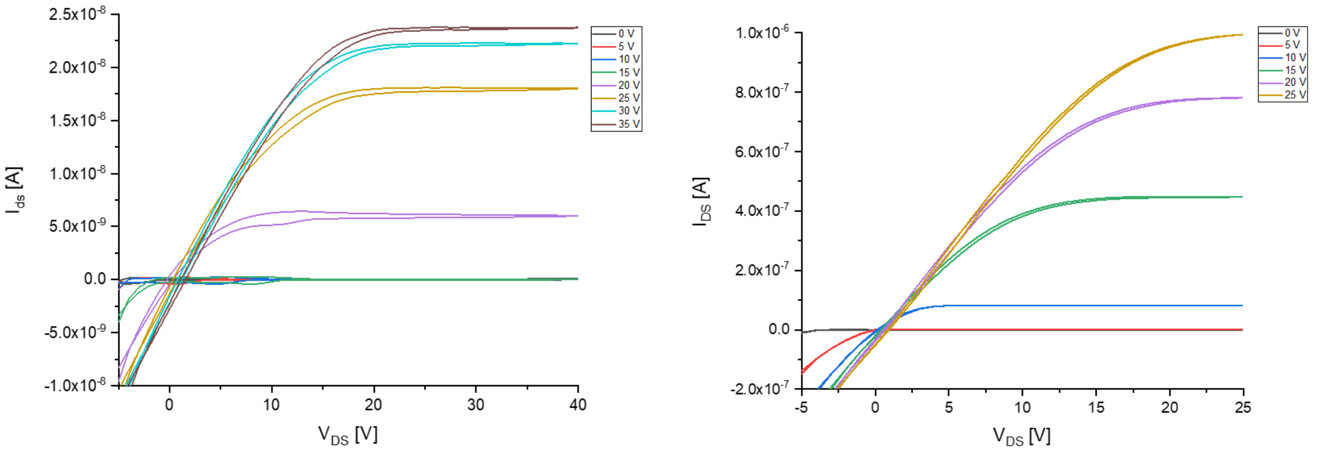

Figure S54. Output characteristics of dimer **3c₂** (left) and trimer **3c₃** (right).

2.6 Crystallographic Data

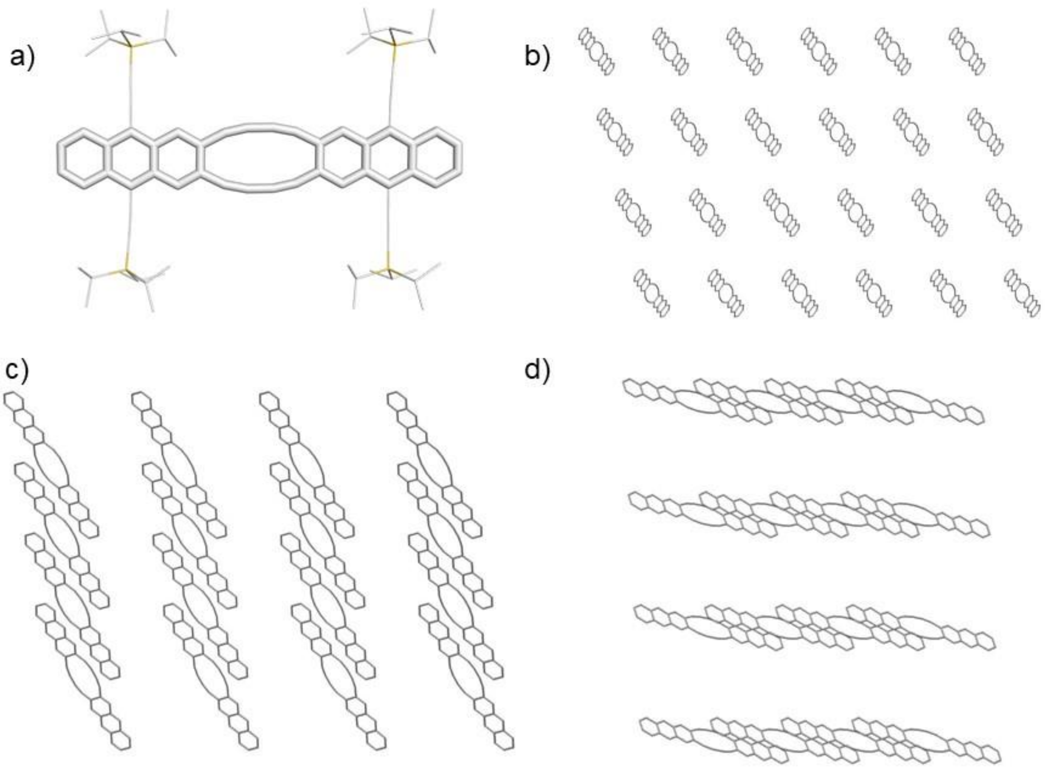

Figure S55. Single crystal Structure of **3a₂**. a) single molecule, b)packing viewed along crystallographic axis a (TIPS-ethinyl groups were removed for clarity), c) packing viewed along crystallographic axis b (TIPS-ethinyl groups were removed for clarity), d) packing viewed along crystallographic axis c (TIPS-ethinyl groups were removed for clarity).

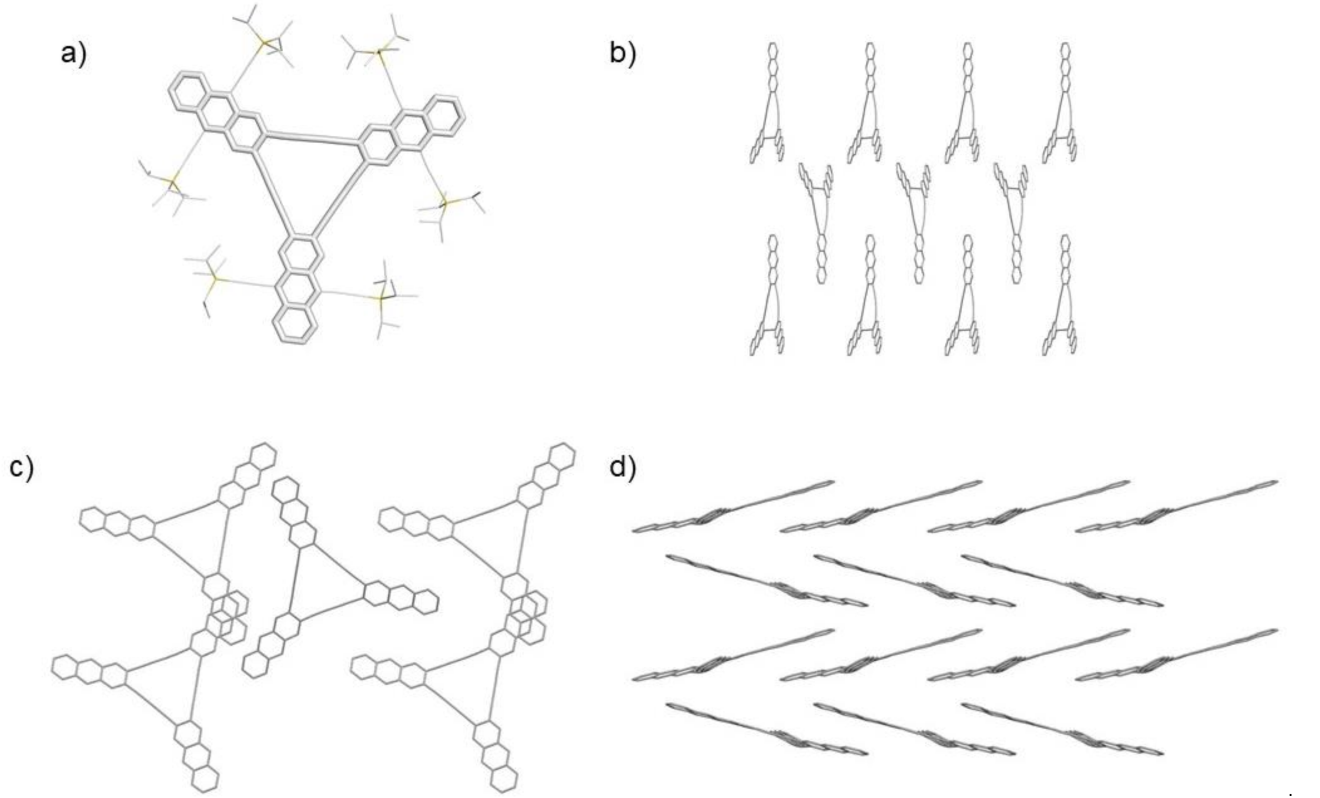

Figure S56. Single crystal Structure of **3a**$_3$. a) single molecule, b) packing viewed along crystallographic axis a (TIPS-ethinyl groups were removed for clarity), c) packing viewed along crystallographic axis b (TIPS-ethinyl groups were removed for clarity), d) packing viewed along crystallographic axis c (TIPS-ethinyl groups were removed for clarity).

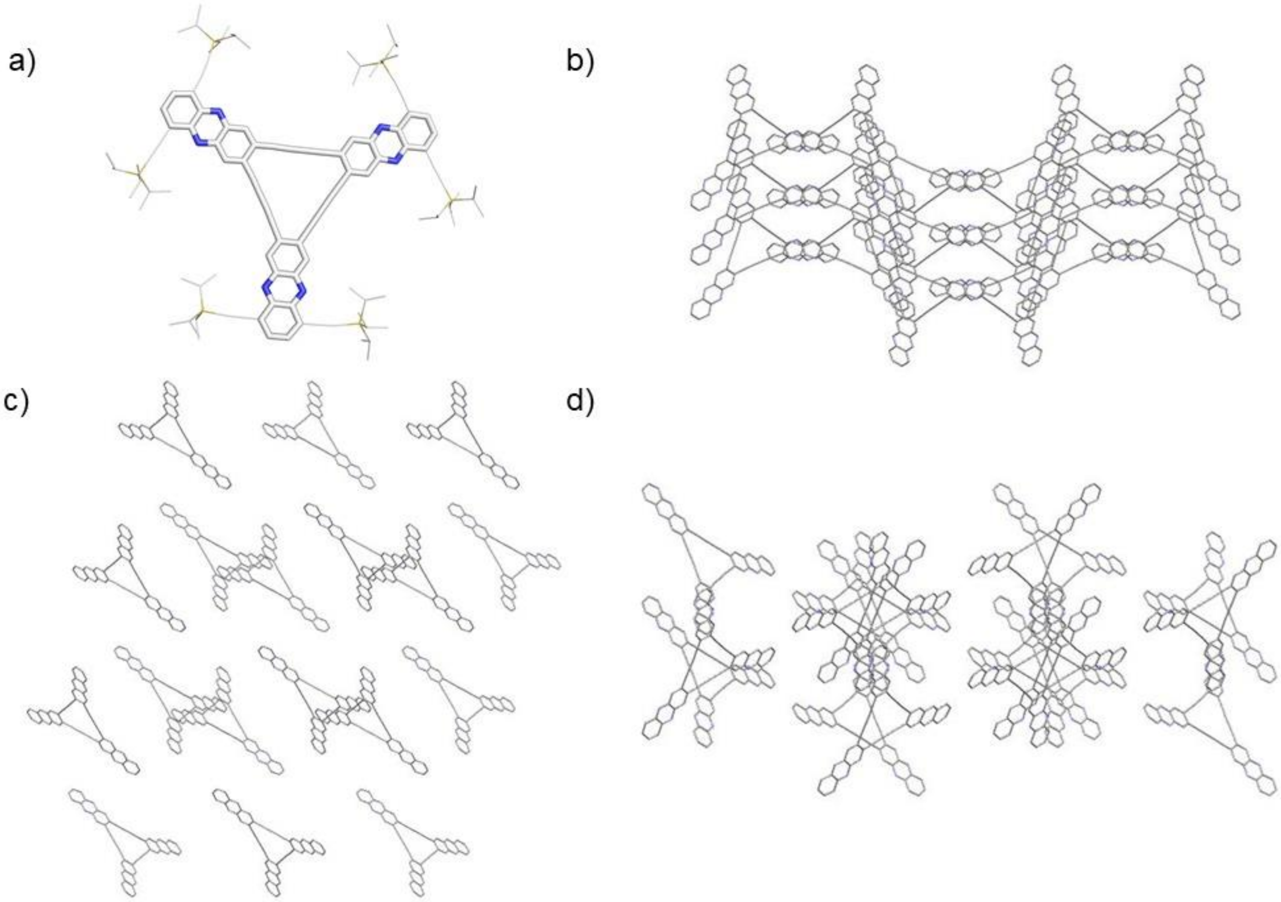

Figure S57. Single crystal Structure of **3b$_3$**. a) single molecule, b) packing viewed along crystallographic axis a (TIPS-ethinyl groups were removed for clarity), c) packing viewed along crystallographic axis b (TIPS-ethinyl groups were removed for clarity), d) packing viewed along crystallographic axis c (TIPS-ethinyl groups were removed for clarity).

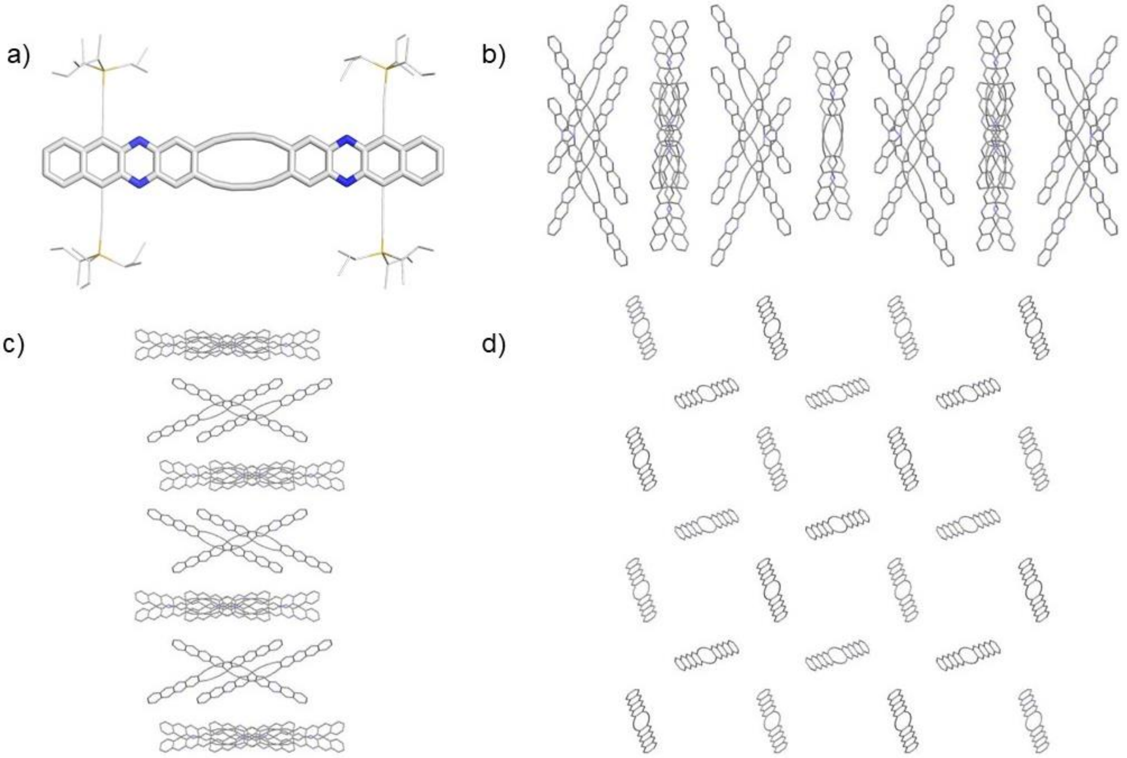

Figure S58. Single crystal Structure of **3c$_2$**. a) single molecule, b) packing viewed along crystallographic axis a (TIPS-ethinyl groups were removed for clarity), c) packing viewed along crystallographic axis b (TIPS-ethinyl groups were removed for clarity), d) packing viewed along crystallographic axis c (TIPS-ethinyl groups were removed for clarity).

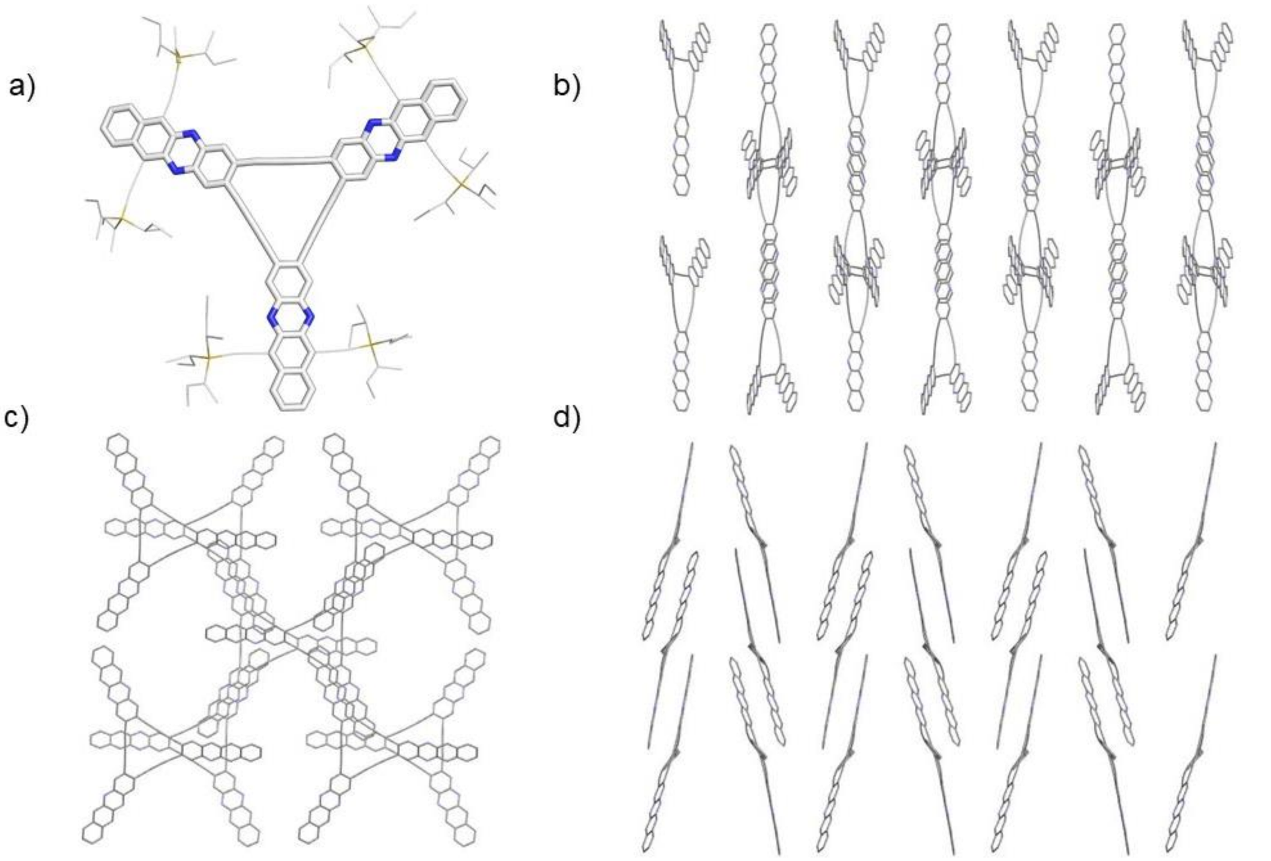

Figure S59. Single crystal Structure of **3c$_3$**. a) single molecule, b) packing viewed along crystallographic axis a (TIPS-ethinyl groups were removed for clarity), c) packing viewed along crystallographic axis b (TIPS-ethinyl groups were removed for clarity), d) packing viewed along crystallographic axis c (TIPS-ethinyl groups were removed for clarity).

Table S2. Crystal structure, crystal data and structure refinement of **3a$_2$** (CCDC 2106563).

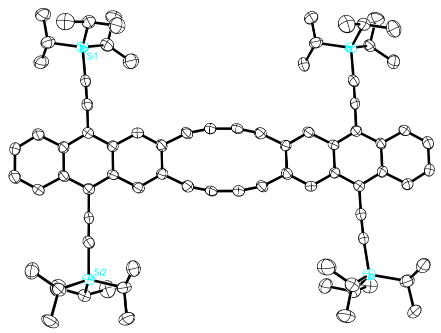

Empirical formula	$C_{80}H_{96}Si_4$	
Formula weight	1169.92	
Temperature	200(2) K	
Wavelength	1.54178 Å	
Crystal system	triclinic	
Space group	$P\bar{1}$	
Z	1	
Unit cell dimensions	a =8.4541(12) Å	α =76.569(11) deg.
	b = 13.729(2) Å	β =80.562(11) deg.
	c = 15.529(2) Å	γ =85.128(12) deg.
Volume	1727.4(4) Å^3	
Density (calculated)	1.12 g/cm^3	

Absorption coefficient	1.11 mm^{-1}
Crystal shape	needle
Crystal size	0.103 x 0.022 x 0.015 mm^3
Crystal colour	orange
Theta range for data collection	3.0 to 51.9 deg.
Index ranges	$-8\leq h\leq 6$, $-14\leq k\leq 14$, $-15\leq l\leq 15$
Reflections collected	11245
Independent reflections	3835 (R(int) = 0.1298)
Observed reflections	1778 (I > 2σ(I))
Absorption correction	Semi-empirical from equivalents
Max. and min. transmission	1.61 and 0.71
Refinement method	Full-matrix least-squares on F^2
Data/restraints/parameters	3835 / 308 / 391
Goodness-of-fit on F^2	1.01
Final R indices (I>2sigma(I))	R1 = 0.077, wR2 = 0.152
Largest diff. peak and hole	0.26 and -0.29 eÅ^{-3}

Table S3. Crystal structure, crystal data and structure refinement of **$3a_3$** (CCDC 2106564).

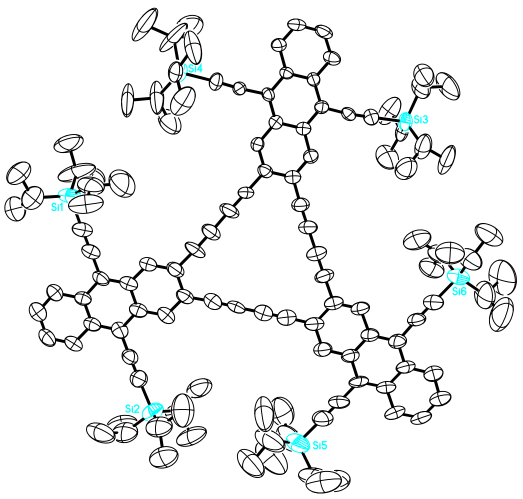

Empirical formula	$C_{122.50}H_{144.50}Cl_{7.50}Si_6$	
Formula weight	2051.29	
Temperature	200(2) K	
Wavelength	1.54178 Å	
Crystal system	monoclinic	
Space group	$P2_1$	
Z	2	
Unit cell dimensions	a = 14.7849(7) Å	α = 90 deg.
	b = 14.5900(6) Å	β =100.692(4) deg.
	c = 29.3325(14) Å	γ = 90 deg.
Volume	6217.5(5) Å^3	
Density (calculated)	1.10 g/cm^3	
Absorption coefficient	2.44 mm^{-1}	
Crystal shape	plank	
Crystal size	0.102 x 0.043 x 0.032 mm^3	
Crystal colour	orange	
Theta range for data collection	3.1 to 45.5 deg.	
Index ranges	$-13\leq h\leq 13$, $-12\leq k\leq 13$, $-27\leq l\leq 27$	
Reflections collected	61444	
Independent reflections	8818 (R(int) = 0.2018)	
Observed reflections	5041 (I > 2σ(I))	
Absorption correction	Semi-empirical from equivalents	
Max. and min. transmission	1.57 and 0.58	
Refinement method	Full-matrix least-squares on F^2	
Data/restraints/parameters	8818 / 3406 / 1245	
Goodness-of-fit on F^2	1.03	
Final R indices (I>2sigma(I))	R1 = 0.084, wR2 = 0.202	
Absolute structure parameter	0.11(7)	
Largest diff. peak and hole	0.39 and -0.26 eÅ^{-3}	

Table S4. Crystal structure, crystal data and structure refinement of **$3b_3$** (CCDC 2106565).

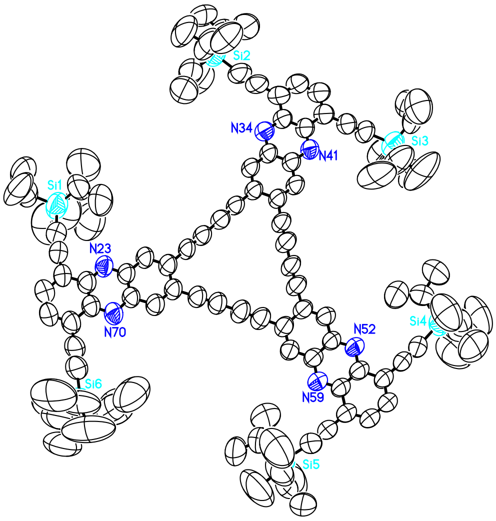

Empirical formula	$C_{114}H_{138}N_6Si_6$	
Formula weight	1760.84	
Temperature	200(2) K	
Wavelength	1.54178 Å	
Crystal system	monoclinic	
Space group	C2/c	
Z	8	
Unit cell dimensions	a = 43.659(2) Å	α = 90 deg.
	b = 13.3478(5) Å	β =107.747(3) deg.
	c = 41.943(3) Å	γ = 90 deg.
Volume	23279(2) $Å^3$	
Density (calculated)	1.00 g/cm^3	
Absorption coefficient	1.00 mm^{-1}	
Crystal shape	plank	
Crystal size	0.420 x 0.108 x 0.052 mm^3	
Crystal colour	orange	
Theta range for data collection	2.1 to 42.5 deg.	
Index ranges	$-38 \le h \le 37$, $-11 \le k \le 5$, $-36 \le l \le 36$	
Reflections collected	28438	
Independent reflections	8048 (R(int) = 0.0674)	
Observed reflections	4128 (I > 2σ(I))	
Absorption correction	Semi-empirical from equivalents	
Max. and min. transmission	1.48 and 0.65	
Refinement method	Full-matrix least-squares on F^2	
Data/restraints/parameters	8048 / 3126 / 1135	
Goodness-of-fit on F^2	1.23	
Final R indices (I>2sigma(I))	R1 = 0.119, wR2 = 0.326	
Largest diff. peak and hole	0.42 and -0.27 $eÅ^{-3}$	

Table S5. Crystal structure, crystal data and structure refinement of **3c$_2$** (CCDC 2106566).

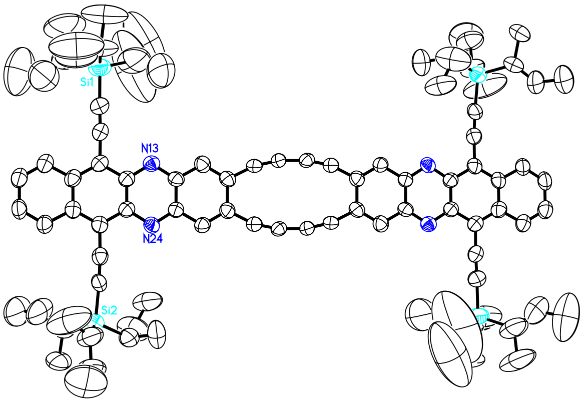

Identification code	mai48		
Empirical formula	$C_{96}H_{120}N_4Si_4$		
Formula weight	1442.31		
Temperature	200(2) K		
Wavelength	1.54178 Å		
Crystal system	tetragonal		
Space group	$I4_1/a$		
Z	8		
Unit cell dimensions	a = 45.2457(11) Å	α =	90 deg.
	b = 45.2457(11) Å	β =	90 deg.
	c =8.5618(3) Å	γ =	90 deg.
Volume	17527.5(10) $Å^3$		
Density (calculated)	1.09 g/cm^3		
Absorption coefficient	0.97 mm^{-1}		
Crystal shape	plank		
Crystal size	0.226 x 0.018 x 0.016 mm^3		
Crystal colour	red		
Theta range for data collection	2.8 to 49.7 deg.		
Index ranges	$-24 \le h \le 44$, $-43 \le k \le 42$, $-8 \le l \le 4$		
Reflections collected	16239		
Independent reflections	4358 (R(int) = 0.1456)		
Observed reflections	2366 (I > 2□(I))		
Absorption correction	Semi-empirical from equivalents		
Max. and min. transmission	0.99 and 0.66		
Refinement method	Full-matrix least-squares on F^2		
Data/restraints/parameters	4358 / 568 / 474		
Goodness-of-fit on F^2	1.09		
Final R indices (I>2sigma(I))	R1 = 0.126, wR2 = 0.272		
Largest diff. peak and hole	0.37 and -0.25 $eÅ^{-3}$		

Table S6. Crystal structure, crystal data and structure refinement of **$3c_3$** (CCDC 2106567).

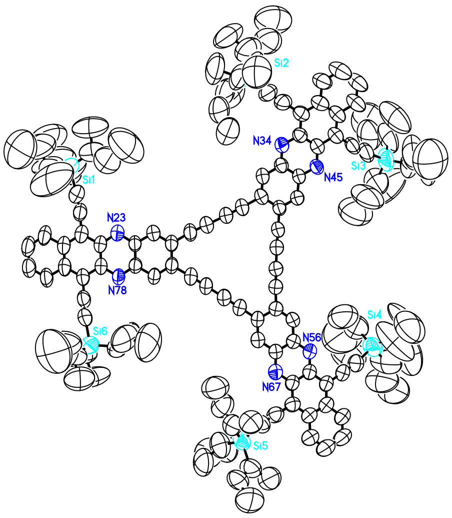

Empirical formula	$C_{144}H_{180}N_6Si_6$	
Formula weight	2163.47	
Temperature	200(2) K	
Wavelength	1.54178 Å	
Crystal system	monoclinic	
Space group	$P2_1/n$	
Z	4	
Unit cell dimensions	a = 25.8355(7) Å	α = 90 deg.
	b = 21.1688(6) Å	β =94.014(2) deg.
	c = 26.2914(9) Å	γ = 90 deg.
Volume	14343.7(7) $Å^3$	
Density (calculated)	1.00 g/cm^3	
Absorption coefficient	0.89 mm^{-1}	
Crystal shape	brick	
Crystal size	0.185 x 0.180 x 0.108 mm^3	
Crystal colour	dark green	
Theta range for data collection	3.4 to 49.1 deg.	
Index ranges	$-15 \le h \le 25$, $-20 \le k \le 20$, $-25 \le l \le 25$	
Reflections collected	57490	
Independent reflections	14037 (R(int) = 0.0452)	
Observed reflections	8924 ($I > 2\sigma(I)$)	
Absorption correction	Semi-empirical from equivalents	
Max. and min. transmission	0.92 and 0.32	
Refinement method	Full-matrix least-squares on F^2	
Data/restraints/parameters	14037 / 5524 / 1651	
Goodness-of-fit on F^2	2.31	
Final R indices (I>2sigma(I))	R1 = 0.126, wR2 = 0.326	
Largest diff. peak and hole	0.56 and -0.29 $eÅ^{-3}$	

References

[S1] G. R. Fulmer, A. J. Miller, N. H. Sherden, H. E. Gottlieb, A. Nudelman, B. M. Stoltz, J. E. Bercaw, K. I. Goldberg, *Organometallics* **2010,** *29*, 2176-2179.
[S2] Gaussian 16, Revision B.01, M. J. Frisch, G. W. Trucks, H. B. Schlegel, G. E. Scuseria, M. A. Robb, J. R. Cheeseman, G. Scalmani, V. Barone, G. A. Petersson, H. Nakatsuji, X. Li, M. Caricato, A. V. Marenich, J. Bloino, B. G. Janesko, R. Gomperts, B. Mennucci, H. P. Hratchian, J. V. Ortiz, A. F. Izmaylov, J. L. Sonnenberg, D. Williams-Young, F. Ding, F. Lipparini, F. Egidi, J. Goings, B. Peng, A. Petrone, T. Henderson, D. Ranasinghe, V. G. Zakrzewski, J. Gao, N. Rega, G. Zheng, W. Liang, M. Hada, M. Ehara, K. Toyota, R. Fukuda, J. Hasegawa, M. Ishida, T. Nakajima, Y. Honda, O. Kitao, H. Nakai, T. Vreven, K. Throssell, J. A. Montgomery, Jr., J. E. Peralta, F. Ogliaro, M. J. Bearpark, J. J. Heyd, E. N. Brothers, K. N. Kudin, V. N. Staroverov, T. A. Keith, R. Kobayashi, J. Normand, K. Raghavachari, A. P. Rendell, J. C. Burant, S. S. Iyengar, J. Tomasi, M. Cossi, J. M. Millam, M. Klene, C. Adamo, R. Cammi, J. W. Ochterski, R. L. Martin, K. Morokuma, O. Farkas, J. B. Foresman, and D. J. Fox, Gaussian, Inc., Wallingford CT, **2016**.
[S3] a) D. Geuenich, R. Herges, *J. Phys. Chem. A* **2001**, *105*, 3214-3220; b) D. Geuenich, K. Hess, F. Koehler, R. Herges, *Chem. Rev.* **2005**, *105*, 3758-3772.
[S4] U. H. F. Bunz, J. U. Engelhart, *Chem. Eur. J.* **2016**, *22*, 4680.
[S5] P. R. Spackman, M. J. Turner, J. J. McKinnon, S. K. Wolff, D. J. Grimwood, D. Jayatilaka, M. A. J. Spackman, *Appl. Cryst.* **2021**, *54*, 1006-1011.
[S6] G. te Velde, F.M. Bickelhaupt, E.J. Baerends, C. Fonseca Guerra, S.J.A. van Gisbergen, J.G. Snijders and T. Ziegler, Chemistry with ADF, *Journal of Computational Chemistry* **2001,** *22*, 931.

[S7] ADF 2021.1, SCM, Theoretical Chemistry, Vrije Universiteit, Amsterdam, The Netherlands, E.J. Baerends, T. Ziegler, A.J. Atkins, J. Autschbach, O. Baseggio, D. Bashford, A. Bérces, F.M. Bickelhaupt, C. Bo, P.M. Boerrigter, C. Cappelli, L. Cavallo, C. Daul, D.P. Chong, D.V. Chulhai, L. Deng, R.M. Dickson, J.M. Dieterich, F. Egidi, D.E. Ellis, M. van Faassen, L. Fan, T.H. Fischer, A. Förster, C. Fonseca Guerra, M. Franchini, A. Ghysels, A. Giammona, S.J.A. van Gisbergen, A. Goez, A.W. Götz, J.A. Groeneveld, O.V. Gritsenko, M. Grüning, S. Gusarov, F.E. Harris, P. van den Hoek, Z. Hu, C.R. Jacob, H. Jacobsen, L. Jensen, L. Joubert, J.W. Kaminski, G. van Kessel, C. König, F. Kootstra, A. Kovalenko, M.V. Krykunov, P. Lafiosca, E. van Lenthe, D.A. McCormack, M. Medves, A. Michalak, M. Mitoraj, S.M. Morton, J. Neugebauer, V.P. Nicu, L. Noodleman, V.P. Osinga, S. Patchkovskii, M. Pavanello, C.A. Peeples, P.H.T. Philipsen, D. Post, C.C. Pye, H. Ramanantoanina, P. Ramos, W. Ravenek, M. Reimann, J.I. Rodríguez, P. Ros, R. Rüger, P.R.T. Schipper, D. Schlüns, H. van Schoot, G. Schreckenbach, J.S. Seldenthuis, M. Seth, J.G. Snijders, M. Solà, M. Stener, M. Swart, D. Swerhone, V. Tognetti, G. te Velde, P. Vernooijs, L. Versluis, L. Visscher, O. Visser, F. Wang, T.A. Wesolowski, E.M. van Wezenbeek, G. Wiesenekker, S.K. Wolff, T.K. Woo, A.L. Yakovlev
[S8] A. Moliterni, D. Altamura, R. Lassandro, V. Olieric, G. Ferri, F. Cardarelli, A. Camposeo, D. Pisignano, J. E. Anthony, C. Giannini, *Acta Cryst*. **2020**, *B76,* 427–435.
[S9] D. Liu, Z. He, Y. Su, Y. Diao, S.C.B. Mannsfeld, Z. Bao, J. Xu, Q. Miao. Adv. Mater. **2014**, 26: 7190-7196
[S10] B. D. Lindner, F. Paulus, A. L. Appleton, M. Schaffroth, J. U. Engelhart, K. M. Schelkle, O. Tverskoy, F. Rominger, M. Hamburger, U. H. F. Bunz, *J. Mater. Chem. C* **2014**,*2*, 9609–9612
